# Photo splitting of bio-polyols and sugars to methanol and syngas

**DOI:** 10.1038/s41467-020-14915-8

**Published:** 2020-02-27

**Authors:** Min Wang, Meijiang Liu, Jianmin Lu, Feng Wang

**Affiliations:** 1 State Key Laboratory of Catalysis, Dalian National Laboratory for Clean Energy, Dalian Institute of Chemical Physics, Chinese Academy of Sciences, 116023 Dalian, Liaoning China; 2 Zhang Dayu School of Chemistry, Dalian University of Technology, 116024 Dalian, Liaoning China; 3 University of Chinese Academy of Sciences, 100049 Beijing, P. R. China

**Keywords:** Photocatalysis, Biomethanol, Chemical engineering

## Abstract

Methanol is a clean liquid energy carrier of sunshine and a key platform chemical for the synthesis of olefins and aromatics. Herein, we report the conversion of biomass-derived polyols and sugars into methanol and syngas (CO+H_2_) via UV light irradiation under room temperature, and the bio-syngas can be further used for the synthesis of methanol. The cellulose and even raw wood sawdust could be converted into methanol or syngas after hydrogenolysis or hydrolysis pretreatment. We find Cu dispersed on titanium oxide nanorod (TNR) rich in defects is effective for the selective C−C bond cleavage to methanol. Methanol is obtained from glycerol with a co-production of H_2_. A syngas with CO selectivity up to 90% in the gas phase is obtained via controlling the energy band structure of Cu/TNR.

## Abstract (web summary)

Methanol and syngas are important fuels and chemicals, which are presently produced from fossil resources. Here, the authors report the conversion of biomass-derived polyols and sugars into methanol and syngas via UV light irradiation under room temperature.

## Introduction

Exploring renewable and clean energy as alternatives for fossil fuel has aroused a great research interest in sustainable development. Methanol is recognized to be the most promising clean and large scale deployment liquid fuel in the future because it has been identified as a viable alternative liquid fuel to gasoline and diesel, which can use existing global supply chains for storage, shipping, and distribution^1^. Moreover, methanol is a fundamental chemical material and has been industrially used for the production of ethylene and propylene and also promising for the synthesis of aromatics and gasoline^2^. Currently, methanol is industrially produced from natural gas and coal^3^. The production of methanol from renewable and abundant carbon resource instead of from fossils is a promising route for both fuel and chemicals purpose^4,5^.

Biomass, with global production of 170 billion metric tons per year, store the solar energy in the chemical bonds via plants biological photosynthesis, but cannot be directly used as a liquid fuel^4,6^. The conversion of biomass into fuels have been widely studied^4,7–10^. The conversion of biomass into bio-methanol, called as liquid sunshine^1^, efficiently connects the biorefinery processes and the existing petrochemical-fuel chains. The production of bio-methanol was fundamentally investigated via bio-derived syngas^11,12^. Syngas itself is a very important platform chemical and can be converted into olefins and aromatics^13,14^, but it is challenging to selectively break up all the robust C−C bond over C−O bond to generate CO from biomass. Traditionally, biomass gasification via pyrolysis, partial oxidation or reforming at high temperature (700–1000 °C) usually generates a mixture of CO, CO_2_, hydrocarbons, and deficient H_2_ along with the formation of coke, char and tar^4,15^. The generation of a large amount of CO_2_ and hydrocarbons not only lowers the carbon atom economy but also affects the end usage of the bio-syngas. A catalytic thermal reforming has been reported for selective conversion of biomass into syngas, but still needs to be operated at a temperature higher than 300 °C and limited to the C2–C3 based polyols, such as glycerol and ethylene glycol^16,17^. Cellulose was recently reported being converted into syngas under low temperature (70 °C) via stoichiometric oxidation with polyoxometalate followed by electrolysis, but using concentrated H_2_SO_4_ solvent^18^.

Compared to indirect production of methanol from bio-syngas, direct production of methanol from biomass is more difficult and has been rarely explored^19^. It is challenging to only break up the robust C−C bond but not affect the C−H/C−O bonds. Only C2–C3 based polyols, such as glycerol and ethylene glycol, were studied but suffered from low efficiency. Tsang et al. reported the hydrogenolysis of ethylene glycol (EG) to methanol over Pd/Fe_2_O_3_ with about 6% yield^20^. Hutchings et al. realized the H_2_-free production of methanol from glycerol reforming over simple basic oxide with 40–60% yield at above 300 °C^21^.

**Fig. 1:**
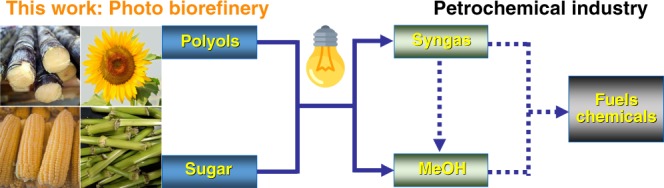
A concept of bridging biorefinery and petrochemical industry. Methanol and syngas act as the platform chemical connecting the biorefinery and petrochemical industry.

In this work, we propose a photo splitting method for the conversion of biomass into syngas and methanol (Fig. 1). We find Cu dispersed on titanium oxide nanorod (Cu/TNR) is effective for the conversion of polyols and sugars into syngas and methanol under UV light irradiation at room temperature. The gas product can be facially tuned from CO_2_ to CO via controlling the energy band structure of Cu/TNR. The cellulose and even raw wood sawdust could be converted into methanol or syngas after hydrogenolysis or hydrolysis pretreatment.

## Results

### Photoreforming of glycerol

**Fig. 2:**
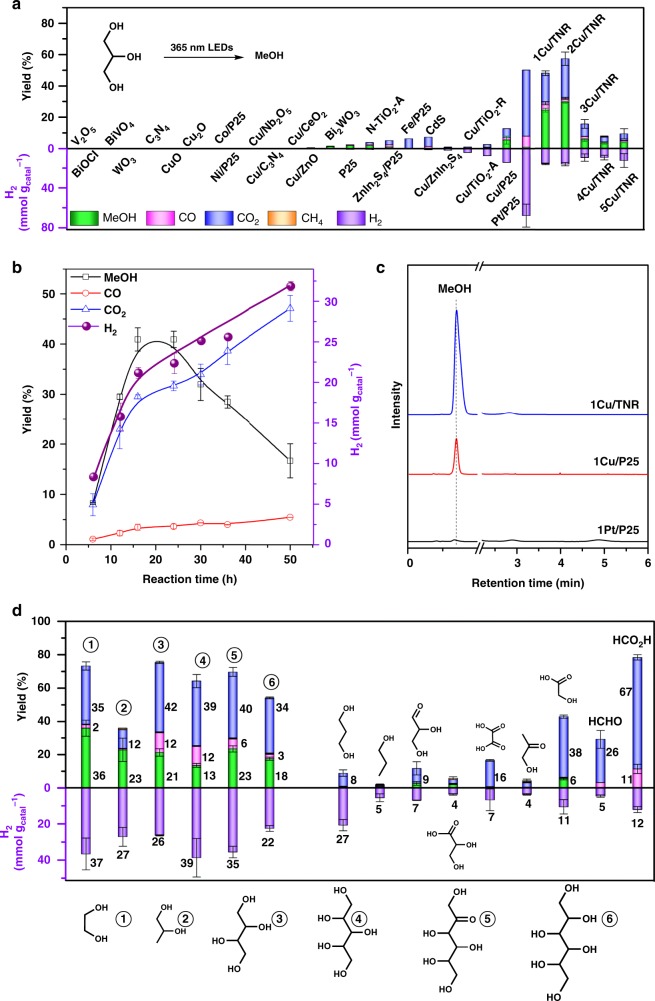
Conversion of biomass resources to methanol. **a** Catalyst screening. Reaction conditions: 10 mg of glycerol, 10 mg of catalyst, 0.8 mL of MeCN, 0.2 mL of water, 365 nm LED (18 W, 55 mW cm^−2^) irradiation for 12 h. **b** The effect of reaction time. **c** GC curves of liquid phase products. **d** Photo-reforming of different oxygenates. Detailed reaction conditions and product analysis are presented in Supplementary Tables 2, 3. Error bar represents the standard deviation.

Catalyst screening. Glycerol is a by-product in the production of biodiesel at roughly one-tenth the mass of the biodiesel. Well-utilization of the glycerol has become a bottleneck for the biodiesel technology and is providing a brake on further development^22^. We firstly investigated the photo-reforming of glycerol. Direct cleavage of the C−C bond via conventional thermal-catalysis requires to be performed at a high temperature accompanied by the risk of over decomposition and carbonization. Light-driven photocatalysis is a promising strategy to realize the C−C bond cleavage under mild conditions but the over oxidation generally occurs. Besides some works on the generation of useful chemicals from photoreforming of biomass resources^23,24^, most of the carbon are eventually converted into CO_2_ (A summary of photoreforming of glycerol was shown Supplementary Table 1). It is a challenge to selectively cleave C−C bond and avoid the over-oxidation reactions. We firstly screened a series of semiconductor photocatalysts in the photo-reforming of glycerol in MeCN-water (8:2) solution under 365 nm LED irradiation (Fig. 2a). Most of the widely used catalysts were inactive for this reaction. A trace amount of methanol was found in the case of P25. The loading of Fe, Co and Ni on P25 did not improve the catalytic performance, and methanol was not detected. When copper was loaded in the TiO_2_-R, TiO_2_-A, P25, CO_2_ was generated as the major product. Pt/P25 is very active but generates a large amount of CO_2_ and H_2_. In comparison, immobilization of copper on titanium oxide nanorod (1Cu/TNR) prefers to produce methanol, and 30% yield of methanol were produced along with a generation of 15 mmol g_cat._^−1^ of H_2_. We found MeCN is the best co-solvent in combination with H_2_O (Supplementary Fig. 1). The volume ratio of MeCN to H_2_O affects catalytic performance (Supplementary Fig. 2). The ratio of MeCN to H_2_O with 8:2 is a suitable solvent system to achieve a high yield of methanol. The copper loading of Cu/TNR showed a significant effect on the production of methanol from glycerol (Fig. 2a and Supplementary Fig. 3). The yield of methanol firstly increases with the copper loading and reaches a maximum yield at 1–2 wt% copper loading, and then declines with further increase in the content of copper beyond 2 wt%. As the reaction proceeded, the yield of methanol gradually increased and then decreased (Fig. 2b), indicating the decomposition of glycerol and methanol are two competitive routes. After optimization, about 40% yield of methanol is reached over 2Cu/TNR, along with 31% yield of CO_2_, 3.6% yield of CO and 22 mmol g_cat._^−1^ of H_2_. The apparent quantum yield is 3.4%. We tried to use sunlight for this reaction, but much lower activity was obtained due to the small content of UV light in the solar light. Under solar irradiation for 16 h, only 4% yield of methanol was produced along with a generation of 0.8 mmol g_cat_^−1^ h^−1^ of H_2_. In order to exclude the methanol comes from MeCN solvent. We performed the photoreaction in the absence of glycerol. Methanol was not detected and only a very minor amount of CO_2_ detected (Supplementary Fig. 4). These results demonstrate that the acetonitrile is relatively stable under the reaction conditions and methanol is not originated from MeCN degradation.

### Photoreforming of polyols and sugars

Then, we investigated the photo-reforming of other biomass resources over 2Cu/TNR (Fig. 2d). C2–C6 polyol, such as ethylene glycol (EG), 1,2-propanediol, erythritol, xylitol, and sorbitol were all facilely converted to methanol in 10–40% yields along with a generation of 20–40 mmol g_cat._^−1^ of H_2_. The polyols with longer carbon chains are more reluctant to react and need a longer reaction time (Supplementary Table 4). Fructose was converted to methanol in 23% yield. Methanol was formed from 1,2-propanediol with 22% yield, but not from 1,3-propanediol and 1-propanol or 2-propanol, indicating that molecules require the vicinal diol structure for this reaction. Although the structures of the substrates are different, the liquid products are similar which is probably due to that all the substrate are possessed of vicinal diol structure and probably undergo similar C−C bond cleavage route.

### Controlling the ratio of CO to CO_2_

**Fig. 3:**
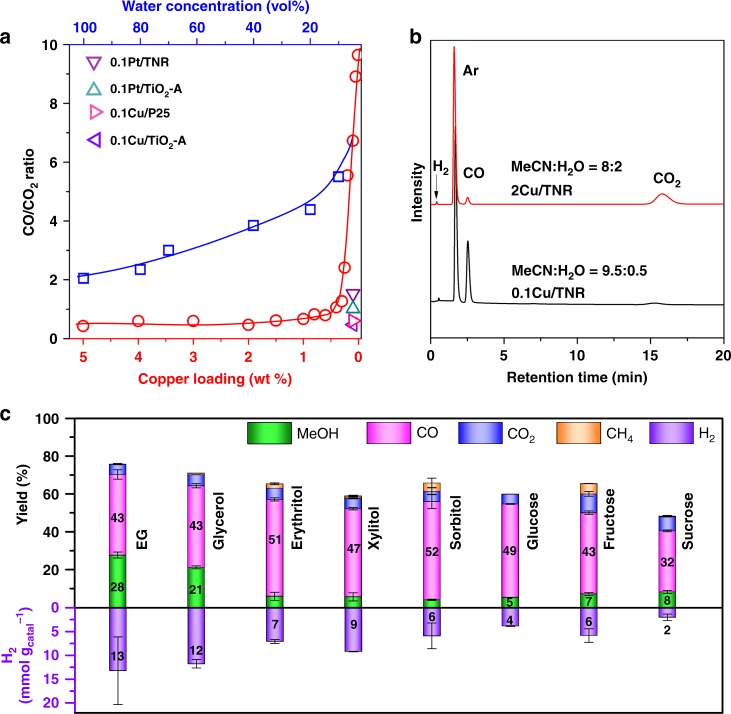
Conversion of biomass resources to syngas. **a** The effect of water content and copper loading on the ratio of CO/CO_2_. Reaction conditions: 10 mg of glycerol, 10 mg of catalyst, 365 nm LED (18 W, 55 mW cm^−2^) irradiation for 12 h. For the investigation of water concentration effect, 0.1Cu/TNR was used as a catalyst. For the investigation of copper loading effect, 0.95 mL of MeCN and 0.05 mL of water was used as solvent. **b** GC curves of the gas products. **c** Photo-reforming of different polyols. Error bar represents the standard deviation. Detailed reaction conditions and product analysis are presented in Supplementary Table 4.

Although methanol was obtained from various biomass resources, a large amount of CO_2_ was also produced along with H_2_ in the gas phase. If CO is generated instead of CO_2_, a syngas will be produced. Syngas can be further converted into methanol^11,12,25–28^. We further attempted to improve the selectivity of CO. In order to prevent the formation of CO_2_ and increase the selectivity of CO, we optimized the reaction conditions and the structure of Cu/TNR. The water concentration and copper loading were two key factors that influence the CO/CO_2_ ratio (Fig. 3a). CO/CO_2_ ratio increases with a decrease in water concentration. The CO_2_ comes from the decarboxylation of carboxylic acids which are generated via reaction with HO∙ radicals^7^. We have detected the hydroxyl radical using electron paramagnetic resonance (EPR), and 5,5-dimethyl-1-pyrroline N-oxide (DMPO) was used to capture the hydroxyl radicals (Supplementary Fig. 5). In water solvent, the signals of hydroxyl radicals were detected. When decreasing the water content to 5%, no obvious signals of hydroxyl radicals were observed. It can be deduced that decreasing the concentration of water reduces the hydroxyl radicals and thus slows down the degradation of organic species to CO_2_.

The copper loading amount shows a more remarkable effect on the CO/CO_2_ ratio. The CO/CO_2_ ratio slowly increases from 0.5 to 1 when the copper loading is reduced from 5 wt% to 0.5 wt%. Further decreasing the copper loading below 0.5 wt% significantly favors the formation of CO. The CO/CO_2_ ratio sharply increases from 1 to 9.6 when the copper loading is decreased from 0.5 wt% to 0.01 wt%. With 0.1Cu/TNR as a catalyst, CO was favorably produced even in pure water with a CO/CO_2_ ratio of 2. At the same loading amount of metal, 0.1Pt/TNR, 0.1Pt/TiO_2_-A, 0.1Cu/TiO_2_-A, and 0.1Cu/P25 all gave a low CO/CO_2_ ratio (0.5–1). As the reaction proceeded, the yields of CO and CO_2_ gradually increased, while the yield of methanol first increased and then decreased (Supplementary Fig. 6). This indicates that methanol can be further decomposed to CO and H_2_. Under optimized conditions, 27% yield of methanol and 45% yield of CO were obtained along with H_2_/CO ratio of 0.8 and 96% conversion of glycerol. We further investigated other biomass resources, such as sugars and sugar alcohols. Ethylene glycol (EG) was converted into methanol (28%), CO (43%), and CO_2_ (5%). Compared with glycerol and EG, sugars and sugar alcohols possess longer carbon chains and require a longer reaction time to achieve a high CO yield. Sugar alcohols, including erythritol, xylitol, and sorbitol, are converted into 6–10% yield of MeOH, 46–52% yield of CO, 5–6% yield of CO_2_ and 1–5% CH_4_ along with 5–10 mmol g_cat._^−1^ of H_2_. Monosaccharides, such as fructose and glucose, are facially converted into CO and methanol. With 0.1 Cu/TNR as a catalyst, 5% yield of MeOH and 49% yield of CO was obtained from glucose, and 7% yield of MeOH and 43% yield of CO was produced if fructose was used as the feed. Sucrose, which consists of one glucose and fructose, is relatively inert and was converted into 8% yield of MeOH and 32% yield of CO.

### Conversion of cellulose and beech sawdust

**Fig. 4:**
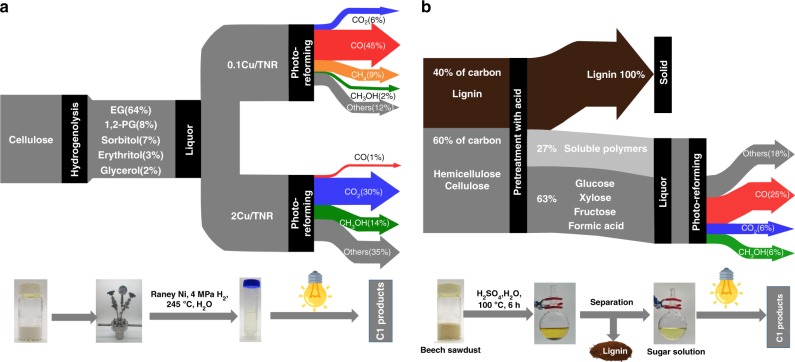
Two-step strategy for the conversion of cellulose and sawdust into methanol and syngas. **a** Cellulose **b** beech sawdust. Detailed reaction conditions and product analysis are presented in Supplementary Tables 5, 6.

Cellulose is the most abundant biomass resources and rich in diol structure, which is a promising candidate for the synthesis of methanol and syngas. Nevertheless, the insolubility and polymeric properties of the cellulose make it inert to be directly converted using the present method. We then tried to convert cellulose via a two-step strategy (Fig. 4a). In the first step, cellulose was hydrogenolyzed to a mixture of soluble polyols. Then, the polyols mixture was subjected to a photo-reforming reaction. Using 0.1Cu/TNR as a catalyst, 45% yield of CO, 6% yield of CO_2_ and 9% yield of CH_4_ along with 9.5 mmol g_cat._^−1^ of H_2_ in the gas phase were obtained. With 2Cu/TNR as a catalyst, 14% yield of methanol was produced together with 30% yield of CO_2_ and 1% yield of CO in the gas phase.

Finally, we tried to use the raw sawdust as the substrate via a two-step strategy **(**Fig. 4) Beech sawdust was firstly treated in an acidic solution. The solid lignin which contains 40% of carbon of beech sawdust was removed, generating a sugar solution which contains 31% of glucose, 20% of xylose, 8% of fructose and 4% of formic acid based on the total carbon of (hemi)cellulose. After photoreaction over 0.1Cu/TNR, 6% yield of methanol, 6% yield of CO_2_ and 25% yield of CO were produced accompanied by the generation of H_2_. Overall, 1 g of beech sawdust will produce 50 mg of methanol in the liquid phase and 212 mL of gas consisting of 140 mL of CO, 36 mL of CO_2_, and 36 mL of H_2_, together with 240 mg of lignin solid.

### The reaction mechanism study

We then investigated the reaction route via the analysis of the products. 1,3-Propanediol, hydroxypropanone, ethylene glycol, oxalic acid, glycolic acid, formaldehyde, formic acid, and 1,2-propanediol, were detected in the reaction of glycerol (Supplementary Fig. 7). The observed products were separately employed as reactants (Fig. 2d). No methanol was generated from 1,3-propanediol and hydroxypropanone. Although glyceraldehyde and glycerol acid were not detected, they are possibly formed via the oxidation of the primary hydroxyl group. A minor amount of methanol was formed with glyceraldehyde and glycerol acid as an initial substrate, indicating that they are not intermediate on rout to methanol. EG is a possible intermediate on rout to methanol as it was detected in the reaction and could be converted to methanol with a high yield. Glycolic acid and oxalic acid were majorly converted to CO_2_. Formaldehyde and formic acid were decomposed to a mixture of CO, CO_2_ and H_2_ under the reaction conditions.

**Fig. 5:**
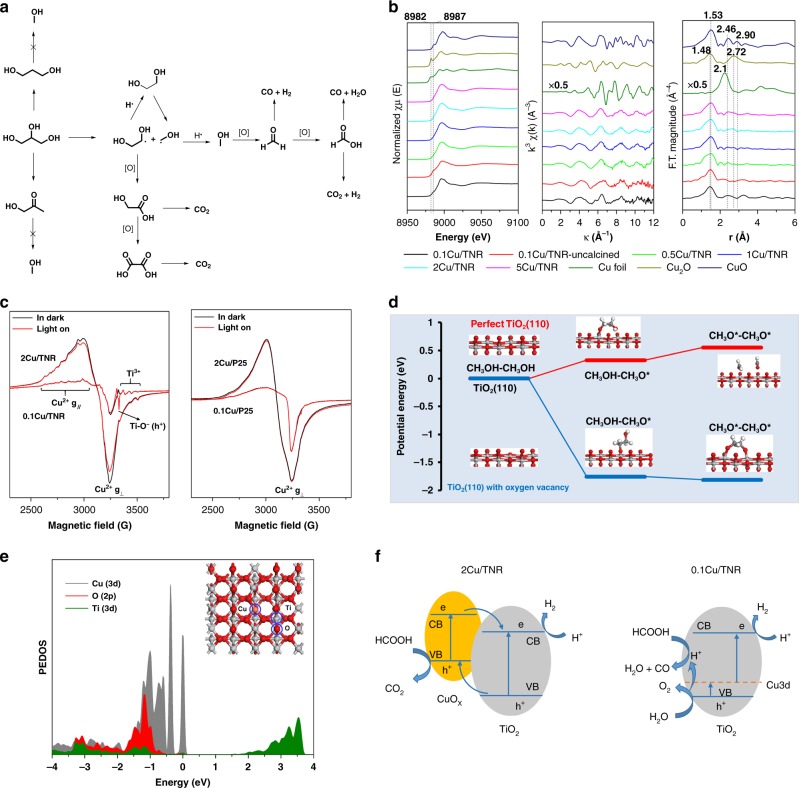
Catalyst characterization and reaction mechanism study. **a** Reaction route for the conversion of glycerol to methanol and syngas. **b** XAFs characterization of copper samples. **c** EPR spectra. **d** DFT calculation of the EG adsorption on the TiO_2_(110) surface. **e** PEDOS of copper doped TiO_2_. **f** The proposed model for the photodecomposition of formic acid over 2Cu/TNR and 0.1Cu/TNR.

Based on the above results, a possible reaction route for the photo-reforming of glycerol to methanol was proposed (Fig. 5a). Upon photoirridiation, the adsorbed glycerol undergoes C–C bond cleavage, forming hydroxymethyl radical and EG radical which were in situ captured by the addition of styrene (Supplementary Fig. 8). The coupling of EG and hydroxymethyl radicals with hydrogen atoms leads to the formation of EG and methanol, respectively. EG further undergoes C–C bond cleavage to hydroxymethyl radicals. The further oxidation of intermediates by HO∙ radicals results in the formation of overoxidation products, such as glycolic acid and oxalic acid. The decarboxylation glycolic acid produces methanol and CO_2_. Oxalic acid is finally decomposed to CO_2_ and H_2_. Oxidation of hydroxymethyl carbon radicals results in the formation of formaldehyde and formic acid, which could be further decomposed to CO, CO_2_, and H_2_.

The Cu/TNR shows higher activity than other TiO_2_ supported copper catalysts in the C–C bond cleavage of polyols to methanol. We performed electron paramagnetic resonance (EPR) characterization (Fig. 5c) and found that Cu/TNR is full of oxygen vacancies as evidenced by the appearance of Ti^3+^ species^29,30^, while Ti^3+^ species is not obvious for Cu/P25. The oxygen vacancy plays a key role in the adsorption and activation of polyols according to DFT calculations (Fig. 5d). The adsorption and activation of EG on the TiO_2_(110) surface with or without oxygen vacancy were considered. Compared with perfect TiO_2_(110) surface, TiO_2_ with the oxygen vacancy is more thermodynamically favored for activation of substrate via dissociative adsorption. EG dissociative adsorption on TiO_2_(110) surface is exothermic (−1.75 eV) while endothermic on the perfect TiO_2_(110) surface (0.33 eV).

The gas product distribution is dominantly controlled by the decomposition way of formic acid. There are two pathways for the decomposition of formic acid. The dehydrogenation of formic acid produces CO_2_ and the dehydration of formic acid generates CO (Fig. 5a). We further investigated copper loading effect of Cu/TNR catalysts in the decomposition of formic acid. The similar trend that with glycerol as substrate was observed and the CO/CO_2_ ratio decreased with the increase of the copper loading (Supplementary Fig. 9), indicating that Cu/TNR with low copper loading favors the dehydration of formic acid and Cu/TNR with high copper loading favors the dehydrogenation of formic acid.

The CO could also be produced from photoreduction of CO_2_ over Cu/TNR. We further used ^13^C labeling experiments to study the source of CO. The photoreforming of glycerol was carried out in the presence of ^13^CO_2_. After reaction, the gas phase was analyzed by mass spectroscopy. Only a small peak of ^13^CO was appeared (Supplementary Fig. 10). The results demonstrated that the CO can be both derived from the decomposition of the intermediates (such as formic acid) in the liquid phase and the reduction of CO_2_ in the gas phase, and the former is the dominant way for the generation of CO from glycerol.

We further performed characterizations to understand the origin of the significant loading effect of copper on the photo decomposition of formic acid to form CO or CO_2_. No copper oxide phase was detected by X-ray diffraction (XRD) for Cu/TNR samples (Supplementary Fig. 11). X-ray of photoelectron spectroscopy (XPS) only detected Cu^+^ species with its Cu 2*p*_3/2_ binding energy at approximately 932.4 eV (Supplementary Fig. 12), which is supposed to be formed via reduction of Cu^2+^ by Ti^3+^ during the calcination process^31^. Cu^2+^ species in Cu/TNR samples were detected by EPR (Fig. 5c). The Cu K-edge X-ray absorption near edge structure (XANES) spectra of and the corresponding differential spectra (Fig. 5b, Supplementary Discussion) confirm the presence of both Cu^2+^ dopants and CuO_x_ particles. In the pre-edge of XANES spectra, 0.1Cu/TNR displays a characteristic Cu^2+^ peak at 8987 eV. In r space, all the samples show a strong Cu–O peak but very weak Cu–Cu peaks, indicating the Cu^2+^ is highly dispersed in the TiO_2_. As the copper loading amount increases, weak Cu–Cu peaks at 2.57 Å (CuO) and 2.60 Å (Cu_2_O) gradually become more obvious, indicating the CuO_x_ clusters or nanoparticles are formed. Copper oxide nanoparticles are observed by transmission electron microscope (TEM) with copper loading above 2 wt% (Supplementary Fig. 13). These results suggest that CuO_x_ particles and Cu^2+^ dopants coexist in the Cu/TNR, forming CuO_x_–TiO_2_ heterojunction and Cu^2+^ defect level (Fig. 5e). With increasing the copper loading amount, the dominant copper species are gradually transformed from the single copper dopants to CuO_x_ particles. Therefore, CuO_x_–TiO_2_ heterojunction dominates in high copper loading and Cu^2+^ defect level prevails in low copper loading (Supplementary Fig. 14).

It is reported that the dehydrogenation of formic acid is more thermodynamically favorable than the dehydration reaction. Under thermal conditions (100 °C), TiO_2_, CuO, and Cu_2_O are all inactive in the decomposition of formic acid. Under light irradiation, the dehydrogenation reaction takes place over CuO and Cu_2_O and generates 100% selectivity of CO_2_, while the dehydration reaction is preferable over TiO_2_, affording 68% selectivity of CO and 32% selectivity of CO_2_ (Supplementary Fig. 15). The results are in accordance with the thermal decomposition of the formic acid demonstrated by the calculations and surface science experiments: copper phase prefers to follow dehydrogenation reaction to afford CO_2_, while TiO_2_ phase favors the dehydration reaction to generate CO^32–34^. We conducted the FTIR characterization of formic acid adsorption on Cu/TNR samples (Supplementary Fig. 16). With low copper loading, the spectra of formic acid adsorption on 0.1Cu/TNR is similar to the formic acid adsorption on TiO_2_^35^. The bands at 1360 and 1581 cm^−1^ are assigned to –COO– symmetric and antisymmetric stretches, 1378 cm^−1^ is signed to C–H deformation. With increasing the copper loading, the bands changed. Besides the shifting of the –COO– symmetric and antisymmetric stretches, new bands at 1650 and 1609 cm^−1^ appeared which are probably due to the formic acid adsorption on CuO_X_. These results clearly explain the copper loading amount effect on the CO/CO_2_ ratio (Fig. 5f). For the Cu/TNR with high copper loading amount (2 Cu/TNR), CuO_x_ nanoparticles are dominant and a CuO_x_–TiO_2_ heterojunction is formed. Upon photoexcitation, the photoexcited holes in the valance band (VB) of TiO_2_ transfer to VB of CuO_x_. Therefore, the oxidation of formic acid by holes takes place on the CuO_x_ phase, and formic acid is decomposed to CO_2_ and H_2_ via dehydrogenation reaction. For the Cu/TNR with low copper loading amount (0.1Cu/TNR), Cu^2+^ single site is doped into the lattice of TiO_2_, forming a defect level in the bandgap. The photoexcited holes remain in the TiO_2_, which is further confirmed by EPR characterization. Upon photoirradiation, the typical holes (Ti–O^−^) signal appears in 0.1 Cu/TNR^36^, but not in 2 Cu/TNR (Fig. 5c). It is reported that the oxidation of water by holes on TiO_2_ generates acid, which is permanent under photoirradiation and distributed close to the surface^37^. Because there is no CuO_x_ phase in 0.1Cu/TNR, the formic acid dominantly adsorbed on TiO_2_ phase. For 0.1Cu/TNR, the holes on TiO_2_ oxidize water to in situ generate acidic TiO_2_ surface under photoirradiation, which may promote the dehydration of formic acid to CO and H_2_O.

## Discussion

We provided a mild way for the conversion of polyols and sugars into methanol and syngas via photo splitting. Cu dispersed on titanium oxide nanorod (Cu/TNR) is effective for the conversion of biomass into syngas and methanol under UV light irradiation at room temperature. The TNR is rich in oxygen vacancy and favors for the activation of polyols via dissociative adsorption. The water concentration and copper loading are two factors that influence the CO/CO_2_ ratio. Water favors for the overoxidation of organic species to carboxylic acids which decompose to CO_2_ via decarboxylation. Decreasing water content suppresses the formation of CO_2_. The copper loading amount controls the decomposition way of the formic acid intermediate. For the Cu/TNR with high copper loading amount, a CuO_x_–TiO_2_ heterojunction is formed and the oxidation of formic acid by holes on the CuO_x_ phase generates to CO_2_ and H_2_. For the Cu/TNR with low copper loading amount, Cu^2+^ dopants are dominant. Formic acid undergoes dehydration on TiO_2_ surface to form CO. A series of biomass resources, including polyols and sugars, could be converted into syngas and methanol. Because the methanol and syngas are feedstocks of the current petrochemical and chemical industries, the present biomass to methanol/syngas process is promising for the utilization of biomass to produce useful commodities for modern society.

## Methods

### Preparation of Cu/TNR

First, protonated titanate nanotubes (H-TNTs) were prepared using the alkaline hydrothermal synthesis. Briefly, 3.0 g of TiO_2_ powder (anatase TiO_2_, Macklin reagent, purchased from Beijing Puyihua Science and Technology CO., LTD) were dispersed into 90 mL of 10 M NaOH aqueous solution under stirring. After stirring for 24 h, the alkaline suspension was transferred into an autoclave with Teflon inner and statically heated at 150 °C for 48 h. The H-TNTs were recovered by washing with 0.1 M dilute HNO_3_ solution and deionized water till neutral. Then, NH_4_ exchanged TiO_2_ nanotubes (NH_4_-TNTs) were prepared from H-TNTs using NH_4_Cl as the N-sources. Briefly, 18 g of NH_4_Cl was added in a 100 mL ethanol-water solution (1:1 volume ratio). The as-recovered H-TNTs were then well dispersed in the NH_4_Cl solution prior to heating reflux at 120 °C for 12 h. After cooling to room temperature, the resulting white precipitates were filtrated and washed with deionized water and ethanol. The obtained NH_4_-TNTs were dried at 80 °C overnight prior to calcination at 400 °C for 2 h. Finally, Cu^2+^-exchanged hydrogen titanate was prepared by an ion-exchange reaction of NH_4_-TNTs in Cu(NO_3_)_2_ aqueous solution. Typically, 0.5 g of NH_4_-TNTs was dispersed in 20 mL of deionized water, into which a certain volume of Cu(NO_3_)_2_ aqueous solutions (0.1 mol·L^−1^) was added. The mixture was stirred for 24 h at room temperature and then heated at 100 °C overnight to remove water. The acquired powders were put in a quartz tube and calcined at 450 °C for 2 h in pipe furnace with a heating rate of 2 °C min^−1^ and 25 mL min^−1^ flow of air. The samples with calculated Cu weight percentages of 0.05, 0.2, 0.5, 1, 2, and 5% were prepared and denoted as XCu/TNR, where X% denotes the Cu weight percentages.

### Reaction procedure and product analysis

The reaction was carried out in home-made LED photoreactors. Typically, 10 mg of substrate and 10 mg of catalyst were added into 1 mL of solvent in a 6.5 mL of quartz tube reactor, then the system was completely replaced with Ar before sealed with a cap. This quartz tube reactor could stand up 0.5 MPa pressure. The quartz tube was then irradiated with 365 nm LED light (18 W, 55 mW cm^−2^) via side irradiation. The reaction temperature was kept between 25 and 35 °C. After the reaction, gas-phase products were analyzed by mass spectroscopy (MS) and gas chromatography (GC) equipped with a TCD detector and TDX-01 column. The gas was injected into the mass spectroscopy and GC via an injector. The liquid phased was analyzed by the GC equipped with FID detector and GDX-02 column, and high performance liquid chromatography (HPLC) equipped with a hydrogen column (Hi-Piex H, 300 × 7.7 mm). Propanol was added into the reaction liquid as the internal standard. The catalyst was filtered, and the supernatant was used for the GC and HPLC analysis.

## Supplementary information

Supplementary Information

Peer Review File

## Data Availability

All data generated and analyzed during this study are included in this Article and its Supplementary Information or are available from the corresponding author upon reasonable request.
