# Supplementary material for: Photo splitting of bio-polyols and sugars to methanol and syngas
Source: Nat Commun. 2020 Feb 27;11:1083. doi: 10.1038/s41467-020-14915-8 (PMC7046649; doi:10.1038/s41467-020-14915-8)
Supplement: Supplementary file 1 — Supplementary Information [file 41467_2020_14915_MOESM1_ESM.pdf]

Supplementary Information for

Photo splitting of bio-polyols and sugars to methanol and syngas

Wang et al.

Supplementary Materials

All commercial chemicals were analytical reagents and were used without further purification. P25, anatase TiO_2, Nb_2O_5, CuO, ZnO, WO_3, Nb_2O_5 were purchased from Alfa Aesar. NaOH, NH_4Cl, HNO_3, $Cd(NO_3)_2$, $Cu(NO_3)_2$, H_2PtCl_4, $Fe(NO_3)_2$ and $Ni(NO_3)_2$ NH_4VO_3 (3 mmol) and $Bi(NO_3)_3$ $Na_2WO_4{\cdot}2H_2O$ were purchased from Alfa Aesar. Glycerol and EG were purchased from Alfa Aesar. Acetonitrile, dioxane, DMF, DMSO, glucose, fructose, sucrose, and cellulose were purchased from Alfa Aesar. Water was purified using a Millipore Milli-Q Advantage A10 water purification system to a resistivity higher than 18 MΩ.cm.

Supplementary Methods

Preparation of Cu/TNR

Step 1: preparation of protonated titanate nanotubes (H-TNTs)

The H-TNTs were prepared using the alkaline hydrothermal synthesis. Briefly, 3.0 g of TiO_2 powder (anatase TiO_2, Macklin reagent, purchased from Beijing Puyihua Science and Technology CO., LTD) were dispersed into 90 mL of 10 M NaOH aqueous solution under stirring. After stirring for 24 h, the alkaline suspension was transferred into an autoclave with Teflon inner and statically heated at 150 ^oC for 48 h. The H-TNTs were recovered by washing with 0.1 M dilute HNO_3 solution and deionized water till neutral.

Step 2: preparation of NH_4 exchanged TiO_2 nanotubes (NH_4-TNTs)

The N H_4-TNTs were prepared using NH_4Cl as the N-sources. [1] Briefly, 18 g of NH_4Cl was added in a 100 mL ethanol-water solution (1:1 volume ratio). The as-recovered H-TNTs were then well dispersed in the NH_4Cl solution prior to heating reflux at 120 ^oC for 12 h. After cooling to room temperature, the resulting white precipitates were filtrated and washed with deionized water and ethanol. The obtained materials were dried at 80 ^oC overnight prior to calcination at 400 °C for 2 h.

Step 3: preparation of Cu/TNR

Cu^{2+}-exchanged hydrogen titanate was prepared by an ion-exchange reaction of NH_4-TNTs in $Cu(NO_3)_2$ aqueous solution. Typically, 0.5 g of NH_4-TNTs was dispersed in 20 mL of deionized water, into which a certain volume of $Cu(NO_3)_2$ aqueous solutions (0.1 mol·L^{-1}) was added. The mixture was stirred for 24 h at room temperature and then heated at 100 ^oC overnight to remove water. The acquired powders were put in a quartz tube and calcined at 450 ^oC for 2 h in pipe furnace with a heating rate of 2 ^oC min^{-1} and 25 mL min^{-1} flow of air. The samples with calculated Cu weight percentages of 0.05%, 0.2%, 0.5%, 1%, 2% and 5% were prepared and denoted as XCu/TNR, where X% denotes the Cu weight percentages.

Preparation of N-TNTs

The N-TNTs were prepared by calcination of NH_4-TNTs at 400 ^oC for 2 h. Typically, 0.5 g of NH_4-TNTs was put in a quartz tube and calcined at 400 ^oC for 2 h in pipe furnace with a heating rate of 2 ^oC min^{-1} and 25 mL min^{-1} flow of air.

Preparation of supported copper catalyst

The supported copper catalyst with 1 wt % metal loading was prepared by the impregnation method. Typically, 0.5 g of support was dispersed in 20 mL of deionized water, into which a certain volume of $Cu(NO_3)_2$ aqueous solutions (0.1 mol·L^{-1}) was added. The mixture was stirred for 24 h at room temperature and then heated at 100 ˚C overnight to remove water. The acquired powders were put in a quartz tube and calcined at 450 ^oC for 2 h in pipe furnace with a heating rate of 2 ^oC min^{-1} and 25 mL min^{-1} flow of air.

Preparation of $BiVO_4$

$BiVO_4$ was prepared by a hydrothermal method as previously reported.[2] Typically, 3 mmol of NH_4VO_3 and 3 mmol of $Bi(NO_3)_3{\cdot}5H_2O$ was dissolved in 25 mL of 2.0 M nitric acid solutions, and the pH value of the solution was then adjusted to 2.0 with ammonia solution under stirring. After aging for 2 hours, the orange precipitate at the bottom of the beaker was transferred to a 50 mL Teflon-lined stainless steel autoclave and hydrothermally treated at 200 ^oC for 24 h. After the autoclave was cooled to room temperature, the yellow powder was separated by filtration, washed with de-ionized water for more than 3 times, and then dried at 70 ^oC for overnight.

Preparation of Bi_2WO_3

Bi_2WO_6 was synthesized according to the literature report.[3] The general procedures are as follows. Firstly, 2 mmol of $Bi(NO_3)_3{\cdot}5H_2O$ was dissolved in 15 mL of 1.0 M HNO_3 aqueous solution, and 1 mmol of $Na_2WO_4{\cdot}2H_2O$ was dissolved in 15 mL of H_2O. Then, the Na_2WO_4 solution was added into the $Bi(NO_3)_3$ solution and stirred for 30 min, after which the mixture was transferred to a 50 mL autoclave and heated at 180 ^oC for 24 h in an oven. After the hydrothermal crystallization process, the mixture was filtered, washed with deionized water several times and dried at 100 ^oC for 12 h, giving the final solid Bi_2WO_6 sample.

Preparation of BiOCl

BiOCl was synthesized according to the literature report.[4] In a typical synthesis, 0.486 g of $Bi(NO_3)_3{\cdot}5H_2O$ and 0.400 g of PVP were dissolved in 25 mL of 0.1 M mannitol solution. Then, 5 mL of saturated NaCl solution was slowly added into the above mixture. After magnetically stirring for 10 min, the mixture was transferred into a Teflon-lined stainless steel autoclave and heated at 160 °C for 3 h in an oven. Then, the autoclave was cooled to room temperature. The solid powder was collected by centrifugation and washed with deionized water three times. The final solids were then dried at 60 ^oC for 4 h.

Preparation of CdS

CdS was synthesized by a hydrothermal method reported previously.[5] A Na_2S solution (20 mL, 0.2 M) was added dropwise into the $Cd(NO_3)_2$ (40 mL, 0.0875 M) under continuous stirring. After further stirred at room temperature for 0.5 h, the mixture was transferred into a Teflon-lined stainless steel autoclave and was subject to hydrothermal treatment at 180 ^oC for 12 h. Then, the solid was collected by centrifugation and washing with deionized water for five times and then drying at 80 ^oC for 12 h.

Preparation of $ZnIn_2S_4$/P25

$ZnIn_2S_4$/P25 composites were prepared as follows: 0.2 g of P25, 0.075 g of $Zn(NO_3)_2{\cdot}6H_2O$, 0.019 g of $In(NO_3)_3{\cdot}H_2O$, 0.015 g of thioacetamide were added into 10 mL of water. The mixture was heated under 100 ^oC for 24 h. Then, the solid was collected by centrifugation and washing with deionized water for five times and then drying at 60 ^oC for 12 h.

Preparation of Cu/$ZnIn_2S_4$

$ZnIn_2S_4$/P25 composites were prepared as following: 0.1 mmol $Cu(NO_3)_2{\cdot}3H_2O$, 0.9 mmol of $Zn(NO_3)_2{\cdot}6H_2O$, 2 mmol $InCl_3{\cdot}4H_2O$, 8 mmol of thioacetamide and 0.7 mmol hexadecyl trimethyl ammonium Bromide were added into 20 mL of deionized water. The mixture was added into an autoclave and heated under 160 ^oC for 24 h. Then, the solid was collected by centrifugation and washing with deionized water and ethanol for five times and then drying at 60 ^oC for 12 h.

Preparation Pt/P25

Pt/P25 with 1 wt % weight of Pt was prepared by the impregnation method. Typically, 0.5 g of support was dispersed in 20 mL of deionized water, into which a certain volume of H_2PtCl_4 aqueous solutions (0.1 mol·L^{-1}) was added. The mixture was stirred for 24 h at room temperature and then heated at 100 ^oC overnight to remove water. The acquired powders were put in a quartz

tube and reduced at 300 ^oC for 2 h in pipe furnace with a heating rate of 2 ^oC min^{-1} and 25 mL min^{-1} flow of H_2.

Preparation M/P25 (M=Fe，Co，Ni)

The P25 supported catalyst with 1 wt % weight of metal was prepared by the impregnation method. Typically, 0.5 g of P25 was dispersed in 20 mL of deionized water, into which a certain volume of $M(NO_3)_2$ aqueous solutions (0.1 mol·L^{-1}) was added. The mixture was stirred for 24 h at room temperature and then heated at 100 ^oC overnight to remove water. The acquired powders were put in a quartz tube and calcined at 450 ^oC for 2 h in pipe furnace with a heating rate of 2 ^oC min^{-1} and 25 mL min^{-1} flow of air.

Characterizations

Fourier transform infrared (FT-IR) spectra were collected on a Bruker Tensor 27 FT -IR spectrometer. About 30 mg of Cu/TNR samples was pressed into a plate. After collecting the background of Cu/TNR, 10 μl of formic solution was dropped into the plate which is dried in vacuum at 60 ^oC for 10 min. Then, the adsorption spectra was collected via subtraction of the Cu/TNR background. The X-ray powder diffraction (XRD) patterns were obtained using a Rigaku D/Max 2500/PC powder diffractometer with Cu Kα radiation (λ = 0.15418 nm). High resolution transmission electron microscopy (HRTEM) was performed using JEOL JEM-2100 electron microscope operated at 220 kV. The X-ray photoelectron spectroscopy (XPS) measurements were performed on a Thermo ESCALAB 250Xi spectrometer equipped with a monochromated AlKα X-ray source (hυ = 1486.6 eV, 15 kV, 10.8 mA). The samples were dried in vacuum at 120 °C for 12 h. The charge neutralizer system was used for all of the analyses. The base pressure was 1×10^{-8} Pa. High resolution spectra were recorded with 20 eV pass energy. The pass energies correspond to the Ag3$d_{5/2}$ FWHM of 0.65 eV. The data was acquired with 0.05 eV steps. The binding energy (BE) was calibrated to the C1*s* signal (284.6 eV) as a reference. The curve fitting procedure was performed using an approximation based on a combination of the Gaussian and Lorentzian functions with the subtraction of a Shirley-type background. The X-ray absorption fine structure (XAFS) experiment was performed at the bending magnet beamline BL12B of SPring-8 (8 GeV, 100 mA) belong to National Synchotron Radiation Research Center, in which the X-ray beam was monochromatized with water-cooled Si (111) double-crystal monochromator and focused with two Rh coated focusing mirrors with the beam size of 2.0 mm in the horizontal direction and 0.5 mm in the vertical direction around sample position. The samples were filled in a phi10 aluminium tube sealed with graphite tape in Ar gas protected glovebox for quasi in situ XAFS measurements. All of the samples were measured by both transmission and fluorescence modes at Cu K-edge. The spectra were analyzed and fitted using an analysis program Demeter(Ravel, B. & Newville, M. ATHENA, ARTEMIS, HEPHAESTUS: data analysis for X-ray absorption spectroscopy using IFEFFIT. J. Synchrotron Radiat 12, 537-541 (2005).). The electron paramagnetic resonance (EPR) were performed on Bruker spectrometer at X-band under room temperature, with a field modulation of 100 kHz. The microwave frequency was kept at 9.401 GHz. For in situ ESR measurements, 2Cu/TNR were dispersed in a mixed solution of 95 vol% MeCN and 0.5 vol% H_2O containing DMPO (0.1 M), which was used as a spin-trapping agent, by ultrasonic treatment. Then, the suspension was injected into a glass capillary and the glass capillary was placed in a sealed glass tube under Ar atmosphere. The sealed glass tube was placed in the microwave cavity of EPR spectrometer and was irradiated with Hg lamp during EPR measurements at room temperature.

DFT calculations

All of the first-principles electronic structure calculations were carried out using the Vienna ab initio simulation package (VASP), [6] one density functional theory implementation. The exchange correlation potential was described by the Perdew–Burke–Ernzerhof (PBE) [7] formulation of the generalized gradient approximation (GGA). The ion–electron interactions were represented by the projector augmented wave (PAW) [8] method, while the valence electrons ($2s^2 2p^4$ of O, $3s^2 3p^6 3d^2 4s^2$ of Ti, and $3d^{10} 4s$ of Cu) were expanded by a plane wave basis set with an energy cutoff of 400 eV. The *k*-point sampling was performed using the Monkhorst–Pack scheme. [9] The electronic self-consistent minimization was converged to 10^{-5} eV, and the geometry optimization was converged to 10^{-4} eV. The self-interaction error (SIE) was mitigated using the DFT+U method by Dudarev and his colleagues.[10] A typical *U* value of 4.5 eV was used for Ti.

The lattice constants of anatase TiO_2 were optimized to be a = 3.855 Å and c = 9.661 Å, in good agreement with the experimental constants, a = 3.782 Å and c = 9.502 Å.[11] We used them to build a $p(2 \times 4)$ TiO_2(101) slab with 12 atomic layers and a vacuum of 15 Å. Atoms in the bottom 6 atomic layers were fixed to their bulk positions, while the rest were allowed to fully relax. A 4 × 3 × 1 *k*-point mesh was used.

Reaction procedure and product analysis

The reaction was carried out in home-made LED photoreactors. Typically, 10 mg of substrate and 10 mg of catalyst were added into 1 mL of solvent in a 6.5 mL of quartz tube reactor, then the system was completely replaced with Ar before sealed with a cap. This quartz tube reactor could stand up 0.5 MPa pressure. The quartz tube was then irradiated with 365 nm LED light (18 W, 55 Mw cm^{-2}) via side irradiation. The reaction temperature was kept between 25-35 ^oC. After the reaction, gas-phase products were analyzed by mass spectroscopy (MS) and gas chromatography (GC) equipped with a TCD detector and TDX-01 column. The gas was injected into the mass spectroscopy and GC via an injector. The liquid phased was analyzed by the GC equipped with FID detector and GDX-02 column, and high performance liquid chromatography (HPLC) equipped with H column. Propanol was added into the reaction liquid as the internal standard. The catalyst was filtered and the supernatant was used for the GC and HPLC analysis.

The H_2 and CO_2 were quantified by mass spectroscopy using an internal standard method. Ar was used as the internal standard. The molecule molecular ion peak area was used for calculation. The differential response was calibrated using the response factor. The detailed calculation was as follows:

$V_{H_2} = A_{H_2}/A_{Ar} \times V_{Ar} \times K_{H_2}$

$V_{CO_2} = \mathrm{A}_{\mathrm{CO_2}}/A_{Ar} \times V_{Ar} \times K_{CO_2}$

$R_{H_2} = V_{H_2}/m_{catalsyt}$

In these equations:

V_{H_2} : the volume of H_2 in the quartz tube reactor;

V_{CO_2}: the volume of CO_2 in the quartz tube reactor;

R_{H_2}: hydrogen generation volume per mg of catalyst;

V_{Ar}: the volume of Ar in the quartz tube reactor (5.5 mL);

A_{H_2} : the peak area of H_2 (m/z=2);

A_{Ar}: the peak area of Ar (m/z=40);

A_{CO_2} : the peak area of H_2 (m/z=44);

K_{H_2} : the H_2 response factor (0.1) related to Ar;

K_{CO_2} : the CO_2 response factor (2.65) related to Ar.

The CO and CH_4 were quantified by GC equipped with FID detector and TDX-01 column. He was used as the carrier gas. CO_2, quantified by MS, was used as an internal standard to calculate the amount of CO and CH_4. The detailed calculation was as follows:

$V_{CH_4} = A_{CH_4}/A_{CO_2}\times V_{CO_2}\times K_{CH_4}$

$V_{CO}= A_{CO}/A_{CO_2}\times V_{CO_2}\times K_{CO}$

In these equations:

V_{CH_4} : the volume of CH_4 in the quartz tube reactor;

V_{CO}: the volume of CO in the quartz tube reactor;

A_{CH_4} : the peak area of CH_4;

A_{CO}: the peak area of CO;

A_{CO_2} : the peak area of H_2;

K_{CH_4} : the CH_4 response factor (1.79) related to CO_2;

K_{CO} : the CO response factor (0.8) related to CO_2.

The yields are calculated based on sum of total carbon in the products.

Yp=(NpＸCp)/($N_{substrate}$Ｘ$C_{substrate}$)Ｘ100, where Np is the molar of product, Cp is the number of carbon in the product, $N_{substrate}$ is the molar of substrate, and $C_{substrate}$ is the number of carbon in the substrate.

The molar of gas products is calculated based on the gas equation.

N = PV/RT=101.3ＸV/(8.314ＸT)， where N is the molar of gas, V is the volume of the gas product, T is the temperature.

The volume of gas is depended on the temperature. We calculated the molar of gas product at room temperature (298 K).

Supplementary Discussion

Reaction conditions optimization

The solvent and copper loading amount of Cu/TNR showed a great effect on the photo-reforming of glycerol to methanol. The water-organic solvent mixture solvent was used as the solvent. MeCN, dioxane, DMSO and DMF were used as the organic solvent. MeCN shows the highest yield of methanol among these solvents. The ratio of organic solvent to water affects the reactions. The methanol yield first increases and then decreases with the increase of MeCN concentration. MeCN with 80% volume concentration achieves the highest yield of methanol. Cu/TNR with 0.25-5 wt % copper loading were investigated in the photo-reforming of glycerol. A 1-2 wt % copper loading shows the best performance.

The reaction route study

The liquid phase products were detected by HPLC. HPLC analysis was performed on an Agilent system equipped with a RID-6A refractive index detector. A hydrogen column (Hi-Piex H, 300 Ｘ7.7 mm) was used with pure water as eluent (flow rate of 0.6 ml min^{-1}). The temperature of the column was kept at 65 ºC. Ten microlitres of the sample were injected. Oxalic acid, glycolic acid, formaldehyde, formic acid, ethylene glycol, hydroxypropanone, 1, 3-propanediol and methanol were detected (Figure S4). The observed products in the reaction of glycerol were separately employed as reactants (Figure 1). Hydroxypropanone and 1, 3-propanediol were probably formed via dehydration of primary and secondary hydroxyl groups, respectively, which could not be converted into methanol under the standard reaction conditions, indicating they were not the intermediates to methanol. EG was a possible intermediate on rout to methanol as it is detected in the reaction and could be converted to methanol in high yield. Glycolic acid and oxalic

acid were the overoxidaiton product of EG and mainly converted into CO_2, indicating they are contributed to the formation of CO_2. Formaldehyde and formic acid were the overoxidation product of methanol and were decomposed to CO, CO_2 and H_2 under the reaction conditions. Based on these results, the reaction route was proposed. Glycerol first undergoes C-C bond cleavage to form EG and methanol. EG can further undergo C-C bond cleavage to form methanol. The dehydration of glycerol generates hydroxypropanone and 1, 3-propanediol byproducts. The overoxidation of EG and methanol leads to the formation of overoxidation products, such as glycolic acid, oxalic acid, formaldehyde and formic acid, which were finally converted to CO, CO_2 and H_2.

The C-C bond cleavage of glycerol may produce EG and hydroxymethyl radicals. We then try to capture the radicals. The carbon-based radical can add to the C=C bond. Styrene was added into the reaction to capture the radicals. After the reaction, the reaction liquid was qualified by GC-MS. 3-Phenylpropanol and 4-phenylbutane-1,2-diol were detected, confirming the formation of EG and methoxyl carbon radicals via C-C bond cleavage.

Conversion of cellulose

It is a challenge to direct convert cellulose due to the poor solubility. We tried to convert cellulose via a two-step strategy. Firstly, the hydrogenolysis of cellulose to a mixture of polys and then photo-reforming of the reaction liquid to methanol or syngas. The reaction liquid was provided from Tao Zhang group (Dalian Institute of Chemical Physics, Chinese Academy of Sciences). The catalytic conversion of cellulose (Merck, microcrystalline) was carried out in a stainless-steel autoclave (Parr Instrument Company, 100 mL) at an H_2 pressure of 4 MPa (measured at room temperature) and at 518 K for 30 min. For each reaction, 0.5 g of cellulose, 0.05g of Raney Ni, 0.05 g of tungstic acid and 50 mL of water were put into the reactor and stirred at a rate of 1000 r min^{-1}. After the reaction, the catalyst was filtered. The water reaction solution was treated with $Ba(OH)_2$ to remove the tungstic acid, and the pH value of the resulting solution is 9-10. Cellulose was completely converted. The yields of polyols were as follows: 6.9% of sorbitol, 3.3% of erythritol, 2.2% of glycerol, 63.6% of EG and 7.7% of 1, 2- propanol.

The procedure for the photo-reforming of the cellulose reaction solution is as follows.

Procedure A: 10 mg of 2Cu/TNR, 0.2 mL (equal to 2 mg of the cellulose) of the reaction solution, 0.8 mL of MeCN, were added into the quartz tube reactor, then the system was completely replaced with Ar before sealed with a cap. The quartz tube was then irradiated with 365 nm LED light (9 W) for 12 h. The reaction temperature was kept between 25-35 ^{o}C.

Procedure B: 10 mg of 0.1Cu/TNR, 0.05 mL (equal to 2 mg of the cellulose) of the concentrated reaction solution, 9.5 mL of MeCN, were added into the quartz tube reactor, then the system was completely replaced with Ar before sealed with a cap. The quartz tube was then irradiated with 365 nm LED light (9 W) for 72 h. The reaction temperature was kept between 25-35 ^{o}C.

Under the procedure A, 16% yield of methanol was obtained based on the cellulose. Under the procedure B, 50% and 5% yield of CO and CO_2 were obtained from cellulose, respectively. The ratio of H_2/CO is 6.7.

Conversion of native biomass

The beech sawdust was treated with an acid solution to convert the hemicellulose and cellulose into soluble sugars. The resulting sugar solution was further used as the substrate for the photo-reforming reaction. Typically, 0.5 g of each biomass sample was loaded into 50-mL beakers with the addition of 7.5 mL of a 72 wt % H_2SO_4 solution. The mixture was left at room temperature for 2 h under stirring. Afterward, the slurry was transferred into a round-bottom-flask and 90 mL of water was added to reach a H_2SO_4 concentration of 3 wt %. The solution was heated under reflux

(100 ºC) under magnetically stirring for 6 h. The resultant solution was filtered and the filtrate was analyzed by HPLC. The as-obtained solution was treated with $Ba(OH)_2$ to removed H_2SO_4. The formed $BaSO_4$ solid was filtered, and the filtrate was concentrated to 10 mL. The pH value of the final solution was 9-10. Then, the as-obtained solution was subjected to further photo reaction. Typically, 10 mg of 0.1Cu/TNR and 50 μl of the solution were added to 0.95 mL of MeCN in a 6.5 mL of quartz tube reactor, then the system was completely replaced with Ar before sealed with a cap. This quartz tube reactor could stand up 0.5 MPa pressure. The quartz tube was then irradiated with 365 nm LED light (18 W). The reaction temperature was kept between 25-35 ºC. The results were shown in table S5.

After acid treatment, based on the mass of the beech sawdust, 21 wt % glucose, 14 wt % xylose, 6 wt % fructose and 4 wt % formic acid were left in the solution, which accounts for 45 wt % of the beech sawdust. The soluble products in the solution come from the (hemi)cellulose part of beech sawdust. Based on the (hemi)cellulose, 88 wt % of (hemi)cellulose was released to the solution as small molecules. The carbon content of beech sawdust was determined to 49 wt % by element analysis. Based on the carbon analysis, about 33% carbon of beech sawdust was left in the solution. After photoreaction for 6 h, based on the carbon in the solution, 11% of methanol, 44% of CO and 11% of CO_2 were formed, which accounts for 66% and 22% of carbon in the solution and beech sawdust, respectively. Further prolonging the reaction time to 24 h generates 50% of CO, 23% of CO_2 and 23% of CH_4, which accounts 96% and 32% of carbon in the solution and beech sawdust, respectively.

Carbon mass balance analysis

The liquid phase products were analyzed by HPLC. After photoreaction, the liquid phase products are mostly the same for different substrates, which is probably due to the similar polyols structure. The identified compounds are as follows: oxalic acid, glycolic acid, formaldehyde, formic acid, ethylene glycol, hydroxypropanone, 1, 2-propanediol, 1, 3-propanediol and methanol. For some substances, there are some unidentified compounds, which accounts for the relatively lower carbon balance. The results were shown in Table S1-5. The carbon balance is calculated based on the following equation.

$$\text{Carbon balance} = \sum M_i \times n_i / M \times n \times 100$$

In these equations:

M_i: the molar of the products;

M: the molar of the substance;

n_i: the carbon number of the products;

n: the carbon number of the substance;

Beech sawdust is derived from the group of Fang Lu (Dalian Institute of Chemical Physics, Chinese Academy of Sciences). The composition has been analyzed,[12] which comprises 47 wt % of cellulose, 21 wt % of hemicellulose and 21 wt % of lignin. The total carbon in beech sawdust and the solid after acid treatment of beech sawdust were 45 wt % and 64 wt %, respectively. The total carbon content of cellulose and hemicellulose was calculated to be 60%. The carbon content of lignin is calculated to be 40%. The carbon balance for the beech sawdust is calculated based on cellulose and hemicellulose.

The apparent quantum yield (AQY) was measured over 2Cu/TNR with UV LEDs light (input power of 50 W, 365 nm) by top irradiation. We measured the apparent quantum yield was calculated based on the following equation.

Apparent quantum yield (AQY) measurements

The AQY was measured over 2Cu/TNR in MeCN-H_2O (8:2) solution with UV LEDs light (input power of 50 W, 365 nm) by top irradiation. We measured the apparent quantum yield was calculated based on the following equation (Supplementary Equations 1).

$$AQY = \frac{electrons\ consumed\ by\ the\ products}{Photons} \times 100$$
$$= \frac{\frac{4}{3}n_{methanol} \times N_A + \frac{1}{3}n_{EG} \times N_A + 2n_{hydrogen} \times N_A}{I \times t \times S} \times 100 \quad \text{Supplementary Equations 1}$$

where $n_{methanol}$, $n_{methanol}$, and $n_{hydrogen}$ are the molar amount of methanol, EG and hydrogen, respectively, and N_A, I, t and S represent Avogadro's constant, light intensity, reaction time and irradiation area, respectively.

Catalyst characterization

To understand the form and structure of the copper species in the Cu/TNR catalysts prepared under different conditions, Cu K-edge XAFS was used for local structure analysis of the Cu species as shown in Figure 5b, along with those of Cu, Cu_2O and CuO. Cu_2O exhibit a low energy peak in the region between 8982 eV, which has been assigned as a Cu l*s*→4*p* transition.[13] The X-ray absorption pre-edge feature of CuO shows a weaker peak at about 8987 eV. In addition, after the absorption maximum, the absorption intensities of Cu and Cu_2O show a little decrease, while that of CuO decreases more sharply. At low copper loading, 0.1Cu/TNR shows a peak at 8987 eV, while with increasing the copper loading, the peak in the pre-edge is not obvious, but it can be estimated that the copper species in them exist mainly as Cu^{2+} because their sharply decreased absorption features are similar to that of CuO.

Figure 5b shows the radial structure functions (RSF) of Cu, Cu_2O, CuO, and Cu/TNR samples obtained by k3-weighted Fourier transformation. According to the literature,[14,15] metallic Cu shows a strong peak at about 2.10Å corresponding to the Cu–Cu distance. Cu_2O shows two strong peaks at about 1.48 and 2.72Å corresponding to the Cu–O and Cu–Cu distances, respectively. CuO shows three distinct peaks at about 1.53Å (Cu–O), 2.46Å (Cu–Cu) and 2.90Å (Cu–Cu). At low copper loading, the presence of Cu–O peak and absence of Cu–Cu peak for 0.1Cu/TNR indicates the Cu^{2+} single sites are highly dispersed in the TiO_2. As the copper loading increase, weak Cu-Cu peaks gradually appeared, indicating the CuO clusters or nanoparticles formed. These results suggest that the copper species gradually transformed from single copper dopant to CuO particles.

The above characterization results demonstrated that there are two kinds of copper species: the doped Cu^{2+} and CuO_x nanoparticles. At low copper loading amount, highly dispersed Cu^{2+} dopants are dominant, and with increasing the copper loading amount, CuO_x nanoparticles gradually became the major copper species.

Supplementary Figures

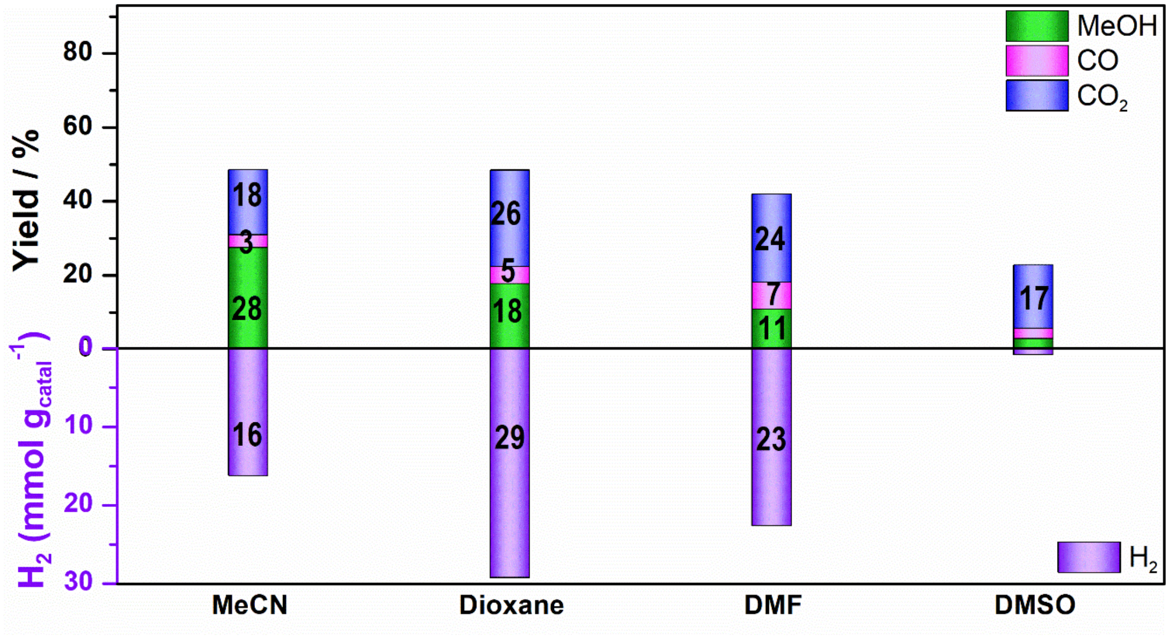

Supplementary Figure 1 The effect of the solvent. Reaction conditions: 10 mg of glycerol, 10 mg of 1Cu/TNR, 0.8 mL of organic solvent, 0.2 mL of water, 365 nm LED (18 W, 55 mW cm^{-2}) irradiation for 12 h.

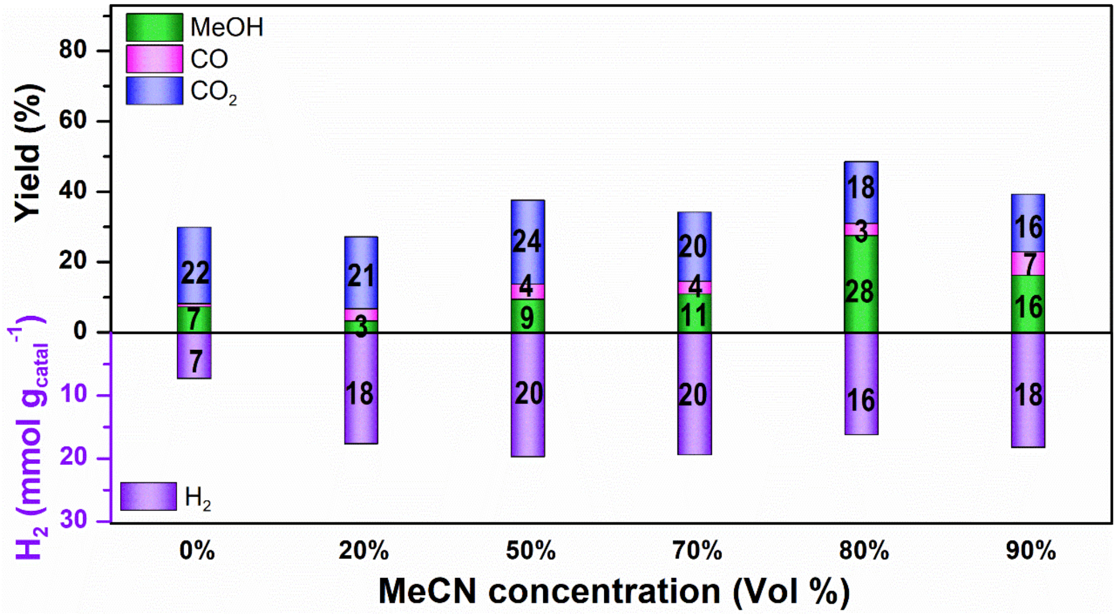

Supplementary Figure 2 The effect of MeCN concentration. Reaction conditions: 10 mg of glycerol, 10 mg of 1Cu/TNR, 1 mL of MeCN-H_2O solvent, 365 nm LED (18 W, 55 mW cm^{-2}) irradiation for 12 h.

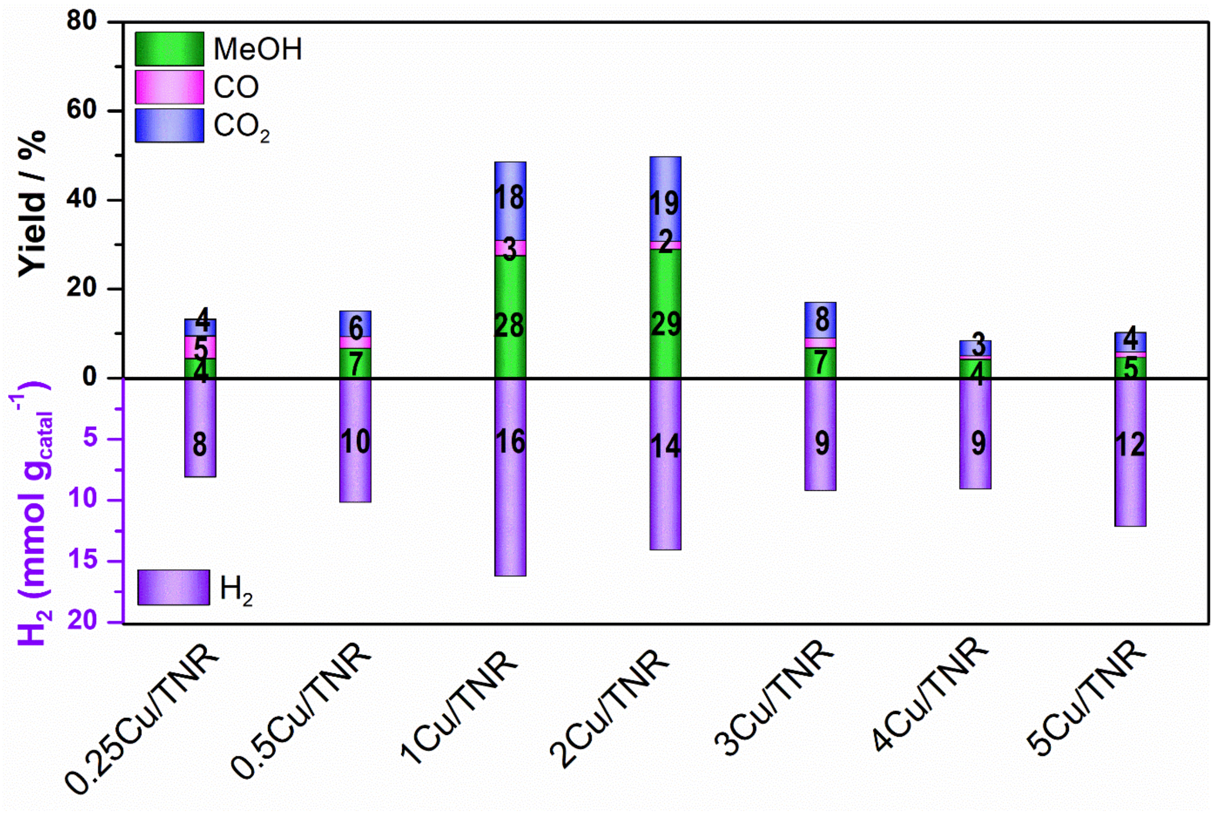

Supplementary Figure 3 The effect of copper loading. Reaction conditions: 10 mg of glycerol, 10 mg of catalyst, 0.8 mL of MeCN, 0.2 mL of water, 365 nm LED (18 W, 55 mW cm^{-2}) irradiation for 12 h.

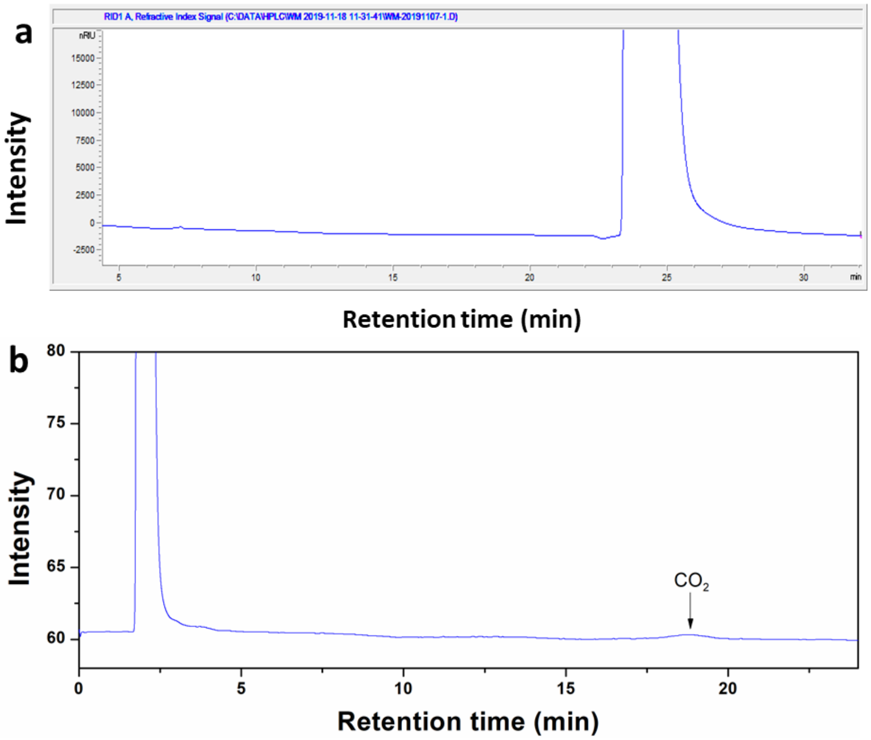

Supplementary Figure 4 The HPLC (a) and GC (b) curves. Reaction conditions: 0.8 mL of MeCN, 0.2 mL of water, 365 nm LED (18 W, 55 mW cm^{-2}), Ar, 12 h

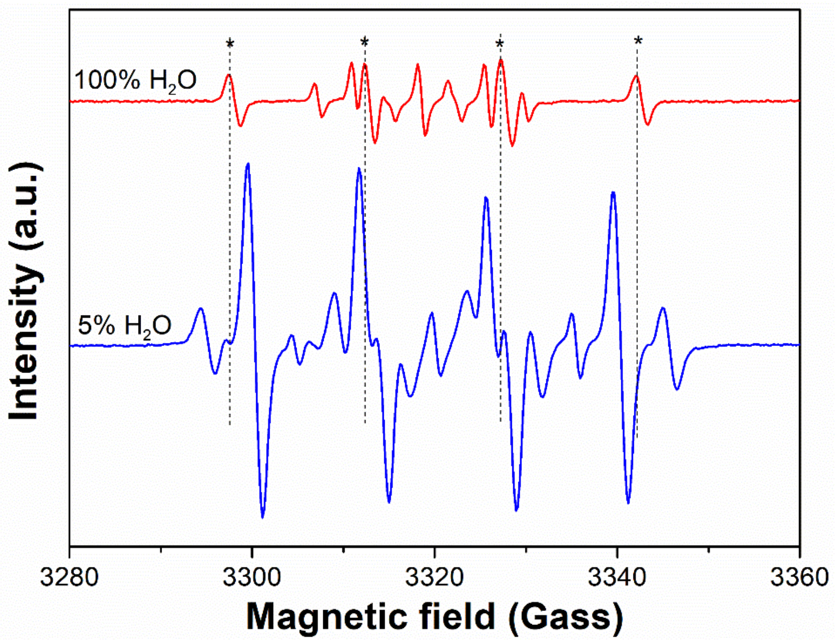

Supplementary Figure 5 EPR spectra of hydroxyl radical (*) produced by photo irradiation over 2Cu/TNR and stabilized by 5,5-dimethyl-1-pyrroline N-oxide (DMPO).

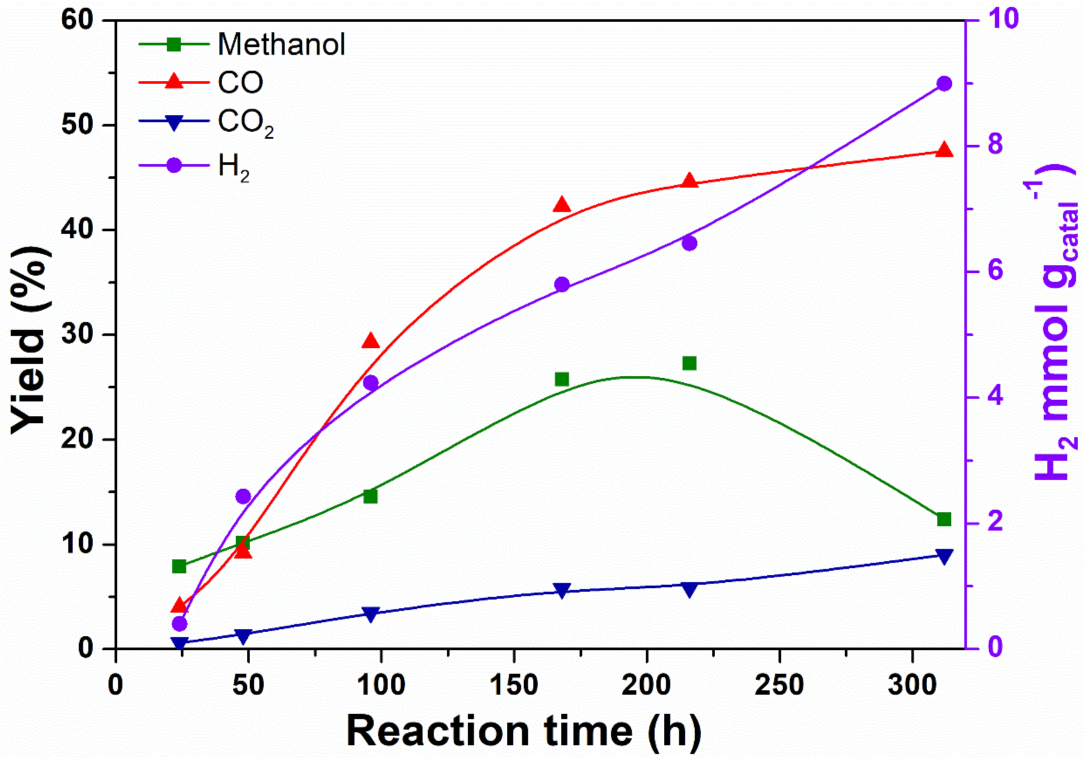

Supplementary Figure 6 The reaction time profile of photo reforming of glycerol over 0.1Cu/TNR catalyst. Reaction conditions: 10 mg of glycerol, 10 mg of 0.1Cu/TNR, 0.95 mL of MeCN, 0.05 mL of water, 365 nm LED (18 W, 55 mW cm^{-2}) irradiation.

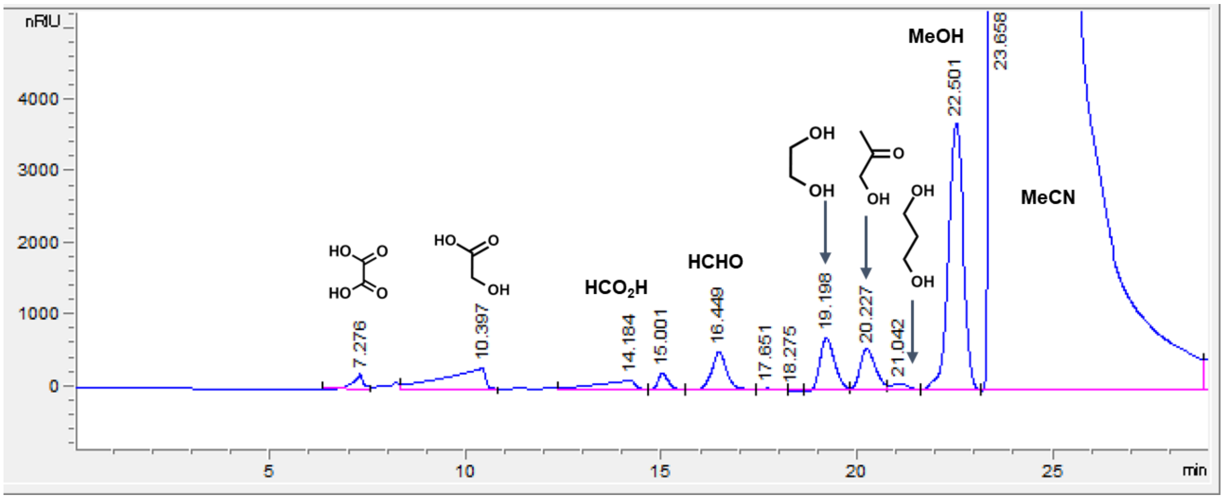

Supplementary Figure 7 HPLC spectra of the reaction solution in the photo-reforming of glycerol.

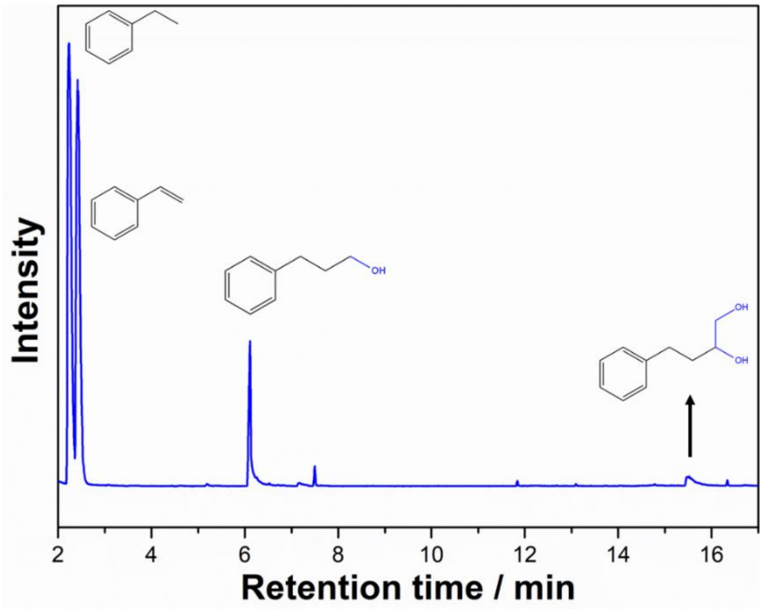

Supplementary Figure 8 The capture of radicals by styrene. The products were identified by GC-MS. Reaction conditions: 10 mg of glycerol, 50 μL of styrene, 10 mg of 2Cu/TNR, 0.8 mL of MeCN, 0.2 mL of water, 365 nm LED (18 W, 55 mW cm^{-2}) irradiation for 24 h.

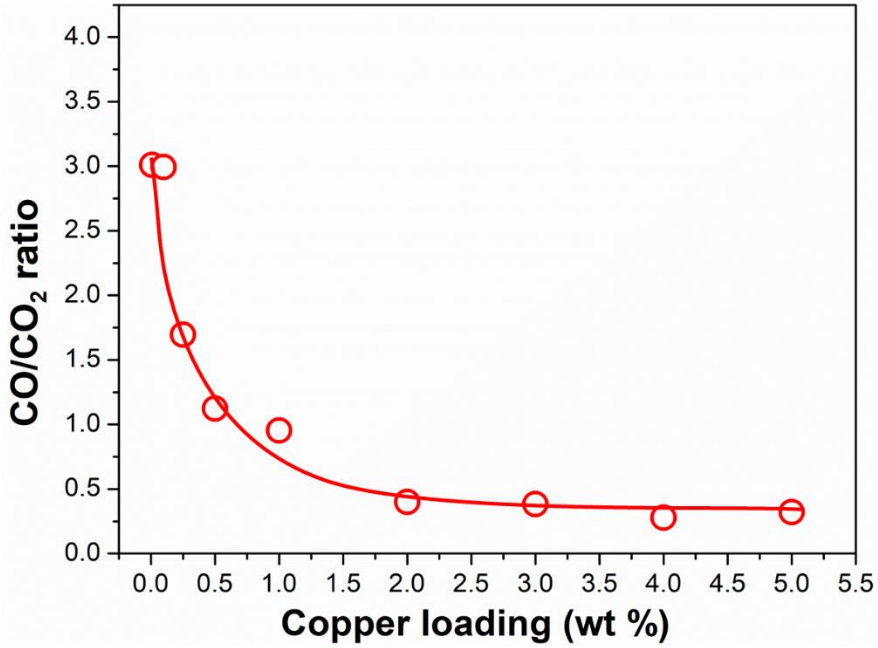

Supplementary Figure 9 The effect of copper loading on the ratio of CO/CO_2 in the photodecomposition of formic acid. Reaction conditions: 10 mg of formic acid, 10 mg of catalyst, 365 nm LED (18 W, 55 mW cm^{-2}) irradiation for 12 h.

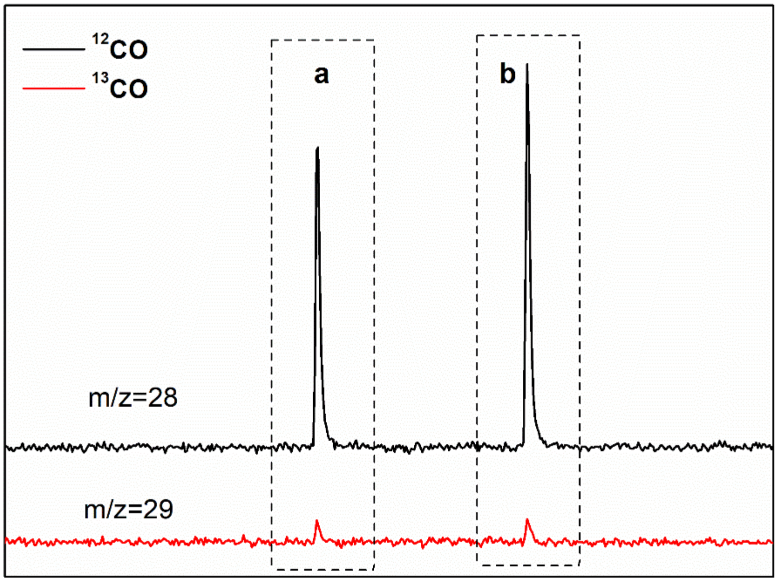

Supplementary Figure 10 The mass spectroscopy of selected m/z=28, 29. (a) Reaction conditions: 10 mg of 2Cu/TNR, 10 mg of glycerol, 0.8 mL of MeCN, 0.2 mL of water, 365 nm 9 W LED, 1mL $^{13}CO_2$, Ar atmosphere, 12 h; (b) Reaction conditions: 10 mg of 0.1Cu/TNR, 10 mg of glycerol, 0.95 mL of MeCN, 0.05 mL of water, 365 nm LED (18 W, 55 mW cm^{-2}), 1mL $^{13}CO_2$, Ar atmosphere, 24 h.

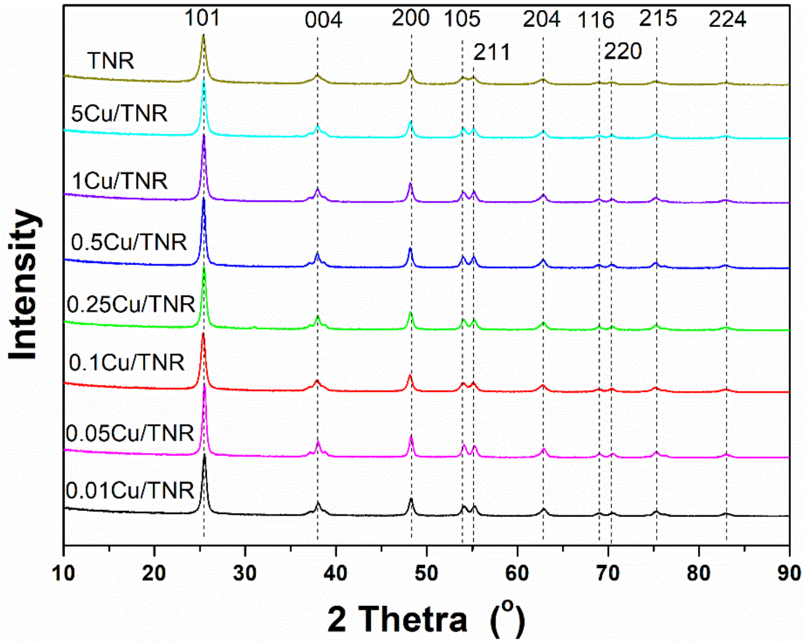

Supplementary Figure 11 XRD patterns of the Cu/TNR catalyst with different copper loadings.

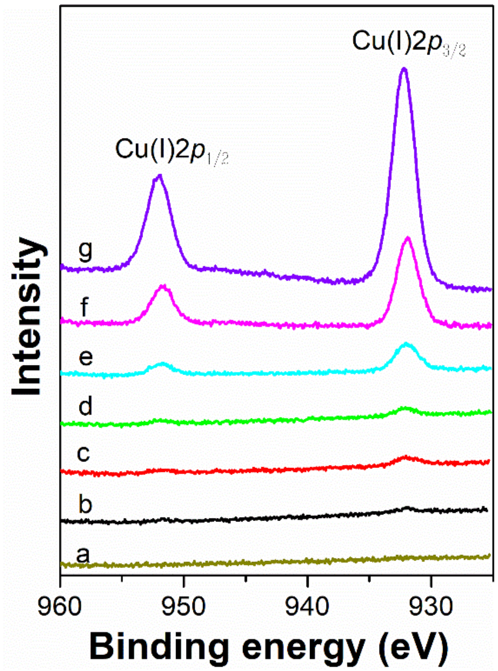

Supplementary Figure 12 XPS of copper catalyst. a:0.01Cu/TNR; b:0.05 Cu/TNR; c: 0.1 Cu/TNR; d: 0.2 Cu/TNR; e: 0.5 Cu/TNR; f:1 Cu/TNR; g: 2Cu/TNR.

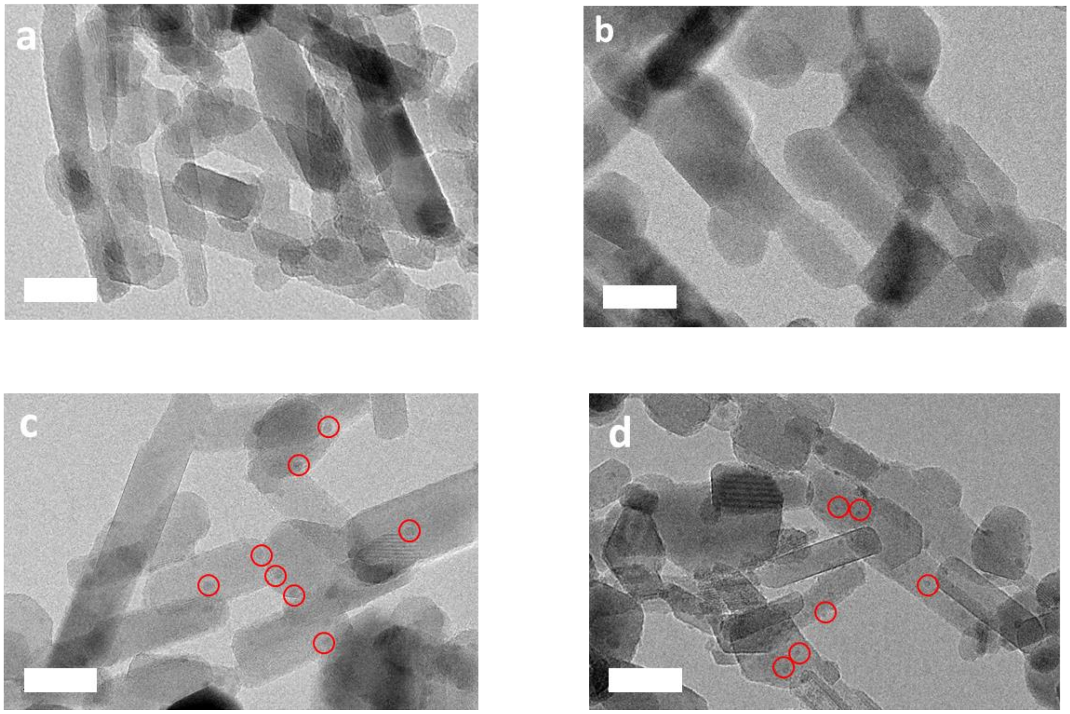

Supplementary Figure 13 TEM of Cu/TNR catalyst with different copper loading**.** (a) 0.1Cu/TNR, (b) 1Cu/TNR, (c) 2Cu/TNR and (d) 5Cu/TNR. Scale bar, 20 nm.

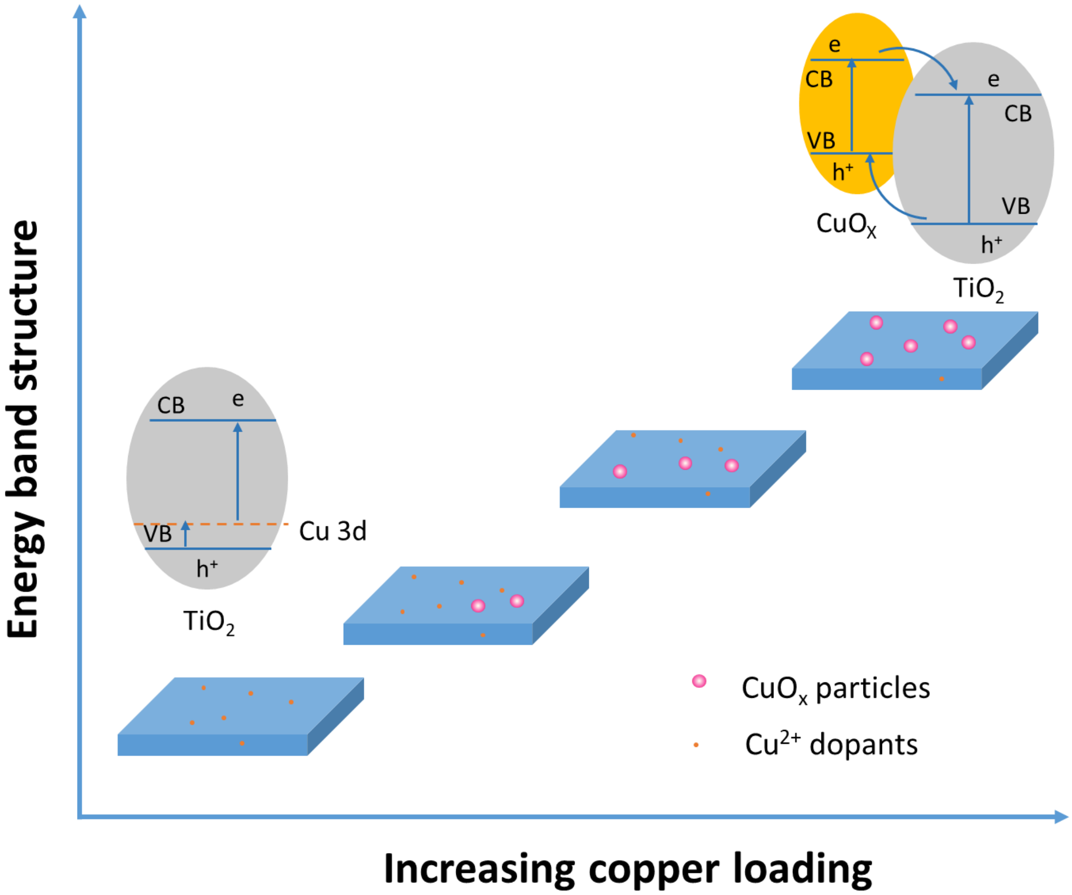

Supplementary Figure 14 Catalyst model and corresponding energy band structure.

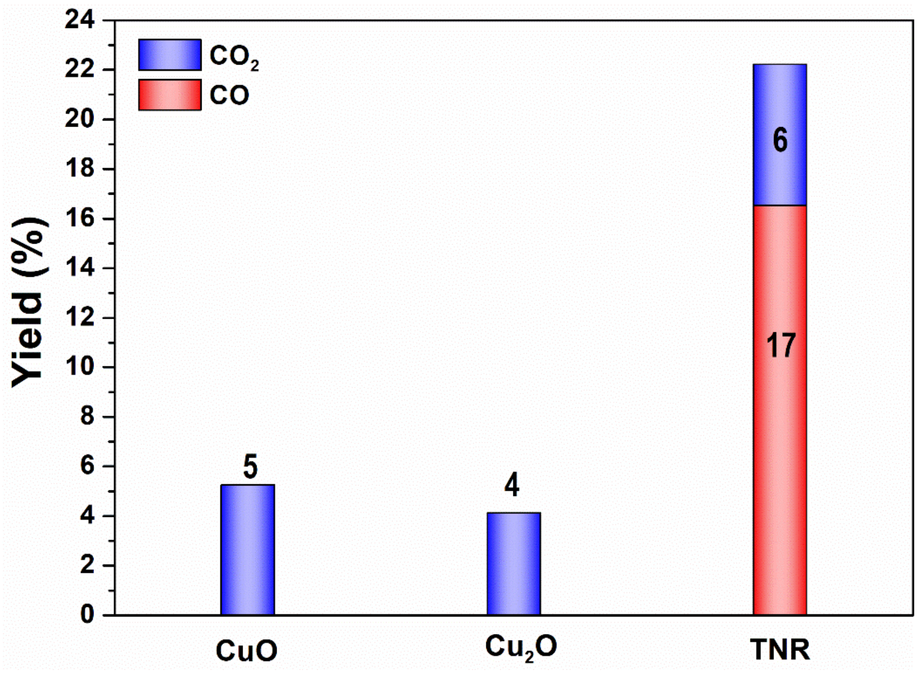

Supplementary Figure 15 The photodecomposition of formic acid. Reaction conditions: 10 mg of formic acid 10 mg of catalyst, 365 nm LED (18 W, 55 mW cm^{-2}) irradiation for 12 h.

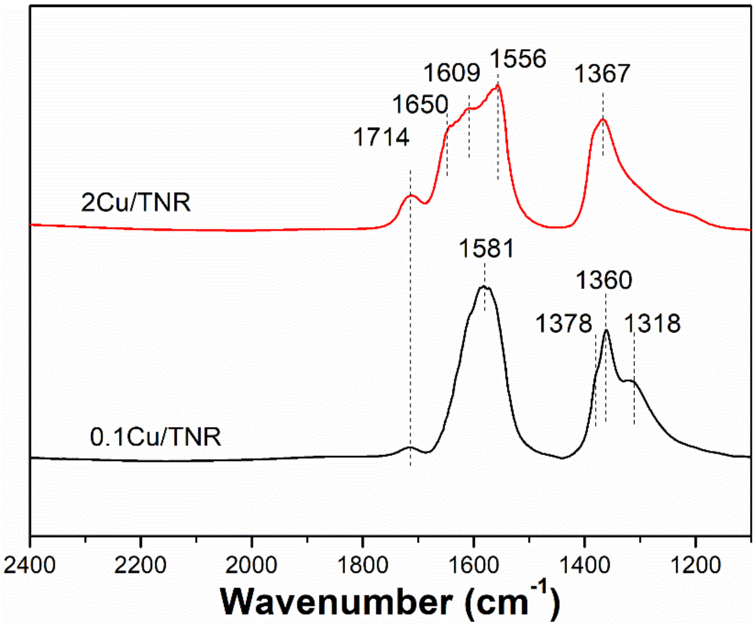

Supplementary Figure 16 Formic acid adsorption on Cu/TNR.

Supplementary Tables

Supplementary Table 1. A Summary of the photoreforming of glycerol

						Production rate/μmol$g_{cat}^{-1}h^{-1}$					
Photocatalyst	Reaction medium	Light Source	P/W (I/mW cm^{-2})	Atmosphere	T/℃	H_2	CH_4	CO	CO_2	CH_3OH	
0.5 wt% Pt/TiO_2(P25)	Glycerol (aq, 0.368mM)	Xe-arc lamp	450	Ar	40	89.9	-	-	36.7	-	Reference
0.5 wt% Pt/TiO_2(P25)	Glycerol (aq, 17.7mM)	Xe-arc lamp	450	Ar	40	1151	-	-	300	-	17
TiO_2(P25)	Glycerol (aq, 20mM)	Xe-arc lamp	450	O_2	40	-	-	-	975	-	18
TiO_2(P25)	Glycerol (aq, 20mM)	Xe-arc lamp	450	Ar	40	75	-	-	<7.5	1.25	18
0.5 wt% Pt/TiO_2(P25)	Glycerol (aq, 20mM)	Xe-arc lamp	450	O_2	40	-	-	-	2250	-	18
0.5 wt% Pt/TiO_2(P25)	Glycerol (aq, 20mM)	Xe-arc lamp	450	Ar	40	1875	-	-	450	0.42	18
1 wt% Pt/TiO_2(P25)	H_2O/Glycerol (*l* , 30:1)	Xe-arc lamp	300	Vacuum	10	4280	-	-	-	-	19
2 wt% Pt/TiO_2(P25)	Glycerol (aq, 8.3mM)	High-pressure Hg lamp	-	N_2	20	4285	-	-	1836	-	20
0.5 wt% Pd/TiO_2(P25)	H_2O/Glycerol (*l* , 1000:1)	Xe-arc lamp	400	Ar	-	892.9	-	-	-	-	21
0.5 wt% Pd/TiO_2(P25)	H_2O/Glycerol (*l*, 1000:1)	Xe-arc lamp	400	Ar	-	1075	-	-	-	-	22
1 wt% Pd/TiO_2(P25)	25% Glycerol (H_2O)	UV LED (365nm)	-	-	-	9846	-	-	-	-	23
0.5 wt% Au/TiO_2(P25)	10% Glycerol	UV Light (365nm)	100	N_2		27900	-	-	-	-	24
2 wt% Au/TiO_2(P25)	H_2O/Glycerol (*l*, 1000:1)	Xe-arc lamp	400	Ar	-	639.9	-	-	-	-	22
1 wt% Au_3Pd/TiO_2(P25)	25% Glycerol (aq)	UV LED (365nm)	(60)	Ar	-	13231	-	-	-	-	23
1 wt% Pd_3Au/TiO_2(P25)	25% Glycerol (aq)	UV LED (365nm)	(60)	Ar	-	14769	-	-	-	-	23
1 wt%$Au_{shell}Pd_{core}/TiO_2$(P25)	25% Glycerol (aq)	UV LED (365nm)	(60)	Ar	-	14923	-	-	-	-	23
1 wt% $Pd_{shell}Au_{core}/TiO_2$(P25)	25% Glycerol (aq)	UV LED (365nm)	(60)	Ar	-	19631	-	-	-	-	23
1 wt% $Pd_{shell}Au_{core}/TiO_2$(P25)	25% Crude Glycerol (aq)	UV LED (365nm)	(60)	Ar	-	10109	-	-	-	-	23
0.4 wt% CuO/TiO_2(P25)	Glycerol (aq, 1M)	Xe-arc lamp	250	Ar	-	863	-	-	50	-	25
1.25 wt% CuO/TiO_2(P25)	Crude Glycerol (aq, 1.04wt%)	UVA (340nm)	-	Ar	40	92	-	-	-	-	26
1.3 wt% CuO/TiO_2(P25)	Glycerol (aq, 0.1M)	UV LED (365nm)	4×3	N_2	-	2061	-	-	-	-	27
Cu_2O/TiO_2(P25)	Glycerol (aq, 0.1M)	Xe-arc lamp (λ>420nm)	500	-	-	240	-	-	-	-	28
0.25 wt% Sn/0.68 wt% RuO_2/TiO_2(P25)	H_2O/Glycerol (*l*, 33:1)	High-pressure Hg lamp	125	N_2	20	31500	140	7300	-	-	29
TiO_2(nanorod)	5 vol% Glycerol (aq)	Sunlight	-	N_2	-	2950	-	-	-	-	30
0.1 wt% Pt/TiO_2(74% Rutile)	Glycerol (aq, 1.3mM)	Xe-arc lamp	300	-	-	7784	-	-	-	-	31
0.5 wt% Pt/TiO_2(porous)	H_2O/Glycerol (*l*, 5:1)	High-pressure Hg lamp	500	N_2	30	6250	-	-	-	-	32
1 wt% Pt/TiO_2	Glycerol (aq, 2.7mM)	High-pressure Hg lamp	125	N_2	30	6000	18	-	2500	-	33
2 wt% Pt/TiO_2	Glycerol (aq, 0.61g/L)	High-pressure Hg lamp	-	N_2	20	8184.5	-	-	2232.1	-	34
2.1 wt% Pt/TiO_2	Glycerol (aq, 7.34M)	UV (300-400nm)	12×15	He		6335	-	-	72	-	35
4 wt% Pt/TiO_2	H_2O/Glycerol (*l* , 1:1)	Xe-arc lamp (λ>320nm)	500	-	40	2500	-	-	-	-	36
0.5 wt% Au/TiO_2(Anatase)	10% Glycerol	UV Light (365nm)	100	N_2	40	15000	-	-	-	-	24
0.5 wt% Au/TiO_2(brookite)	10% Glycerol	UV Light (365nm)	100	N_2	40	13800	-	-	-	-	24
0.5 wt% Au/TiO_2(Rutile)	10% Glycerol	UV Light (365nm)	100	N_2	40	3200	-	-	-	-	24
1 wt% Au/TiO_2(Anatase)	H_2O/Glycerol (*l* , 1:1)	Xe-arc lamp	300	Ar	25	4900	-	-	-	-	37
1 wt% Au/TiO_2/cordierite honeycomb	H_2O/Glycerol (*l* , 1:1 mol)	UV LED (365±5nm)	4×12	Ar	25	768	-	-	-	-	38
1 wt% Au/TiO_2/cordierite honeycomb	H_2O/Bio-Glycerol (*l* , 1:1 mol)	UV LED (365±5nm)	4×12	Ar	25	300	-	-	-	-	38
2 wt% Pt/F-TiO_2(nanosheet)	Glycerol (aq, 0.1M)	Xe-arc lamp	350	N_2	-	8455	-	-	-	-	39
Pt/B-TiO_2	5% Glycerol (aq)	Xe-arc lamp	300	N_2	-	4133	-	-	-	-	40
Pt/N-TiO_2	5% Glycerol (aq)	Xe-arc lamp	300	N_2	-	5133	1.13	18.1	496.6	0.9	40
Pt/(B, N)-TiO_2	5% Glycerol (aq)	Xe-arc lamp	300	N_2	-	8200	0.92	1.85	1040	1.04	40
0.3 wt% Pt/1 mol% Gd-TiO_2	H_2O/Glycerol (*l* , 50:1)	Hg lamp	300	Ar	77~82	12075	-	-	-	-	41
10 mol% Cu/TiO_2	10 vol% Glycerol (aq) + 2M NaOH	Halogen	500	Ar	24	5772	-	-	-	-	42
1wt% Cu_2O/TiO_2	5% Glycerol (aq)	Hg lamp	250	N_2	-	16656	-	-	-	-	43
1.5 wt% Cu_2O/TiO_2(nanorod)	5 vol% Glycerol (aq)	Sunlight	-	N_2	-	50339	-	-	-	-	30
1wt% CuO_x/TiO_2	Glycerol (aq, 1M)	Xe-arc lamp	150	Ar	-	580	-	-	-	-	44
1wt% CuO_x/TiO_2	Glycerol (aq, 1M)	High-pressure Hg lamp	125	Ar	-	1200	-	-	200	-	44
2.5wt% CuO_x/TiO_2	Glycerol (aq, 1M)	Hg lamp	125	Ar	20	970	-	-	-	-	45
1.5 wt% CuO/TiO_2(nanotube)	5 vol% Glycerol (aq)	Sunlight	-	N_2	-	99823	-	-	-	-	46
2 wt% CuO/TiO_2(Anatase/Rutile=7:3)	5% Glycerol (aq)	High-pressure Hg lamp	500	N_2	50	1370	300	140	70	-	47
3wt% RGO-1wt% Cu_2O/TiO_2	5% Glycerol (aq)	Hg lamp	250	N_2	-	110968	-	-	-	-	43
2 wt% NiO/TiO_2(Anatase/Rutile=7:3)	5% Glycerol (aq)	High-pressure Hg lamp	500	N_2	50	1230	19	106	41	-	47
10 wt% NiO/TiO_2	H_2O/Glycerol (*l* , 5:1)	High-pressure Hg lamp	500	N_2	50	900	-	130	590	-	48
2 wt% CoO/TiO_2(Anatase/Rutile=7:3)	5% Glycerol (aq)	High-pressure Hg lamp	500	N_2	50	660	60	100	50	-	47
NiO	H_2O/Glycerol (*l* , 5:1)	High-pressure Hg lamp	500	N_2	50	91	-	2124	917	-	48
3wt% RGO/TiO_2	5% Glycerol (aq)	Hg lamp	250	N_2	-	8226	-	-	-	-	43
ZnO(Nanotube arrays)	10 vol% Glycerol (aq)	Xe-arc lamp	350	-	25	38	-	-	-	-	49
RGO/ZnO	5% Glycerol (aq)	Xe-arc lamp	300	Ar	RT	82	-	-	22	-	50
12wt% RGO/ZnO	5% Glycerol (aq)	Xe-arc lamp	300	Ar	RT	92	-	-	-	-	51
ZnS	10 vol% Glycerol (aq)	Xe-arc lamp	350	-	25	232	-	-	-	-	49
ZnS/ZnO(Nanotube arrays)	10 vol% Glycerol (aq)	Xe-arc lamp	350	-	25	384	-	-	-	-	49
ZnS/ZnO(Nanorod)	7% Glycerol (aq)	High-pressure Hg lamp	125	Ar	-	2609	-	-	-	-	52
ZnS/ZnO(Nanorod)	7% Glycerol (aq)	Xe-arc lamp	500	Ar	-	388	-	-	-	-	52
ZnO@Bi_2S_3/ZnS/RGO	5% Glycerol (aq)	Xe-arc lamp	300	Ar	RT	310	-	-	80	-	50
0.5 wt% Pt/3 wt% Au-WO_3	Glycerol (aq, 2mM)	Xe-arc lamp (450-600nm)	(83)	Ar	25	132	-	-	56	-	53
Bi_2WO_6	H_2O/Glycerol (*l* , 1:1)	Xe-arc lamp	300	Ar	-	7400	-	-	-	-	54
0.2% wt Pt/CdS	10 vol% Glycerol (aq)	Xe-arc lamp (>420nm)	300	Vacuum	10±5	170	-	-	-	-	55
0.2% wt MoS_2/CdS	10 vol% Glycerol (aq)	Xe-arc lamp (>420nm)	300	Vacuum	10±5	370	-	-	-	-	55
0.5wt% $Cd_{0.5}Zn_{0.5}S$	1.368M Glycerol (aq) + 1M NaOH	High-pressure Hg lamp (>420nm)	250	N_2	-	630	-	-	-	-	56
$Cd_{0.5}Zn_{0.5}S$: (γ-$Zn(OH)_2$)	H_2O/Glycerol (*l* , 1:1) + 1.5M NaCl	Hg-Xe arc lamp (418~IR)	500	Ar	-	239	-	-	-	-	57
2wt%Cu/TNR	Glycerol (MeCN-H_2O, 8:2)	UV LED (365nm)	(55)	Ar	RT	1308	-	52	570	788	This work
0.1wt%Cu/TNR	Glycerol (MeCN-H_2O, 9.5:0.5)	UV LED (365nm)	(55)	Ar	RT	36	-	82	11	46	This work

Supplementary Table 2. Experimental results for the photo-reforming of glycerol

Reaction time (h)	6	12	16	24	30	36	50
Conversion (%)	43	84	100	100	100	100	100
Carbon distribution (%)							
CO	1.4	1.7	2.9	4.1	4.4	4.0	5.5
CO_2	10.2	19.1	26.0	32.5	35.0	35.9	49.6
CH_4	0	0	0	0	0	0	0.7
Glycerol	57.0	16.0	0	0	0	0	0
Oxalic acid	0.2	0.1	0.2	0.2	0.2	0.2	0.4
Glycolic acid	3.8	1.9	1.0	0.7	0.9	1.1	1.1
Formic acid	0	0	0.6	0.9	0.9	4.3	0
Formaldehyde	0	0	2.4	0.8	1.1	0.8	2.7
Ethylene glycol	4.8	6.8	3.5	0.9	0.3	0.1	0.1
Hydroxypropanone	2.0	2.0	1.8	1.1	1.9	1.9	2.8
1, 3-Propanediol	0.6	0	0	0.2	0.2	0	0
Methanol	8.8	29.0	39.0	39.3	35.2	29.1	16.0
Carbon balance (%)	88.8	76.5	77.4	80.7	80.1	77.4	79.4

Reaction conditions: 10 mg of glycerol, 10 mg of 2Cu/TNR, 0.8 mL of MeCN, 0.2 mL of water, 365 nm LED (18 W, 55 mW cm^{-2}) irradiation.

Supplementary Table 3. Experimental results for the photo-reforming of other polyols over 2Cu/TNR

Substrates	Ethylene glycol	1, 2-Propanediol	Erythritol	Xylitol	Sorbitol	Fructose
Reaction time (h)	16	12	72	60	54	54
Conversion (%)	95	100	100	100	100	100
Carbon distribution (%)						
Substrate	5.0	0	0	0	0	0
CO	2.0	0.8	12.8	11.4	6.0	2.6
CO_2	37.5	11.7	41.1	43.1	37.5	33.0
CH_4	0	0.8	0	0	0	0
Oxalic acid	0.1	0.1	0.2	0.4	0.5	0.9
Glycolic acid	2.0	0.3	1.2	1.1	1.7	8.0
Formic acid	0.8	1.6	1.0	1.1	4.3	4.6
Formaldehyde	2.7	1.5	6.3	0.5	0.8	2.2
Ethylene glycol	\	0	4.1	1.3	2.2	8.4
Hydroxypropanone	1.6	18.6	1.6	0.4	1.8	3.6
1, 3-Propanediol	0	0	0	0.3	0.5	0
Methanol	31.3	29.8	19.0	14.7	21.5	16.5
Carbon balance (%)	83.0	72.6	87.3	74.3	76.8	79.8

Reaction conditions: 10 mg of substrates, 10 mg of 2Cu/TNR, 0.8 mL of MeCN, 0.2 mL of water, 365 nm LED (18 W, 55 mW cm^{-2}) irradiation.

Supplementary Table 4. Experimental results for the photo-reforming of polyols 0.1Cu/TNR.

Substrates	Ethylene glycol	Glycerol	Erythritol	Xylitol	Sorbitol	Glucose	Fructose	Sucrose
Reaction time (h)	96	168	288	288	288	144	144	144
Conversion (%)	99.9	98.3	100	100	100	100	100	100
Carbon distribution (%)								
Substrate	0.1	1.7	0	0	0	0	0	0
CO	40.1	42.0	51.9	47.1	55.7	49.1	43.7	31.8
CO_2	5.1	5.7	6.0	6.2	7.2	5.3	11.2	15.8
CH_4	0	0	1.9	0.7	2.0	2.5	5.4	0
Oxalic acid	0.3	0	0	0.7	0.2	0	0	0.6
Glycolic acid	1.0	1.3	3.5	4.0	3.1	3.3	3.0	1.4
Formic acid	3.2	0	0	8.3	9.9	9.1	13.8	7.9
Formaldehyde	0.6	0	4.2	0	0	0	0	4.6
Ethylene glycol	\	1.0	0	0.1	0.2	0	0	0.3
Hydroxypropanone	0.1	0	0	0.6	0.6	0.7	0	1.2
1, 3-Propanediol	0.4	2.8	0	0	0.1	4.4	5.1	4.8
Methanol	31.2	23.8	9.6	6.1	6.8	8.4	8.2	8.5
Carbon balance (%)	82.1	78.3	77.1	73.8	85.8	82.8	90.4	76.9

Reaction conditions: 10 mg of substrates, 10 mg of 0.1Cu/TNR, 0.95 mL of MeCN, 0.05 mL of water, 365 nm LED (18 W, 55 mW cm^{-2}) irradiation.

Supplementary Table 5. Experimental results for the conversion of cellulose.

Substrates	Cellulose	Substrates	Cellulose hydrogenolysis solution	
Reaction conditions	Raney Ni, 4 MPa H_2, H_2O, 245 ºC, 30 min	Reaction conditions	2Cu/TNR, MeCN/H_2O (8:2), 12 h	0.1Cu/TNR, MeCN/H_2O (9.5:0.5), 72 h
Conversion (%)	100			
Products distribution (%)		Carbon distribution (%)		
Ethylene glycol	63.6	CO	0.5	45.0
1, 2-Propanediol	7.7	CO_2	30.0	5.6
Glycerol	2.2	CH_4	0	8.7
Erythritol	3.3	Oxalic acid	1.6	2.2
Sorbitol	6.9	Glycolic acid	2.0	10.8
		Formic acid	0	0
		Formaldehyde	7.7	0
		Ethylene glycol	10.8	0
		Hydroxypropanone	13.8	0
		1, 3-Propanediol	0	0
		Methanol	14.0	2.4
Carbon balance (%)	84	Carbon balance (%)[a]	80.4	74.1

[a] The carbon balance is calculated based on cellulose.

Supplementary Table 6. Experimental results for the photo-reforming of beech.

Substrates	Beech	Substrates	Beech hydrolysis solution
Reaction conditions	H_2SO_4, 4 MPa H_2, H_2O, 100 ºC, 6 h	Reaction conditions	0.1Cu/TNR, MeCN/H_2O (9.5:0.5), 6 h
Conversion (%)	100		
Carbon content in beech (wt%)	44.9		
Carbon distribution (%)		Carbon distribution (%)	
Glucose	18.4	CO	25.2
Xylose	12.1	CO_2	6.3
Fructose	5.1	CH_4	0
Formic acid	2.5	Oxalic acid	1.5
Soluble polymer	21.9	Glycolic acid	1.8
Lignin	40	Formic acid	10.1
		Formaldehyde	1.3
		Ethylene glycol	1.4
		Hydroxypropanone	1.8
		1, 3-Propanediol	0
		Methanol	6.9
Carbon balance (%)[a]	84	Carbon balance (%)**[a]**	56.3

[a] The carbon balance is calculated based on cellulose and hemicellulose.

Supplementary References

1 Jiang, Z. *et al.* Solvothermal synthesis of N-doped TiO_2 nanotubes for visible-light-responsive photocatalysis. *Chem. Commun.* **47**, 6372-6374, (2008).

2 Li, R. *et al.* Spatial separation of photogenerated electrons and holes among {010} and {110} crystal facets of $BiVO_4$. *Nat. Commun.* **4**, 1432, (2013).

3 Zhang, Y. H., Zhang, N., Tang, Z. R. & Xu, Y. J. Identification of Bi_2WO_6 as a highly selective visible-light photocatalyst toward oxidation of glycerol to dihydroxyacetone in water. *Chem Sci* **4**, 1820, (2013).

4 Guan, M. *et al.* Vacancy associates promoting solar-driven photocatalytic activity of ultrathin bismuth oxychloride nanosheets. *J. Am. Chem. Soc.* **135**, 10411-10417, (2013).

5 Xiong, Y. S. *et al.* Growth and phase-transformation mechanisms of nanocrystalline CdS in Na2S solution. *J Phys Chem C* **112**, 9229-9233, (2008).

6 Kresse, G. & Furthmuller, J. Efficiency of ab-initio total energy calculations for metals and semiconductors using a plane-wave basis set. *Comp Mater Sci* **6**, 15-50, (1996).

7 Perdew, J. P., Burke, K. & Ernzerhof, M. Generalized gradient approximation made simple. *Phys. Rev. Lett.* **77**, 3865-3868, (1996).

8 Kresse, G. & Joubert, D. From ultrasoft pseudopotentials to the projector augmented-wave method. *Phys. Rev. B* **59**, 1758-1775, (1999).

9 Monkhorst, H. J. & Pack, J. D. Special Points for Brillouin-Zone Integrations. *Phys. Rev. B* **13**, 5188-5192, (1976).

10 Dudarev, S. L., Botton, G. A., Savrasov, S. Y., Humphreys, C. J. & Sutton, A. P. Electron-energy-loss spectra and the structural stability of nickel oxide: An LSDA+U study. *Phys. Rev. B* **57**, 1505-1509, (1998).

11 Burdett, J. K., Hughbanks, T., Miller, G. J., Richardson, J. W. & Smith, J. V. Structural Electronic Relationships in Inorganic Solids - Powder Neutron-Diffraction Studies of the Rutile and Anatase Polymorphs of Titanium-Dioxide at 15 and 295-K. *J. Am. Chem. Soc.* **109**, 3639-3646, (1987).

12 Si, X. Q. *et al.* A strategy for generating high-quality cellulose and lignin simultaneously from woody biomass. *Green Chem.* **19**, 4849-4857, (2017).

13 Kau, L. S., Spirasolomon, D. J., Pennerhahn, J. E., Hodgson, K. O. & Solomon, E. I. X-Ray Absorption-Edge Determination of the Oxidation-State and Coordination-Number of Copper - Application to the Type-3 Site in Rhus-Vernicifera Laccase and Its Reaction with Oxygen. *J. Am. Chem. Soc.* **109**, 6433-6442, (1987).

14 Fukumi, K. *et al.* Structural investigation on implanted copper ions in silica glass by XAFS spectroscopy. *J. Non-Cryst. Solids* **238**, 143-151, (1998).

15 Liu, Y. X., Wang, Z. L. & Huang, W. X. Influences of TiO_2 phase structures on the structures and photocatalytic hydrogen production of CuOx/TiO_2 photocatalysts. *Appl. Surf. Sci.* **389**, 760-767, (2016).

16 Kondarides, D. I., Daskalaki, V. M., Patsoura, A. & Verykios, X. E. Hydrogen Production by Photo-Induced Reforming of Biomass Components and Derivatives at Ambient Conditions. *Catal. Lett.* **122**, 26-32, (2008).

17 Daskalaki, V. M. & Kondarides, D. I. Efficient production of hydrogen by photo-induced reforming of glycerol at ambient conditions. *Catal. Today* **144**, 75-80, (2009).

18 Panagiotopoulou, P., Karamerou, E. E. & Kondarides, D. I. Kinetics and mechanism of glycerol photo-oxidation and photo-reforming reactions in aqueous TiO_2 and Pt/TiO_2 suspensions. *Cata. Today* **209**, 91-98, (2013).
19 Jiang, X., Fu, X., Zhang, L., Meng, S. & Chen, S. Photocatalytic reforming of glycerol for H_2 evolution on Pt/TiO_2: fundamental understanding the effect of co-catalyst Pt and the Pt deposition route. *J. Mater. Chem. A* **3**, 2271-2282, (2015).
20 Shiragami, T., Tomo, T., Matsumoto, T. & Yasuda, M. Structural Dependence of Alcoholic Sacrificial Agents on TiO_2-Photocatalytic Hydrogen Evolution. *Bulletin Chem. Soc. Japan* **86**, 382-389, (2013).
21 Bahruji, H. *et al.* Sustainable H_2 gas production by photocatalysis. *Journal of Photochem. Photobio. A: Chem.* **216**, 115-118, (2010).
22 Bowker, M., Davies, P. R. & Al-Mazroai, L. S. Photocatalytic Reforming of Glycerol over Gold and Palladium as an Alternative Fuel Source. *Catal. Lett.* **128**, 253-255, (2008).
23 Su, R. *et al.* Designer Titania-Supported Au-Pd Nanoparticles for Efficient Photocatalytic Hydrogen Production. *ACS Nano* **8**, 3490-3497, (2014).
24 Chen, W.-T. *et al.* Effect of TiO_2 polymorph and alcohol sacrificial agent on the activity of Au/TiO_2 photocatalysts for H_2 production in alcohol–water mixtures. *J. Catal.* **329**, 499-513, (2015).
25 Petala, A., Ioannidou, E., Georgaka, A., Bourikas, K. & Kondarides, D. I. Hysteresis phenomena and rate fluctuations under conditions of glycerol photo-reforming reaction over CuOx/TiO2 catalysts. *Appl. Catal. B: Environ.* **178**, 201-209, (2015).
26 Skaf, D. W., Natrin, N. G., Brodwater, K. C. & Bongo, C. R. Comparison of Photocatalytic Hydrogen Production from Glycerol and Crude Glycerol Obtained from Biodiesel Processing. *Catal. Lett.* **142**, 1175-1179, (2012).
27 Yu, J., Hai, Y. & Jaroniec, M. Photocatalytic hydrogen production over CuO-modified titania. *J. Colloid Inter. Sci.* **357**, 223-228, (2011).
28 Kum, J. M., Park, Y. J., Kim, H. J. & Cho, S. O. Plasmon-enhanced photocatalytic hydrogen production over visible-light responsive Cu/TiO_2. *Nanotechnology* **26**, 125402, (2015).
29 Gu, Q. *et al.* Single-site Sn-grafted Ru/TiO_2 photocatalysts for biomass reforming: Synergistic effect of dual co-catalysts and molecular mechanism. *J. Catal.* **303**, 141-155, (2013).
30 Praveen Kumar, D. *et al.* Cu_2O-sensitized TiO_2 nanorods with nanocavities for highly efficient photocatalytic hydrogen production under solar irradiation. *Solar Energ. Mater. Solar Cells* **136**, 157-166, (2015).
31 Xu, Q. *et al.* Enhancing hydrogen production activity and suppressing CO formation from photocatalytic biomass reforming on Pt/TiO_2 by optimizing anatase–rutile phase structure. *J. Catal.* **278**, 329-335, (2011).
32 Sun, W., Zhang, S., Liu, Z., Wang, C. & Mao, Z. Studies on the enhanced photocatalytic hydrogen evolution over Pt/PEG-modified TiO_2 photocatalysts. *Int. J. Hydrog. Energy.* **33**, 1112-1117, (2008).
33 Fu, X. *et al.* Photocatalytic reforming of C3-polyols for H_2 production Part (I). Role of their OH groups. *Appl. Catal. B: Environ.***106**, 681-688, (2011).
34 Shiragami, T. *et al.* Pentose Acting as a Sacrificial Multielectron Source in Photocatalytic Hydrogen Evolution from Water by Pt-doped TiO_2. *Chem. Lett.* **41**, 29-31, (2012).

35 López, C. R. *et al.* Comparative study of alcohols as sacrificial agents in H_2 production by heterogeneous photocatalysis using Pt/TiO_2 catalysts. *J. Photochem. Photobio. A: Chem.* **312**, 45-54, (2015).
36 Sakata, T., Kawai, T. & Hashimoto, K. Photochemical diode model of Pt/TiO_2 particle and its photocatalytic activity. *Chem. Phys. Lett.* **88**, 50-54, (1982).
37 Gärtner, F. *et al.* Hydrogen Evolution from Water/Alcohol Mixtures: Effective In Situ Generation of an Active Au/TiO_2 catalyst. *ChemSusChem* **5**, 530-533, (2012).
38 Taboada, E., Angurell, I. & Llorca, J. Visible-Light-Induced Hydrogen and Oxygen Formation over Pt/Au/ WO_3 Photocatalyst Utilizing Two Types of Photoabsorption Due to Surface Plasmon Resonance and Band-Gap Excitation. *J. Photochem. Photobio. A: Chem.* **281**, 35-39, (2014).
39 Yu, J., Qi, L. & Jaroniec, M. Hydrogen Production by Photocatalytic Water Splitting over Pt/TiO2 Nanosheets with Exposed (001) Facets. *J. Phys. Chem. C* **114**, 13118-13125, (2010).
40 Luo, N. *et al.* Photo-catalytic conversion of oxygenated hydrocarbons to hydrogen over heteroatom-doped TiO_2 catalysts. *Int. J. Hydrog. Energy* **34**, 125-129, (2009).
41 Zalas, M. & Laniecki, M. Photocatalytic hydrogen generation over lanthanides-doped titania. *Sol. Energy Mater. Sol. C.* **89**, 287-296, (2005).
42 Bashiri, R., Mohamed, N. M., Kait, C. F. & Sufian, S. Hydrogen production from water photosplitting using Cu/TiO_2 nanoparticles: Effect of hydrolysis rate and reaction medium. *Int. J. Hydrog. Energy* **40**, 6021-6037, (2015).
43 Babu, S. G. *et al.* Influence of electron storing, transferring and shuttling assets of reduced graphene oxide at the interfacial copper doped TiO_2 p–n heterojunction for increased hydrogen production. *Nanoscale* **7**, 7849-7857, (2015).
44 Montini, T. *et al.* Nanostructured Cu/TiO_2 Photocatalysts for H_2 Production from Ethanol and Glycerol Aqueous Solutions. *ChemCatChem* **3**, 574-577, (2011).
45 Gombac, V. *et al.* CuOx-TiO_2 Photocatalysts for H2 Production from Ethanol and Glycerol Solutions. *J. Phys. Chem. A* **114**, 3916-3925, (2010).
46 Praveen Kumar, D. *et al.* Nano-size effects on CuO/TiO2 catalysts for highly efficient H2 production under solar light irradiation. *Chem. Commun.* **49**, 9443-9445, (2013).
47 Fujita, S.-i., Kawamori, H., Honda, D., Yoshida, H. & Arai, M. Photocatalytic hydrogen production from aqueous glycerol solution using NiO/TiO2 catalysts: Effects of preparation and reaction conditions. *Appl. Catal. B: Environ.* **181**, 818-824, (2016).
48 Liu, R., Yoshida, H., Fujita, S.-i. & Arai, M. Photocatalytic hydrogen production from glycerol and water with NiOx/TiO2 catalysts. *Appl. Catal. B: Environ.* **144**, 41-45, (2014).
49 Bao, D. *et al.* ZnO/ZnS Heterostructured Nanorod Arrays and Their Efficient Photocatalytic Hydrogen Evolution. *Chemistry* **21**, 12728-12734, (2015).
50 Xitao, W., Rong, L. & Kang, W. Synthesis of ZnO@ZnS–Bi_2S_3 core–shell nanorod grown on reduced graphene oxide sheets and its enhanced photocatalytic performance. *J. Mater. Chem. A* **2**, 8304-8313, (2014).
51 Lv, R. *et al.* Facile synthesis of ZnO nanorods grown on graphene sheets and its enhanced photocatalytic efficiency. *J. Chem. Technol. Biotechnol.* **90**, 550-558, (2015).
52 Sang, H. X., Wang, X. T., Fan, C. C. & Wang, F. Enhanced photocatalytic H_2 production from glycerol solution over ZnO/ZnS core/shell nanorods prepared by a low temperature route. *Int. J. Hydrog. Energy* **37**, 1348-1355, (2012).

53 Tanaka, A., Hashimoto, K. & Kominami, H. Visible-Light-Induced Hydrogen and Oxygen Formation over Pt/Au/WO_3 Photocatalyst Utilizing Two Types of Photoabsorption Due to Surface Plasmon Resonance and Band-Gap Excitation. *J. Am. Chem. Soc.* **136**, 586-589, (2014).
54 Panmand, R. P. *et al.* Self-assembled hierarchical nanostructures of Bi_2WO_6 for hydrogen production and dye degradation under solar light. *CrystEngComm* **17**, 107-115, (2015).
55 Zong, X. *et al.* Photocatalytic H_2 Evolution on MoS_2/CdS Catalysts under Visible Light Irradiation. *J. Phys. Chem. C* **114**, 1963-1968, (2010).
56 Peng, S., Ding, M., Yi, T., Zhan, Z. & Li, Y. Photocatalytic Hydrogen Evolution and Decomposition of Glycerol over $Cd_{0.5}Zn_{0.5}S$ Solid Solution Under Visible Light Irradiation. *Environ.Prog. Sustain. Energy* **35**, 141-148, (2016).
57 Lopes, P. A. L., Mascarenhas, A. J. S. & Silva, L. A. Sonochemical synthesis of $Cd_{1-x}Zn_xS$ solid solutions for application in photocatalytic reforming of glycerol to produce hydrogen. *J. Alloy. Comp.* **649**, 332-336, (2015).
