# Supplementary material for: Photo splitting of bio-polyols and sugars to methanol and syngas
Source: Nat Commun. 2020 Feb 27;11:1083. doi: 10.1038/s41467-020-14915-8 (PMC7046649; doi:10.1038/s41467-020-14915-8)
Supplement: Supplementary file 2 — Peer Review File [file 41467_2020_14915_MOESM2_ESM.pdf]

Reviewers' comments:

Reviewer #1 (Remarks to the Author):

This manuscript reported a facile way for the conversion of biomass-derived polyols, sugars, cellulose and even raw wood sawdust with the Cu/TNT as photocatalyst into methanol and syngas (CO+H2) via photo-splitting at room temperature. The results showed that Cu dispersed on titanium oxide nanorods (TNR) rich in defects were effective for the selective C−C bond cleavage to methanol. High yield of methanol and co-production of syngas in the gas phase was obtained via controlling the energy band structure of Cu/TNR. The work is interesting although with few ambiguous claims. It is recommended for publication in Nature Communication after major revision. Specific comments are as follows.

1. What's the role of acetonitrile in this reaction? The yields of H2 and methanol were changed with the variation of solvent according to the data in the manuscript. What about the products with the water only as the solvent?
2. As the authors claimed that "The gas product distribution is dominantly controlled by the decomposition way of formic acid.", the authors should pay close attention to the yield of formic acid in the solution. What's the yield of formic acid in the solution? Is it associated with the amount of Cu on the TNT?
3. The FTIR information of formic acid absorbed on the Cu/TNT samples should be supplied for clarifying the mechanism of dehydrogenation and dehydration of formic acid.

Reviewer #2 (Remarks to the Author):

In their manuscript, Wang et al. report on the photocatalytic reforming of biomass-derived compounds to methanol and syngas. There is a growing interest in generating well-defined organic feedstock chemicals from biomass under mild conditions. Currently, there are only a handful of known examples for such a process, and most of these operate under rather harsh conditions and produce mostly hydrogen. The results presented here are exciting, as they show efficient production of methanol at near-ambient conditions using a simple photocatalyst based on inexpensive Cu-doped TiO2. While the performance of this system is promising, there are a number of issues that need to be addressed before publication can be considered.

The work on "real" biomass, rather than refined biomass-derived compounds deserves some discussion here. The authors claim that their work was superior to other processes for biomass utilisation, because of the lower temperatures and pressures required. However, in order to be able to convert true biomass with their system, a pre-treatment at 240 °C and 40 bars hydrogen is needed, which is not much lower than the 300 °C deemed too high in the introduction. Please comment on what the actual advantage of the present work is. The statement in the conclusion that "this process requires neither high pressure hydrogen nor high temperature" is clearly wrong and should be removed. The authors alternatively use

concentrated sulphuric acid, which subsequently must be removed with stoichiometric amounts of $Ba(OH)_2$, which clearly contradicts the claim of being more atom-efficient or milder than other processes. The title claiming "photo splitting of … cellulose" is misleading, since the cellulose is never directly exposed to photocatalysis, but the sugars generated from conventional processing of cellulose. Similarly, figure 3 lists cellulose among the other substrates which were actually directly converted, even though this was done following hydrogenolysis at high T/high p under H_2. This must be clearly indicated in the figure and it should not be compared directly in the same graph.

In addition, the reactions were performed using UV LEDs, yet the authors mention storage of solar energy and show the sun driving the process in figure 1. This is misleading, as it implies the process is driven by actual or simulated sunlight, which contains very little UV light. The use of UV light needs to be explicitly stated in the abstract and introduction, and the figure needs to be changed. In addition, there is no description of the light intensity incident on the sample (the LED wattage is insufficient detail). What was the quantum efficiency of the process?

The activity of the system should be given in a unit that is more easily compared with other studies, e.g. mmol/g/h since L depends on temperature and pressure. There should also be a better comparison with other catalysts from the literature with similar reactivity, ideally in the form of a table.

What exactly is the role of the organic solvent? Can the authors rule out any degradation of the solvent, e.g. through ^{13}C labelling? The authors claim this is a result of fewer OH radical being generated, however mechanistic studies to support this claim are missing (e.g. quantifying OH radicals). In general, it has been widely shown that photooxidation of polyols on TiO_2 proceeds preferentially through direct hole transfer to substrate bound to the surface, in particular for polyols (see for example J. Phys. Chem. C 2011, 115, 4642-4648, J. Catal., 2016, 344, 806-816; Chem. Rev. 2014, 114, 9919-9986). This is also in line with the calculations performed by the authors, yet they still claim that OH radicals are involved. Please comment and add experimental data. Can the authors rule out that generated CO_2 is being reduced to CO in the cases with high Cu loading? Especially in organic solvents, CO_2 is highly soluble and Cu is a known catalyst for CO_2 reduction. This could be confirmed/ruled out with ^{13}C labelling experiments.

There is also something wrong with the mechanism shown in figure 3f: The dehydration of formic acid is not an oxidation reaction (it is redox-neutral), yet the figure shows conversion of formic acid to CO by a hole in the TiO_2 VB coupled to proton reduction at the CB. Where do the electrons for HER come from? The authors show compelling evidence for CuO_x acting as hole traps. This would remove holes from TiO_2 onto CuO_x, which is known to oxidise formic acid to CO_2. So why is addition of Cu suppressing the oxidation to CO_2? And why do the authors state that "oxidation of formic acid mainly takes place on TiO_2 phase", when all the holes are supposedly located on CuO_x? This doesn't quite add up.

How exactly was the product yield calculated? Looking at figure 2b and d, the total yield seems to be >100%. Please explain. On what reaction equation was this yield based?

Further comments:
• The language needs some attention (wording, grammar)
• Fig. 3: A GC trace is not a spectrum. Please reword
• Fig. S7 should also include the CO/CO_2 ratio in the absence of any Cu
• There are some graphs in the supporting information that are not being referred to in the manuscript (e.g. S13). Please either refer to them or remove them from the supporting information. What is the purpose of the supporting video? I struggle a bit to see the point. It would be more interesting to see H_2 evolution during irradiation.

Reviewer #3 (Remarks to the Author):

This manuscript by Wang et al describes the photoreforming of biomass-derived molecules into methanol and Syngas, thereby creating useful feedstocks from an abundant resource. This is undertaken using TiO_2 nanorods loaded with Cu, which work efficiently when irradiated with high-intensity UV light. The observed activity is impressive compared to other photocatalysts for the reaction, however the discoveries do not represent the significant advance in knowledge required for publication in Nature Communications. The system relies predominantly on well-studied components and reactions and the report focuses on their optimisation towards high activity and selectivity. I therefore believe the data would be best suited to a more specialist journal.

Furthermore, although their data has been well-presented and discussed, there remain a number of issues that must be addressed before publishing:

1) What power of light is hitting the sample during the experiment? It appears that the intensity is high and only UV-light is implemented. How does the system work under simulated solar light?

2) The mechanism involving a semiconducting CuO_x on the surface of TiO_2 is not sufficiently justified. If such a tandem light absorbing mechanism was occurring it should be visible in the UV-visible spectrum of the photocatalyst.

3) In Figure 3a what solvent conditions were used when varying the Cu quantity?

4) Hydroxyl radicals are often referred to in order to explain the oxidation mechanism on the TiO_2, however these statements are not referenced. Since there is some controversy concerning the presence of such radicals, these statements must be justified.

5) Could the Cu be doing CO_2 reduction to produce CO? Cu is a well-known CO_2 reduction catalyst

6) A lot of contemporary references on biomass oxidation are missing (see

doi.org/10.1016/j.ccr.2015.12.009 and doi: 10.1002/anie.201710133 for examples).

7) A lot of discussion has been added to the supplementary information - why are the authors publishing this work as a communication when they could write a detailed full paper?

In summary the study is well carried out but the new hypotheses are not sufficiently justified and the catalyst not sufficiently novel for publication in Nature Communications. In terms of readability the English should be improved before publication, as the syntax is often irregular. I recommend the authors strengthen their mechanistic studies with further experimental evidence and submit this work to a more specialised journal.

Response to the reviewers

Reviewer: 1

This manuscript reported a facile way for the conversion of biomass-derived polyols, sugars, cellulose and even raw wood sawdust with the Cu/TNT as photocatalyst into methanol and syngas ($CO+H_2$) via photo-splitting at room temperature. The results showed that Cu dispersed on titanium oxide nanorods (TNR) rich in defects were effective for the selective C−C bond cleavage to methanol. High yield of methanol and co-production of syngas in the gas phase was obtained via controlling the energy band structure of Cu/TNR. The work is interesting although with few ambiguous claims. It is recommended for publication in Nature Communication after major revision. Specific comments are as follows.

Question 1. What's the role of acetonitrile in this reaction? The yields of H_2 and methanol were changed with the variation of solvent according to the data in the manuscript. What about the products with the water only as the solvent?

Response: The solvent effect is complex. As reported in the literature about the photocatalytic oxidation of alcohols, the alcohols are converted into target products in high selectivity (J. Catal. 2009, 266, 279-285; Angew. Chem. Int. Ed. 2008, 47, 9730.) in organic solvent. When the reactions are conducted in water, the alcohols are usually degradation to CO_2, resulting in low selectivity to the target products (J. Am. Chem. Soc. 2008, 130, 1568; Chem. 2011, 17, 9816; Green Chem. 2009, 11, 510). In photocatalytic oxidations, the initial oxidation steps may proceed by two different routes. The holes oxidize water to hydroxyl radicals, which further serve as the active species for the oxidation of organic species. The other route is the direct oxidation of the chemisorbed or strongly adsorbed organic species by holes on the surface of the photocatalyst. The two routes are both possible in the photoreforming of polyols in the present of water. In water solvent, hydroxyl radicals are involved. The oxidation of organic species by hydroxyl radicals leads to the formation of carboxylic acids which decompose to CO_2 via decarboxylation (Appl. Catal. B: Environ. 2011, 106, 681). We think the hydroxyl radicals are the major cause for over oxidation of the organic species to CO_2. Therefore, the competitive oxidation of water and polyols by the holes affect the final product in the water-organic solvent system. We have detected the hydroxyl radical using Electron paramagnetic resonance (EPR). 5,5-Dimethyl-1-pyrroline N-oxide (DMPO) was used to capture the hydroxyl radicals. In water solvent, the signals of hydroxyl radicals were detected (Figure R1). When decreasing the water content to 5%, no obvious signals of hydroxyl radicals were observed. It can be deduced that decreasing the water concentration in the water-organic solvent system will reduce the hydroxyl radicals, thus slows down the degradation of the organic species to CO_2, which is probably the one of its effect of organic solvent. With water only as the solvent, the major product is the CO_2 and methanol was also detected (Figure S2). The yield of methanol is much lower than

that in MeCN-H_2O (8:2) solvent.

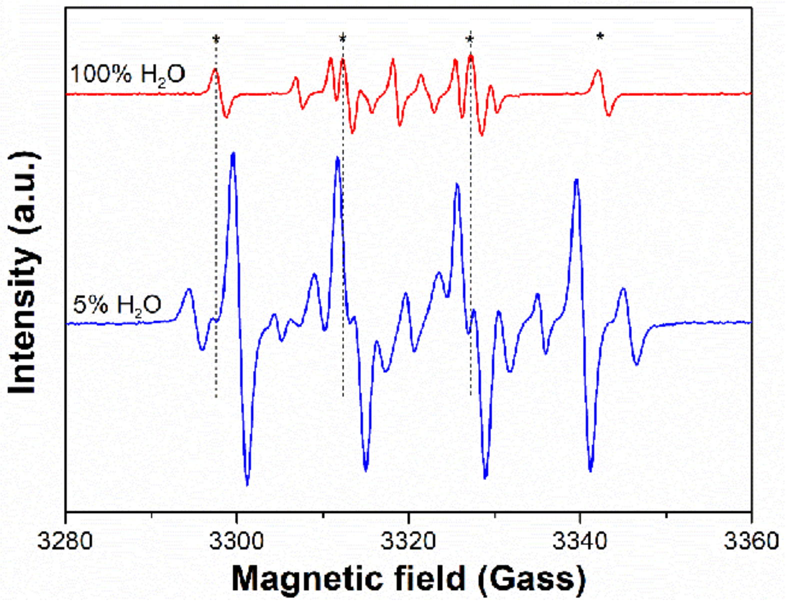

Figure R1 EPR spectra of hydroxyl radical (*) produced by photo irradiation over 2Cu/TNR and stabilized by 5,5-dimethyl-1-pyrroline N-oxide (DMPO).

Question 2. As the authors claimed that "The gas product distribution is dominantly controlled by the decomposition way of formic acid.", the authors should pay close attention to the yield of formic acid in the solution. What's the yield of formic acid in the solution? Is it associated with the amount of Cu on the TNT?

Response: We have provided the yield of formic acid in Table S2. When 2Cu/TNR was used in MeCN: water (8:2) solution, as the reaction proceeded, the yield of formic acid is below 5%. There is not obvious accumulation of formic acid because the decomposition of formic acid is faster than the generation of formic acid from glycerol. When decreasing the copper content, the decomposition of formic acid slowed down, higher yield of formic acid was obtained over 0.1Cu/TNR (Figure R2a). This is because the decomposition of formic acid is slower over 0.1Cu/TNR (Figure R2b).

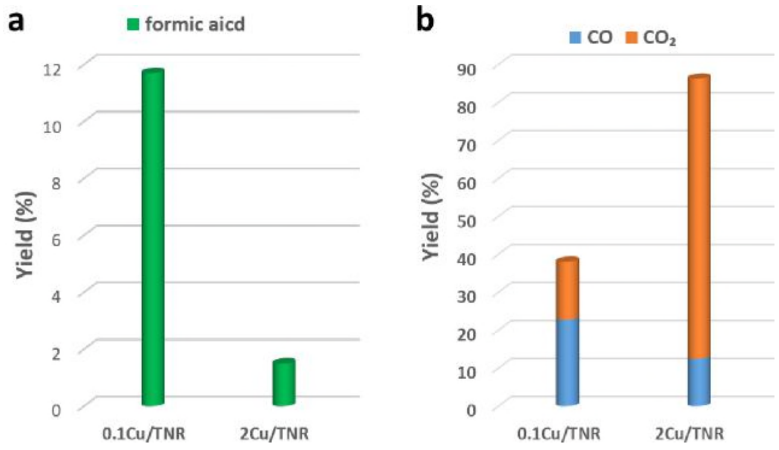

Figure R2. (a) The yield of formic acid in the photoreforming of glycerol over 0.1Cu/TNR and 2Cu/TNR. Reaction conditions: 10 mg of glycerol, 10 mg of catalyst, 0.8 mL of MeCN, 0.2 mL of water, 18 W 365 nm LED irradiation for 48 h. (b) The decomposition of formic acid over 0.1Cu/TNR and 2Cu/TNR. Reaction conditions: 10 mg of formic acid, 10 mg catalyst, 0.95 mL of MeCN, 0.05 mL of water, 365 nm LED (18 W, 55 mv cm^{-2}) irradiation for 12 h.

Question 3. The FTIR information of formic acid absorbed on the Cu/TNT samples should be supplied for clarifying the mechanism of dehydrogenation and dehydration of formic acid.

Response: We conducted the FTIR characterization of formic acid adsorption on Cu/TNR samples (Figure R3). With low copper loading, the spectra of formic acid adsorption on 0.1Cu/TNR is similar to the formic acid adsorption on TiO_2 (J. Phys. Chem. B 2001, 105, 7678). The bands at 1360 and 1581 cm^{-1} are assigned to -COO- symmetric and antisymmetric stretches, 1378 cm^{-1} is signed to C-H deformation. With increasing the copper loading, the bands changed. Besides the shifting of the -COO- symmetric and antisymmetric stretches, new bands at 1650 and 1609 cm^{-1} appear which are probably due to the adsorption of formic acid on CuOx. We have discussed this result in the revised manuscript.

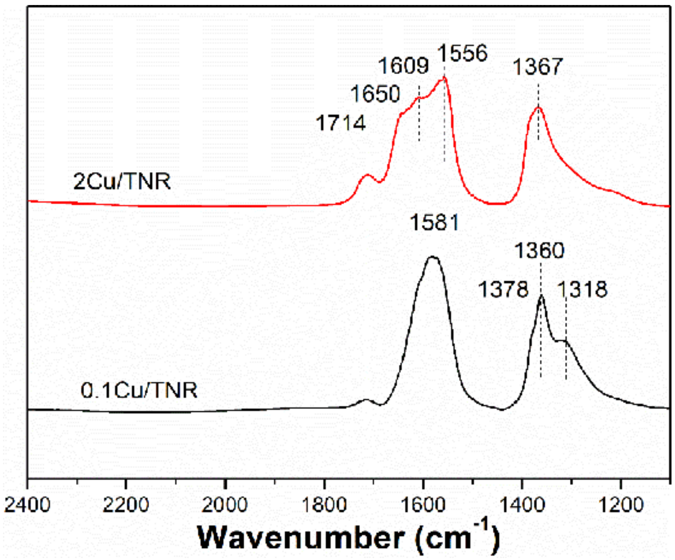

Figure R3. Formic acid adsorption on Cu/TNR.

Reviewer: 2

In their manuscript, Wang et al. report on the photocatalytic reforming of biomass-derived compounds to methanol and syngas. There is a growing interest in generating well-defined organic feedstock chemicals from biomass under mild conditions. Currently, there are only a handful of known examples for such a process, and most of these operate under rather harsh conditions and produce mostly hydrogen. The results presented here are exciting, as they show efficient production of methanol at near-ambient conditions using a simple photocatalyst based on inexpensive Cu-doped TiO_2. While the performance of this system is promising, there are a number of issues that need to be addressed before publication can be considered.

Question 1. The work on "real" biomass, rather than refined biomass-derived compounds deserves some discussion here. The authors claim that their work was superior to other processes for biomass utilisation, because of the lower temperatures and pressures required. However, in order to be able to convert true biomass with their system, a pre-treatment at 240 °C and 40 bars hydrogen is needed, which is not much lower than the 300 °C deemed too high in the introduction. Please comment on what the actual advantage of the present work is. The statement in the conclusion that "this process requires neither high pressure hydrogen nor high temperature" is clearly wrong and should be removed. The authors alternatively use concentrated sulphuric acid, which subsequently must be removed with stoichiometric amounts of $Ba(OH)_2$, which clearly contradicts the claim of being more atom-efficient or milder than other processes.

Response: Thanks for your comments. For the direct production of methanol from biomass, very few works have reported. Tsang et al. reported the hydrogenolysis of ethylene glycol (EG) to methanol over Pd/Fe_2O_3 with only 4.3% yield. Hutchings et al. realized the H_2-free production of methanol from glycerol reforming at about 300 ^oC. These works are thermal driven reactions and use the C2 and C3 polyols as the substrate. In the present work, we reported a light driven process to obtain methanol and syngas from biomass resource. The biomass resource includes C2-C6 polyols and sugar and were operated at room temperature. The glycerol comes from fats. C5-C6 polyols and sugars were generated from hemicellulose and cellulose which are the most abundant biomass resources. Although the present method can not direct convert cellulose, our method can use the hemicellulose and cellulose derived C5-C6 polyols and sugars which will hopefully expand the biomass resource from fats to hemicellulose and cellulose.

The conversion of cellulose and wood sawdust into C6 polyols and sugars have been commercialized. In this work, we showed an example for the conversion of cellulose and wood sawdust via a two-step process. Indeed, the pretreatment involves high temperature or concentrated sulphuric acid to generate polyols or sugars which was then subjected to a photo-reforming reaction at room temperature.

We are also aware that the description in the introduction and conclusion may be not clear and appropriate. We have modified the introduction and conclusion accordingly, which are highlighted in the revised manuscript. The pretreatment of cellulose and wood sawdust is not a photo reaction. We also revised the title and delete “cellulose” from the title in order to make it correct.

Question 2. The title claiming “photo splitting of ... cellulose” is misleading, since the cellulose is never directly exposed to photocatalysis, but the sugars generated from conventional processing of cellulose. Similarly, figure 3 lists cellulose among the other substrates which were actually directly converted, even though this was done following hydrogenolysis at high T/high p under H_2. This must be clearly indicated in the figure and it should not be compared directly in the same graph.

Response: We accepted the reviewer’s suggestion. We delete the “cellulose” from the title. The data of cellulose conversion was removed from the Figure 2 and Figure 3. These datas were presented in Figure 4a.

Question 3. In addition, the reactions were performed using UV LEDs, yet the authors mention storage of solar energy and show the sun driving the process in figure 1. This is misleading, as it implies the process is driven by actual or simulated sunlight, which contains very little UV light. The use of UV light needs to be explicitly stated in the abstract and introduction, and the figure needs to be changed. In addition, there is no description of the light intensity incident on the sample (the LED wattage is insufficient detail).

Response: We accepted the reviewer's suggestion. We have modified figure 1 and have stated UV light in abstract and introduction. The light intensity is 55 mw/cm^{-2} which is provided in the reaction conditions.

Question 4. What was the quantum efficiency of the process?

Response: The apparent quantum yield (AQY) was measured over 2Cu/TNR in MeCN-H_2O (8:2) solution with UV LEDs light (input power of 50 W, 365 nm) by top irradiation. The apparent quantum yield was calculated based on the following equation.

$$AQY = \frac{\text{electrons consumed by the products}}{\text{Photons}} \times 100$$

$$= \frac{4\,\underline{3}\ t\,h\,n \times + \underline{3}\ 1 \times + {}^{2}h\,n \times}{\times\ t \times} \times 100 = 3.4\%$$

where $n_{methanol}$, $n_{methanol}$, and $n_{hydrogen}$ are the molar amount of methanol, EG and hydrogen, respectively, and N_A, I, t and S represent Avogadro's constant, light intensity, reaction time and irradiation area, respectively.

Question 5. The activity of the system should be given in a unit that is more easily compared with other studies, e.g. mmol/g/h since L depends on temperature and pressure. There should also be a better comparison with other catalysts from the literature with similar reactivity, ideally in the form of a table.

Response: We accepted the reviewer's suggestion. We summarized the works on the photoreforming of glycerol which is provided in the supplementary materials (Table S1). All the reported catalysts dominantly generate CO_2 as the major products.

Photocatalyst	Reaction medium	Light Source	P/W (I/mW cm^{-2})	Atmosphere	T/□	Production rate/μmol$g_{cat}^{-1}h^{-1}$					
						H_2	CH_4	CO	CO_2	CH_3OH	
0.5 wt% Pt/TiO_2(P25)	Glycerol (aq, 0.368mM)	Xe-arc lamp	450	Ar	40	89.9	-	-	36.7	-	Reference
0.5 wt% Pt/TiO_2(P25)	Glycerol (aq, 17.7mM)	Xe-arc lamp	450	Ar	40	1151	-	-	300	-	17
TiO_2(P25)	Glycerol (aq, 20mM)	Xe-arc lamp	450	O_2	40	-	-	-	975	-	18
TiO_2(P25)	Glycerol (aq, 20mM)	Xe-arc lamp	450	Ar	40	75	-	-	<7.5	1.25	18
0.5 wt% Pt/TiO_2(P25)	Glycerol (aq, 20mM)	Xe-arc lamp	450	O_2	40	-	-	-	2250	-	18
0.5 wt% Pt/TiO_2(P25)	Glycerol (aq, 20mM)	Xe-arc lamp	450	Ar	40	1875	-	-	450	0.42	18
1 wt% Pt/TiO_2(P25)	H_2O/Glycerol (*l* , 30:1)	Xe-arc lamp	300	Vacuum	10	4280	-	-	-	-	19
2 wt% Pt/TiO_2(P25)	Glycerol (aq, 8.3mM)	High-pressure Hg lamp	-	N_2	20	4285	-	-	1836	-	20
0.5 wt% Pd/TiO_2(P25)	H_2O/Glycerol (*l* , 1000:1)	Xe-arc lamp	400	Ar	-	892.9	-	-	-	-	21
0.5 wt% Pd/TiO_2(P25)	H_2O/Glycerol (*l,* 1000:1)	Xe-arc lamp	400	Ar	-	1075	-	-	-	-	22
1 wt% Pd/TiO_2(P25)	25% Glycerol (H_2O)	UV LED (365nm)	-	-	-	9846	-	-	-	-	23
0.5 wt% Au/TiO_2(P25)	10% Glycerol	UV Light (365nm)	100	N_2		27900	-	-	-	-	24
2 wt% Au/TiO_2(P25)	H_2O/Glycerol (*l,* 1000:1)	Xe-arc lamp	400	Ar	-	639.9	-	-	-	-	22
1 wt% Au_3Pd/TiO_2(P25)	25% Glycerol (aq)	UV LED (365nm)	(60)	Ar	-	13231	-	-	-	-	23
1 wt% Pd_3Au/TiO_2(P25)	25% Glycerol (aq)	UV LED (365nm)	(60)	Ar	-	14769	-	-	-	-	23
1 wt%$Au_{shell}Pd_{core}$/TiO_2(P^{2}5)	25% Glycerol (aq)	UV LED (365nm)	(60)	Ar	-	14923	-	-	-	-	23
1 wt% $Pd_{shell}Au_{core}$/TiO_2(P25)	25% Glycerol (aq)	UV LED (365nm)	(60)	Ar	-	19631	-	-	-	-	23
1 wt% $Pd_{shell}Au_{core}$/TiO_2(P^{2}5)	25% Crude Glycerol (aq)	UV LED (365nm)	(60)	Ar	-	10109	-	-	-	-	23
0.4 wt% CuO/TiO_2(P25)	Glycerol (aq, 1M)	Xe-arc lamp	250	Ar	-	863	-	-	50	-	25
1.25 wt% CuO/TiO_2(P25)	Crude Glycerol (aq, 1.04wt%)	UVA (340nm)	-	Ar	40	92	-	-	-	-	26
1.3 wt% CuO/TiO_2(P25)	Glycerol (aq, 0.1M)	UV LED (365nm)	4×3	N_2	-	2061	-	-	-	-	27
Cu_2O/TiO_2(P25)	Glycerol (aq, 0.1M)	Xe-arc lamp (λ>420nm)	500	-	-	240	-	-	-	-	28
0.25 wt% Sn/0.68 wt% RuO_2/TiO_2(P25)	H_2O/Glycerol (*l,* 33:1)	High-pressure Hg lamp	125	N_2	20	31500	140	7300	-	-	29
TiO_2(nanorod)	5 vol% Glycerol (aq)	Sunlight	-	N_2	-	2950	-	-	-	-	30
0.1 wt% Pt/TiO_2(74% Rutile)	Glycerol (aq, 1.3mM)	Xe-arc lamp	300	-	-	7784	-	-	-	-	31
0.5 wt% Pt/TiO_2(porous)	H_2O/Glycerol (*l,* 5:1)	High-pressure Hg lamp	500	N_2	30	6250	-	-	-	-	32
1 wt% Pt/TiO_2	Glycerol (aq, 2.7mM)	High-pressure Hg lamp	125	N_2	30	6000	18	-	2500	-	33
2 wt% Pt/TiO_2	Glycerol (aq, 0.61g/L)	High-pressure Hg lamp	-	N_2	20	8184.5	-	-	2232.1	-	34
2.1 wt% Pt/TiO_2	Glycerol (aq, 7.34M)	UV (300-400nm)	12×15	He		6335	-	-	72	-	35
4 wt% Pt/TiO_2	H_2O/Glycerol (*l* , 1:1)	Xe-arc lamp (λ>320nm)	500	-	40	2500	-	-	-	-	36
0.5 wt% Au/TiO_2(Anatase)	10% Glycerol	UV Light (365nm)	100	N_2	40	15000	-	-	-	-	24
0.5 wt% Au/TiO_2(brookite)	10% Glycerol	UV Light (365nm)	100	N_2	40	13800	-	-	-	-	24
0.5 wt% Au/TiO_2(Rutile)	10% Glycerol	UV Light (365nm)	100	N_2	40	3200	-	-	-	-	24
1 wt% Au/TiO_2(Anatase)	H_2O/Glycerol (*l* , 1:1)	Xe-arc lamp	300	Ar	25	4900	-	-	-	-	37
1 wt% Au/TiO_2/cordierite honeycomb	H_2O/Glycerol (*l* , 1:1 mol)	UV LED (365±5nm)	4×12	Ar	25	768	-	-	-	-	38
1 wt% Au/TiO_2/cordierite honeycomb	H_2O/Bio-Glycerol (*l* , 1:1 mol)	UV LED (365±5nm)	4×12	Ar	25	300	-	-	-	-	38
2 wt% Pt/F-TiO_2(nanosheet)	Glycerol (aq, 0.1M)	Xe-arc lamp	350	N_2	-	8455	-	-	-	-	39
Pt/B-TiO_2	5% Glycerol (aq)	Xe-arc lamp	300	N_2	-	4133	-	-	-	-	40
Pt/N-TiO_2	5% Glycerol (aq)	Xe-arc lamp	300	N_2	-	5133	1.13	18.1	496.6	0.9	40
Pt/(B, N)-TiO_2	5% Glycerol (aq)	Xe-arc lamp	300	N_2	-	8200	0.92	1.85	1040	1.04	40
0.3 wt% Pt/1 mol% Gd-TiO_2	H_2O/Glycerol (*l* , 50:1)	Hg lamp	300	Ar	77~82	12075	-	-	-	-	41
10 mol% Cu/TiO_2	10 vol% Glycerol (aq) + 2M NaOH	Halogen	500	Ar	24	5772	-	-	-	-	42
1wt% Cu_2O/TiO_2	5% Glycerol (aq)	Hg lamp	250	N_2	-	16656	-	-	-	-	43
1.5 wt% Cu_2O/TiO_2(nanorod)	5 vol% Glycerol (aq)	Sunlight	-	N_2	-	50339	-	-	-	-	30
1wt% CuO_x/TiO_2	Glycerol (aq, 1M)	Xe-arc lamp	150	Ar	-	580	-	-	-	-	44
1wt% CuO_x/TiO_2	Glycerol (aq, 1M)	High-pressure Hg lamp	125	Ar	-	1200	-	-	200	-	44
2.5wt% CuO_x/TiO_2	Glycerol (aq, 1M)	Hg lamp	125	Ar	20	970	-	-	-	-	45
1.5 wt% CuO/TiO_2(nanotube)	5 vol% Glycerol (aq)	Sunlight	-	N_2	-	99823	-	-	-	-	46
2 wt% CuO/TiO_2(Anatase/Rutile=7:3)	5% Glycerol (aq)	High-pressure Hg lamp	500	N_2	50	1370	300	140	70	-	47
3wt% RGO-1wt% Cu_2O/TiO_2	5% Glycerol (aq)	Hg lamp	250	N_2	-	110968	-	-	-	-	43
2 wt% NiO/TiO_2(Anatase/Rutile=7:3)	5% Glycerol (aq)	High-pressure Hg lamp	500	N_2	50	1230	19	106	41	-	47
10 wt% NiO/TiO_2	H_2O/Glycerol (*l* , 5:1)	High-pressure Hg lamp	500	N_2	50	900	-	130	590	-	48
2 wt% CoO/TiO_2(Anatase/Rutile=7:3)	5% Glycerol (aq)	High-pressure Hg lamp	500	N_2	50	660	60	100	50	-	47
NiO	H_2O/Glycerol (*l* , 5:1)	High-pressure Hg lamp	500	N_2	50	91	-	2124	917	-	48
3wt% RGO/TiO_2	5% Glycerol (aq)	Hg lamp	250	N_2	-	8226	-	-	-	-	43
ZnO(Nanotube arrays)	10 vol% Glycerol (aq)	Xe-arc lamp	350	-	25	38	-	-	-	-	49
RGO/ZnO	5% Glycerol (aq)	Xe-arc lamp	300	Ar	RT	82	-	-	22	-	50
12wt% RGO/ZnO	5% Glycerol (aq)	Xe-arc lamp	300	Ar	RT	92	-	-	-	-	51
ZnS	10 vol% Glycerol (aq)	Xe-arc lamp	350	-	25	232	-	-	-	-	49
ZnS/ZnO(Nanotube arrays)	10 vol% Glycerol (aq)	Xe-arc lamp	350	-	25	384	-	-	-	-	49
ZnS/ZnO(Nanorod)	7% Glycerol (aq)	High-pressure Hg lamp	125	Ar	-	2609	-	-	-	-	52
ZnS/ZnO(Nanorod)	7% Glycerol (aq)	Xe-arc lamp	500	Ar	-	388	-	-	-	-	52
ZnO@Bi_2S_3/ZnS/RGO	5% Glycerol (aq)	Xe-arc lamp	300	Ar	RT	310	-	-	80	-	50
0.5 wt% Pt/3 wt% Au-WO_3	Glycerol (aq, 2mM)	Xe-arc lamp (450-600nm)	(83)	Ar	25	132	-	-	56	-	53
Bi_2WO_6	H_2O/Glycerol (*l* , 1:1)	Xe-arc lamp	300	Ar	-	7400	-	-	-	-	54
0.2% wt Pt/CdS	10 vol% Glycerol (aq)	Xe-arc lamp (>420nm)	300	Vacuum	10±5	170	-	-	-	-	55
0.2% wt MoS_2/CdS	10 vol% Glycerol (aq)	Xe-arc lamp (>420nm)	300	Vacuum	10±5	370	-	-	-	-	55
0.5wt% $Cd_{0.5}Zn_{0.5}S$	1.368M Glycerol (aq) + 1M NaOH	High-pressure Hg lamp (>420nm)	250	N_2	-	630	-	-	-	-	56
$Cd_{0.5}Zn_{0.5}S$: (γ-Zn$(OH)_2$)	H_2O/Glycerol (*l* , 1:1) + 1.5M NaCl	Hg-Xe arc lamp (418~IR)	500	Ar	-	239	-	-	-	-	57
2wt%Cu/TNR	Glycerol (MeCN-H_2O, 8:2)	UV LED (365nm)	(55)	Ar	RT	1308	-	47	523	990	This work
0.1wt%Cu/TNR	Glycerol (MeCN-H_2O, 9.5:0.5)	UV LED (365nm)	(55)	Ar	RT	36	-	83	12	51	This work

Question 6. What exactly is the role of the organic solvent? Can the authors rule out any degradation of the solvent, e.g. through ^{13}C labelling? The authors claim this is a result of fewer OH radical being generated, however mechanistic studies to support this claim are missing (e.g. quantifying OH radicals). In general, it has been widely shown that photooxidation of polyols on TiO_2 proceeds preferentially through direct hole transfer to substrate bound to the surface, in particular for polyols (see for example J. Phys. Chem. C 2011, 115, 4642-4648, J. Catal., 2016, 344, 806-816; Chem. Rev. 2014, 114, 9919-9986). This is also in line with the calculations performed by the authors, yet they still claim that OH radicals are involved. Please comment and add experimental data.

Response: In photoreforming of polyols, the initial oxidation steps may proceed by two different routes. The holes oxidize water to hydroxyl radicals, which serve as the

active species for the oxidation of organic species. The other route is the direct oxidation of the chemisorbed or strongly adsorbed organic species by holes on the surface of the photocatalyst. There is some controversy concerning the two routes. Alcohols and water are known to adsorb competitively on the oxide surface (J. Phys. Chem. C 2007, 111, 22, 8005). It has also been demonstrated that direct oxidation of the adsorbed organic species by the holes is the dominant way for the TiO_2 based catalyst in the absence of water, and upon increasing water content, indirect oxidation by hydroxyl radicals would be predominant (J. Catal., 2011, 280, 168). The hydroxyl radicals have been captured by EPR upon photoirradiation of aqueous suspension of TiO_2 (Chem. Lett., 1985, 14,1799-1802). Therefore, the two routes are both possible in the photoreforming of polyols in the presence of water.

The solvent effect is complex. As reported in the literature about the photocatalytic oxidation of alcohols, the alcohols are converted into target products in high selectivity in organic solvent (J. Catal. 2009, 266, 279; Angew. Chem. Int. Ed. 2008, 47, 9730.). When the reaction is conducted in water solvent, the alcohols are usually degraded to CO_2, resulting in low selectivity to the target products (J. Am. Chem. Soc. 2008, 130, 1568; Chem. 2011, 17, 9816. Green Chem. 2009, 11, 510.). According to the literature, polyols on TiO_2 proceeds preferentially through direct hole transfer to substrate bound to the surface, but the presence of hydroxyl radicals can not be eliminated. In water solvent, hydroxyl radicals are involved. The oxidation of organic species by hydroxyl radicals leads to the formation of carboxylic acids which decomposes to CO_2 via decarboxylation (Appl. Catal. B: Environ. 2011, 106, 681). We think the hydroxyl radicals are the major cause for over oxidation of the organic species to CO_2. The competitive oxidation of water and polyols by the holes affect the final product in the water-organic solvent system. We have detected the hydroxyl radical using EPR, and 5,5-dimethyl-1-pyrroline N-oxide (DMPO) was used to capture the hydroxyl radicals (Figure R1). In water solvent, the signals of hydroxyl radicals were detected. When decreasing the water content to 5%, no obvious signals of hydroxyl radicals were observed. It can be deduced that decreasing the water concentration in the water-organic solvent system will reduce the hydroxyl radicals, thus slows down the degradation of the organic species to CO_2, which is probably one of the effect of organic solvent.

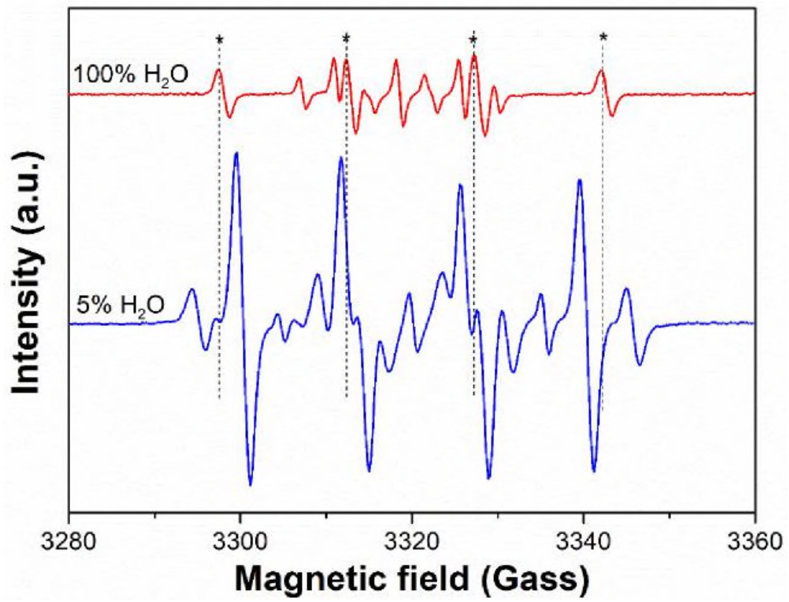

Figure R1 EPR spectra of hydroxyl radical (*) produced by photo irradiation over 2Cu/TNR and stabilized by 5,5-dimethyl-1-pyrroline N-oxide (DMPO).

We performed the photoreaction in the absence of glycerol. In the liquid phase, no obvious acetonitrile degradation products were observed (Figure R4a). In the gas phase, only a very weak CO_2 peak was observed (Figure R4b). These results demonstrate that the acetonitrile is relatively stable under the reaction conditions.

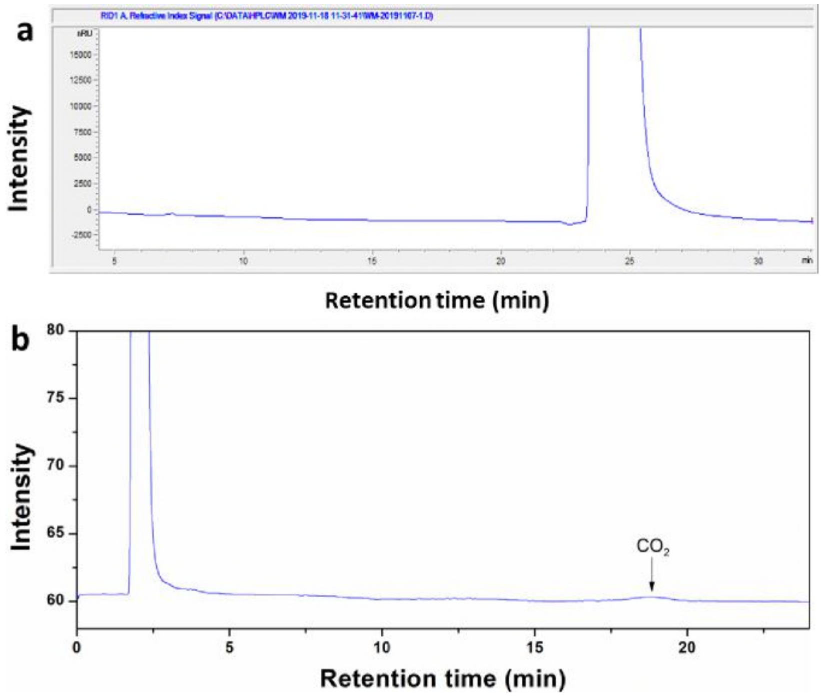

Figure R4 The HPLC (a) and GC (b) curves. Reaction conditions: 0.8 mL of MeCN, 0.2 mL of water, 365 nm LED (18 W, 55 mv cm^{-2}), Ar, 12 h

Question 7. Can the authors rule out that generated CO_2 is being reduced to CO in the cases with high Cu loading? Especially in organic solvents, CO_2 is highly soluble and Cu is a known catalyst for CO_2 reduction. This could be confirmed/ruled out with 13C labelling experiments.

Response: We used ^{13}C labelling experiments to study the source of CO. The photoreforming of glycerol was carried out in the presence of $^{13}CO_2$. $^{13}CO_2$ (1 mL) was injected in the reactor before reaction. Two catalysts with high copper loading (2Cu/TNR) and low copper loading (0.1 Cu/TNR) were studied. After reaction, the gas phase was analyzed by mass spectroscopy. Only minor amount of ^{13}CO was generated. The results demonstrated that CO can be both derived from the decomposition of the intermediates in the liquid phase and the reduction of CO_2 in the gas phase, and the former is the dominant way for the generation of CO from glycerol.

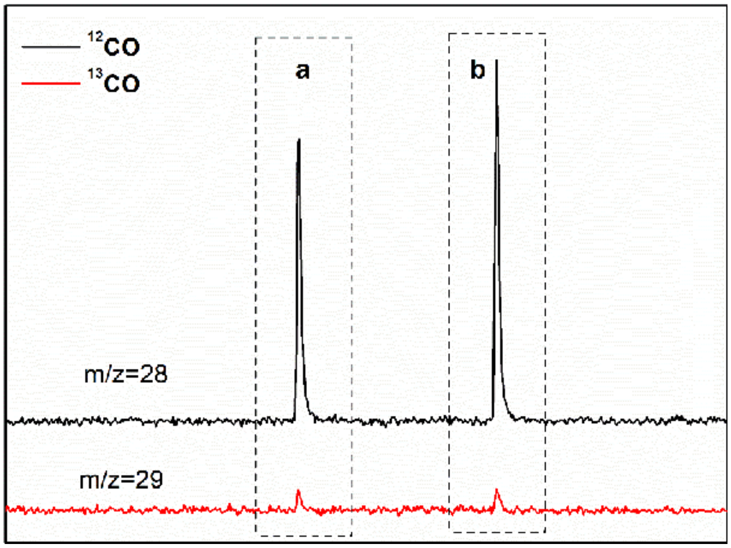

Figure R5 The mass spectroscopy of selected m/z=28, 29. Reaction conditions: (a) 10 mg of 2Cu/TNR, 10 mg of glycerol, 0.8 mL of MeCN, 0.2 mL of water, 365 nm LED (18 W, 55 mv cm^{-2}), 1mL $^{13}CO_2$, Ar atmosphere, 12 h; (b)10 mg of 0.1Cu/TNR, 10 mg of glycerol, 0.95 mL of MeCN, 0.05 mL of water, 365 nm LED (18 W, 55 mv cm^{-2}), 1mL $^{13}CO_2$, Ar atmosphere, 24 h.

Question 8. There is also something wrong with the mechanism shown in figure 3f: The dehydration of formic acid is not an oxidation reaction (it is redox-neutral), yet the figure shows conversion of formic acid to CO by a hole in the TiO_2 VB coupled to proton reduction at the CB. Where do the electrons for HER come from? The authors show compelling evidence for CuOx acting as hole traps. This would remove holes from TiO_2 onto CuOx, which is known to oxidise formic acid to CO_2. So why is addition of Cu suppressing the oxidation to CO_2? And why do the authors state that "oxidation of formic acid mainly takes place on TiO_2 phase", when all the holes are supposedly located on CuOx? This doesn't quite add up.

Response: Thanks for pointing out this mistake. Indeed, the dehydration of formic

acid is a redox-neutral reaction. The figure for the formic acid dehydration is not correct and misleading. We have modified this figure and accordingly the discussions.

From the literature and our experiment results, copper phase prefers to follow dehydrogenation reaction to afford CO_2, while TiO_2 phase favors the dehydration reaction to generate CO. For the Cu/TNR with high copper loading amount (2Cu/TNR), CuO_x nanoparticles are dominant and a CuO_x-TiO_2 heterojunction is formed. Upon photoexcitation, the photoexcited holes in the valance band (VB) of TiO_2 transfer to VB of CuO_x. Therefore, the oxidation of formic acid by holes takes place on the CuO_x phase, and formic acid is decomposed to CO_2 and H_2 via dehydrogenation reaction. For the Cu/TNR with low copper loading amount (0.1Cu/TNR), instead of CuO_x particles, doped Cu^{2+} are dominant, forming a defect level in the bandgap. The photoexcited holes remain in the TiO_2. It is reported that the oxidation of water by holes on TiO_2 generates acid which is permanent under photoirradiation and distributed close to the surface (Angew. Chem. Int. Ed. 2016, 55, 13001). Because there is nearly no CuO_x particles in 0.1Cu/TNR, the formic acid dominantly adsorbed on TiO_2 phase. For 0.1Cu/TNR, the holes on TiO_2 oxidize water to in situ generate acidic TiO_2 surface under photoirradiation, which may promote the dehydration of formic acid to CO and H_2O (Figure R6).

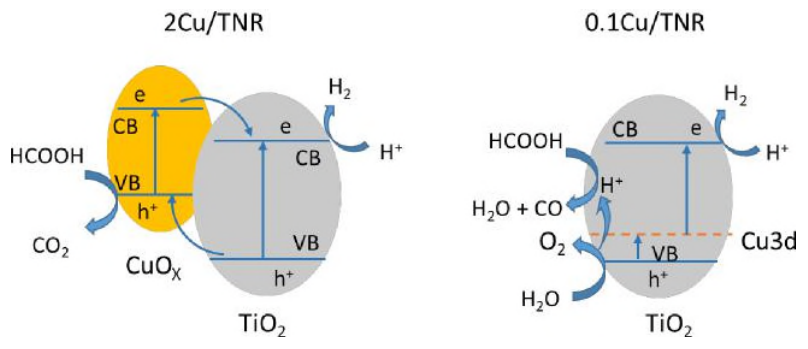

Figure R6 The proposed mechanism for formic acid decomposition.

Question 9. How exactly was the product yield calculated? Looking at figure 2b and d, the total yield seems to be >100%. Please explain. On what reaction equation was this yield based?

Response:

The yields are calculated based on sum of total carbon in the products.

$Y_p = (N_p \times C_p)/(N_{substrate} \times C_{substrate}) \times 100$, where Np is the molar amount of the product, Cp is the number of carbon in the product, $N_{substrate}$ is the molar amount of substrate, and $C_{substrate}$ is the number of carbon in the substrate.

The molar of gas products is calculated based on the gas equation.

$$N = PV/RT = 101.3 \times V/(8.314 \times T)$$

The volume of gas is depended on the temperature. Previously, we used 273 K to

calculate the molar of gas, which is the reason that makes the total carbon higher than 100%. We recalculated the molar of gas product at room temperature (298K). We also repeated the experiment data. The carbon balance was provided in Table S2-S6. We provided the calculation method in the supplementary materials.

Question 10. The language needs some attention (wording, grammar)

- Fig. 3: A GC trace is not a spectrum. Please reword

Response: We have changed the "spectrum" to "curve".

Question 11. Fig. S7 should also include the CO/CO_2 ratio in the absence of any Cu
Response: We have added this data in the revised supplementary materials.

Question 12. There are some graphs in the supporting information that are not being referred to in the manuscript (e.g. S13). Please either refer to them or remove them from the supporting information.
Response: These figures were referred in the text of supplementary information.

Question 13. What is the purpose of the supporting video? I struggle a bit to see the point. It would be more interesting to see H_2 evolution during irradiation.
Response: We are intended to show the generation of gas in the reactor. We have removed this video.

Thinks for the valuable comments and suggestions. It is helpful for us to improve this manuscript.

Reviewer: 3

This manuscript by Wang et al describes the photoreforming of biomass-derived molecules into methanol and Syngas, thereby creating useful feedstocks from an abundant resource. This is undertaken using TiO_2 nanorods loaded with Cu, which work efficiently when irradiated with high-intensity UV light. The observed activity is impressive compared to other photocatalysts for the reaction, however the discoveries do not represent the significant advance in knowledge required for publication in Nature Communications. The system relies predominantly on well-studied components and reactions and the report focuses on their optimisation towards high activity and selectivity. I therefore believe the data would be best suited to a more specialist journal.

Question 1. What power of light is hitting the sample during the experiment? It

appears that the intensity is high and only UV-light is implemented. How does the system work under simulated solar light?

Response: The 365 nm LED light intensity is 55 mv/cm^{-2}. We have conducted the reaction under solar light irradiation (Figure R7). The reactor was put under the sunshine four hours every day. Although the intensity of UV light in the solar light is in a minor amount, the reaction also proceeded but with lower efficiency compared with the 365 LED light. After reaction for 16 h, 4% yield of methanol was produced along with a generation of 0.8 mmol g_{cat-1} h_{-1} of H_2.

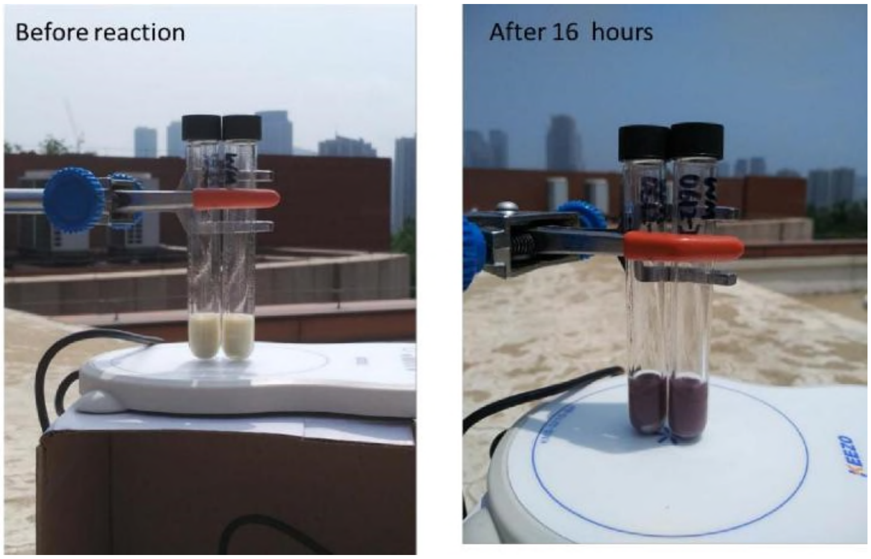

Figure R7 Photoreforming of glycerol in the under solar light. Reaction conditions: 10 mg of glycerol, 0.8 mL of MeCN, 0.2 mL of water, Ar, 16 h.

Question 2. The mechanism involving a semiconducting CuOx on the surface of TiO_2 is not sufficiently justified. If such a tandem light absorbing mechanism was occurring, it should be visible in the UV-visible spectrum of the photocatalyst.
Response: The Cu/TNR can adsorb visible light, but the intensity is lower than that in the UV region. As shown in the UV-visible spectrum of the photocatalyst (Figure R8), peaks at 400–550 and 650–800 nm are assigned to CuOx (Appl. Surf. Sci. 2016, 389, 760). The intensity increases with the increase of copper content.

But under visible light irradiation (455 nm LED), the reaction is very slow. After irradiation under 455 nm LED light for 12 h, only a very weak peak of CO_2 was observed (Figure R9). This is probably that the visible light can only excite the CuOx, and the holes and electrons are easily recombined. We studied the charge separation efficiency of Cu/TNR under UV and Visible light irradiation. The photocurrent under UV light irradiation is much higher than that under visible light irradiation (Figure R10a). Moreover, time resolved photoluminescence spectroscopy also showed that the lifetime of charge carriers under UV light irradiation is longer than that under visible light irradiation (Figure R10b). Therefore, Cu/TNR can adsorb visible light but show low activity in the photoreforming of glycerol due to the low separation efficiency of

charge carriers.

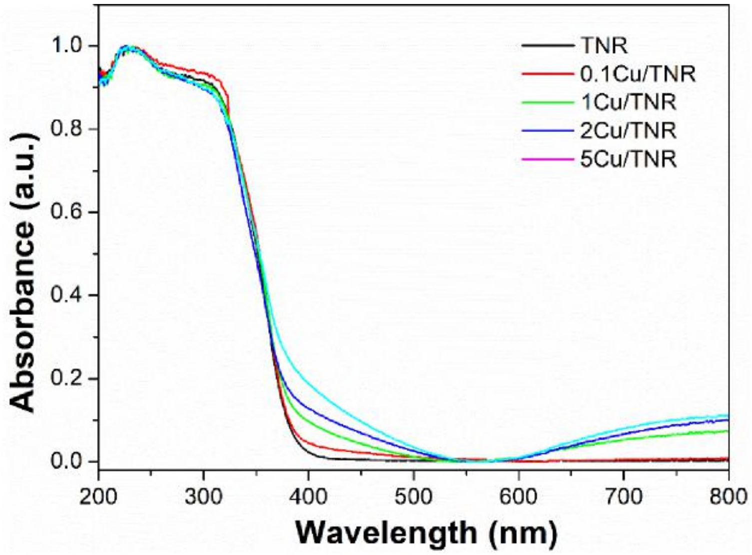

Figure R8 UV Vis spectra of Cu/TNR catalyst

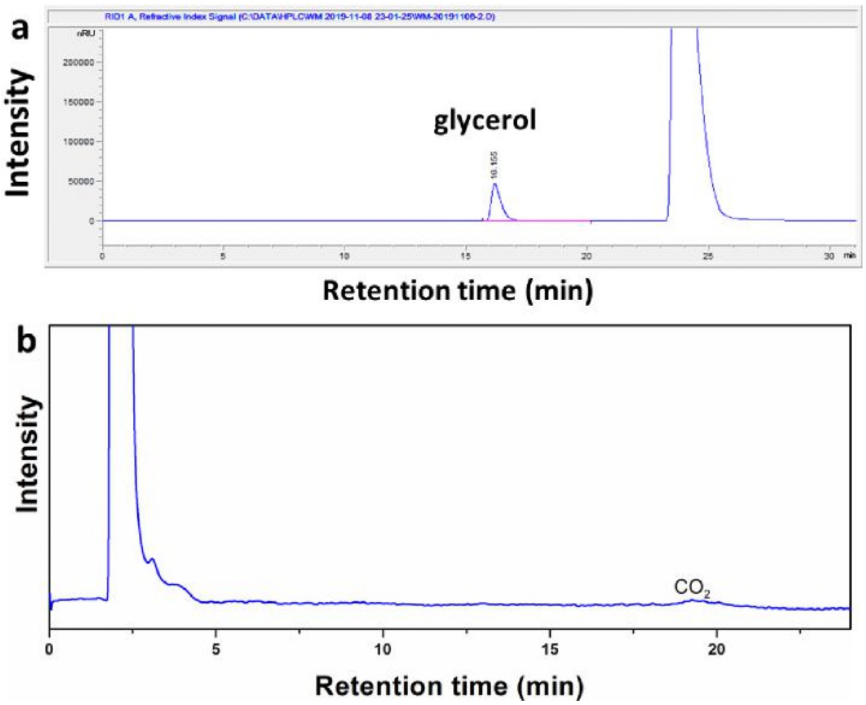

Figure R9 The HPLC (a) and (GC) curves of photoreforming of glycerol with visible light (455 nm LED). Reaction conditions: 10 mg of glycerol, 0.8 mL of MeCN, 0.2 mL of water, 455 nm LED (18 W, 160 mv cm^{-2}), Ar, 12 h

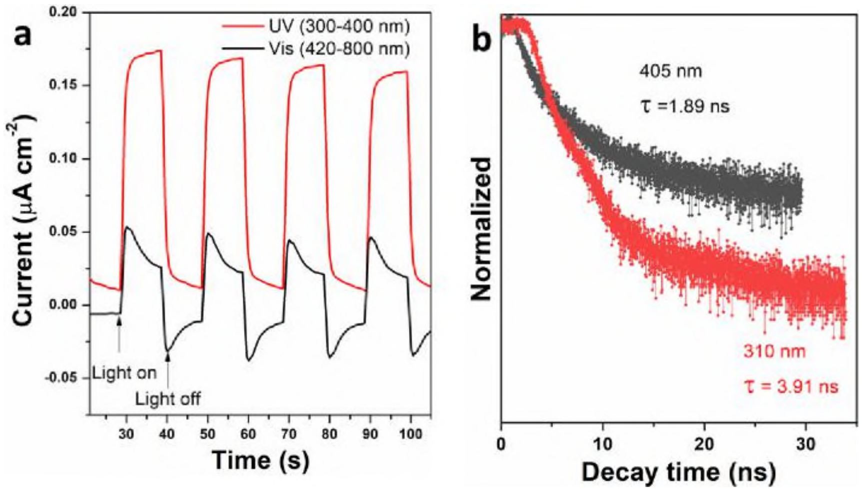

Figure R10 Photocurrent (a) of 2Cu/TNR irradiated with a UV (270 mv cm^{-2}) and Vis light (360 mv cm^{-2}) and time resolved photoluminescence spectroscopy (b) of 2Cu/TNR irradiated with a UV (310 nm) and Vis (405 nm) light.

Question 3. In Figure 3a what solvent conditions were used when varying the Cu quantity?

Response: We have shown the reaction conditions in the caption of Figure 3. For the investigation of copper loading effect, 0.95 mL of MeCN and 0.05 mL of water was used as solvent.

Question 4. Hydroxyl radicals are often referred to in order to explain the oxidation mechanism on the TiO_2, however these statements are not referenced. Since there is some controversy concerning the presence of such radicals, these statements must be justified.

Response: Thanks for the reviewer' advices. We have cited the related reference in the revised manuscript. We have detected the hydroxyl radical using EPR, and 5,5-dimethyl-1-pyrroline N-oxide (DMPO) was used to capture the hydroxyl radicals. In water solvent, the signals of hydroxyl radicals were detected. When decreasing the water content to 5%, no obvious signals of hydroxyl radicals were observed, indicating that that decreasing the water concentration in the water-organic solvent system will reduce the hydroxyl radicals. The oxidation of organic species by hydroxyl radicals leads to the formation of carboxylic acids which decomposes to CO_2 via decarboxylation (Appl. Catal. B: Environ. 2011, 106, 681.). The reduce of hydroxyl radicals slows down the degradation of the organic species to CO_2.

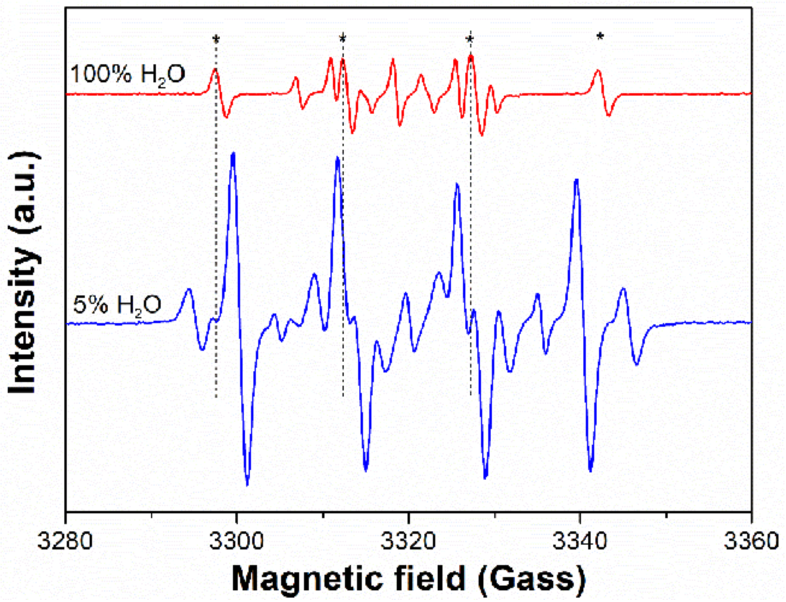

Figure R1 EPR spectra of hydroxyl radical (*) produced by photo irradiation over 2Cu/TNR and stabilized by 5,5-dimethyl-1-pyrroline N-oxide (DMPO).

Question 5. Could the Cu be doing CO_2 reduction to produce CO? Cu is a well-known CO_2 reduction catalyst

Response: Cu catalyst has been reported for CO_2 reduction. We used ^{13}C labelling experiments to study the source of CO. The photoreforming of glycerol was carried out in the presence of $^{13}CO_2$. $^{13}CO_2$ (1 mL) was injected in the reactor before reaction. Two catalysts with high copper loading (2Cu/TNR) and low copper loading (0.1 Cu/TNR) were studied. After reaction, the gas phase was analyzed by mass spectroscopy. A minor amount of ^{13}CO was generated. The results demonstrated that Cu/TNR was active for the photoreduction of CO_2 to CO, and the CO is dominantly produced from the direct decomposition of the organic intermediates.

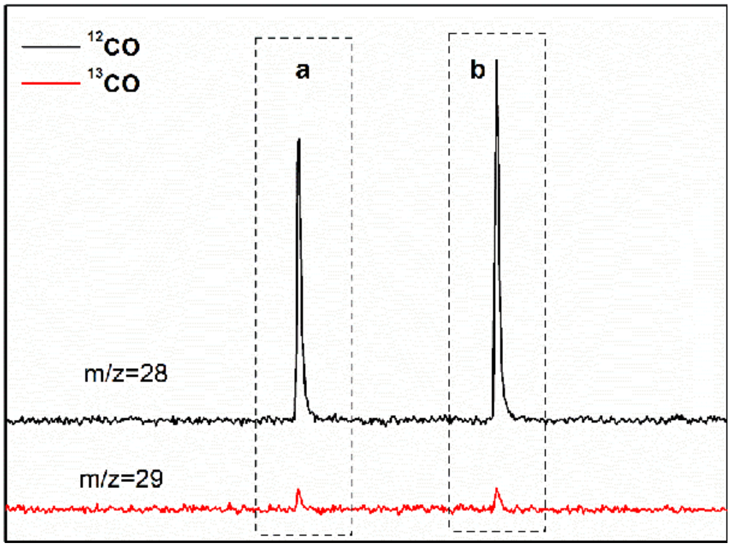

Figure R5 The mass spectroscopy of selected m/z=28, 29. Reaction conditions: (a) 10 mg of 2Cu/TNR, 10 mg of glycerol, 0.8 mL of MeCN, 0.2 mL of water, 365 nm 9 W LED , 1mL $^{13}CO_2$, Ar atmosphere, 12 h; (b) 10 mg of 0.1Cu/TNR, 10 mg of glycerol, 0.95 mL of MeCN, 0.05 mL of water, 365 nm LED (18 W, 55 mv cm^{-2}) , 1mL $^{13}CO_2$, Ar atmosphere, 24 h

Question 6. A lot of contemporary references on biomass oxidation are missing (see doi.org/10.1016/j.ccr.2015.12.009 and doi: 10.1002/anie.201710133 for examples).
Response: We have cited these two reviews in the manuscript.

Question 7. In summary the study is well carried out but the new hypotheses are not sufficiently justified and the catalyst not sufficiently novel for publication in Nature Communications. In terms of readability the English should be improved before publication, as the syntax is often irregular. I recommend the authors strengthen their mechanistic studies with further experimental evidence and submit this work to a more specialised journal.
Response: Methanol is recognized to be the most promising clean and large scale deployment liquid fuel in the future and also a fundamental chemical material and has been industrially used for the production of ethylene and propylene. Currently, methanol is industrially produced from natural gas and coal. The production of methanol from biomass instead of from fossils is a promising route. However, it has not yet been realized as it is a challenge to only break up all the robust C−C bond but not affects the C−H/C−O bonds. In the present work, we reported a light driven process to obtain methanol or syngas ($CO+H_2$) from biomass resource under room temperature, and the bio-syngas can be further used for the synthesis of methanol. We believe it is an important work. According to the reviewer's valuable comments and suggestions, we have improved the manuscript and strengthened the mechanism in the revised manuscript. We have tried out best to improve English. If necessary, we will seek a language editing service to improve the English before publication.

Thinks for the valuable comments and suggestions. It is helpful for us to improve this manuscript.

Reviewers' comments:

Reviewer #1 (Remarks to the Author):

The revised manuscript has been improved and it is acceptable.

Reviewer #2 (Remarks to the Author):

The authors have provided a much improved manuscript. A few minor points require addressing before this can be accepted:

• General comment: It would be very helpful if the authors indicate in the rebuttal letter where exactly the changes they made can be found in the manuscript (page and line numbers).
• P1, l13. The authors still claim that cellulose and raw sawdust were converted at room temperature, even though high temperature/pressure H2 are needed to pre-process. This claim is not justified and must be removed or corrected by adding something like "after high-temperature/high pressure pre-treatment". Similarly, Figure 1 still has a direct arrow from cellulose to light-driven conversion into syngas and MeOH. This is misleading and must be changed, e.g. adding "processing".
• The authors have added information about light intensity, however it is reported as "mv cm-2". Is this just a typo? Please convert this to mW cm-2.
• The authors have added compelling evidence that OH radicals are involved, however the explanations of the solvent effects are still a bit confusing. Fig. S2: The authors claim that CO2 formation is mostly a result of water present. However, there is not an awful difference between the amount of CO2 formed when pure water is used compared to 80% or even 90% MeCN. In addition, the EPR spectrum in fig. S4 actually shows a higher abundance of trapped OH radicals than in pure water. In my view, this seems to contradict the assumption that MeCN suppresses OH radical formation and thus suppresses over-oxidation. I appreciate the authors adding control experiments to study degradation of MeCN, although I would have preferred more solid evidence based on isotopic labelling. In any case, the performed control experiments should be mentioned in the manuscript.
• Although the authors have added AQY measurements to the SI this is not mentioned in the manuscript nor discussed by comparison with the state of the art.
• For comparison with other work, the authors have simply added a table to the SI listing previous work on glycerol photoreforming and two reviews. This is not a suitable replacement for a proper discussion of their results in the context of the state of the art. There is also quite a bit of work on direct photocatalytic conversion of cellulose that might be worth citing in this context, especially since some of these gave organic oxidation products beside CO2, though no MeOH (e.g. Chem. Commun. 2016, 52, 1673–1676; Nat. Energy 2017, 2, 17021; Appl. Catal. A 2018, 563, 73-79; J. Am. Chem. Soc. 2018, 140, 11604-11607). There is also precedent for photoreforming of pre-treated biomass (Am. J. Ener. Res. 2015, 3, 1–7).
• I find it quite remarkable and very interesting that the authors observe CO2 reduction to CO combined with biomass oxidation. Just to double-check: Can the authors rule out that 13CO peak not just a result

of the natural abundance of 13C in glycerol (this should be obvious from integration of the peaks)? Could the authors add a GC-MS trace when this experiment is performed under 12CO2 for comparison?

Reviewer #3 (Remarks to the Author):

The authors have clearly made substantial efforts to improve the manuscript and their responses have adequately answered the posed questions. Although the science is sound, I still have some reservations with respect to the novelty of the data, especially considering the much lower activity of the system when under solar irradiation. This should be acknowledged explicitly in the text.

I commend the authors on their extensive work on this publication and good quality science. Some further work will also be necessary to improve the English in the text before publication.

Response to the reviewers

Reviewer: 1

Reviewer #1 (Remarks to the Author):

The revised manuscript has been improved and it is acceptable.

Reviewer: 2

The authors have provided a much improved manuscript. A few minor points require addressing before this can be accepted:

Question 1. General comment: It would be very helpful if the authors indicate in the rebuttal letter where exactly the changes they made can be found in the manuscript (page and line numbers).

Response: Thanks for your suggestions. We accepted the reviewer's suggestion.

Question 2. P1, l13. The authors still claim that cellulose and raw sawdust were converted at room temperature, even though high temperature/pressure H2 are needed to pre-process. This claim is not justified and must be removed or corrected by adding something like "after high-temperature/high pressure pre-treatment". Similarly, Figure 1 still has a direct arrow from cellulose to light-driven conversion into syngas and MeOH. This is misleading and must be changed, e.g. adding "processing".

Response: We revised this sentence as "we report the conversion of biomass-derived polyols and sugars into methanol and syngas ($CO+H_2$) via UV light irradiation under room temperature, and the bio-syngas can be further used for the synthesis of methanol. The cellulose and even raw wood sawdust could be converted into methanol or syngas after hydrogenolysis or hydrolysis pretreatment." The changes were highlighted at page 1, line 12-16. We also modified Figure 1 and removed the "cellulose".

Question 3. The authors have added information about light intensity, however it is reported as "mv cm-2". Is this just a typo? Please convert this to mW cm^{-2}.

Response: The light intensity is mW cm^{-2}. We have corrected this typo.

Question 4. The authors have added compelling evidence that OH radicals are involved, however the explanations of the solvent effects are still a bit confusing. Fig. S2: The authors claim that CO_2 formation is mostly a result of water present. However,

there is not an awful difference between the amount of CO2 formed when pure water is used compared to 80% or even 90% MeCN. In addition, the EPR spectrum in fig. S4 actually shows a higher abundance of trapped OH radicals than in pure water. In my view, this seems to contradict the assumption that MeCN suppresses OH radical formation and thus suppresses over-oxidation.

Response: Thanks for the reviewer's comments.

For the trapped hydroxyl radicals, the distance between the peaks are equal and the peak intensity follows the ratio of 1:2:2:1 (Arch. Biochem. Biophy. 1997, 347, 45– 52; J. Phys. Chem. 1979, 83, 652-656). In water solvent, the marked signals are typical peaks of the hydroxyl radicals (Figure R1). When decreasing the water content to 5%, no obvious signals of hydroxyl radicals were observed. The detected peaks are not trapped hydroxyl radicals because all the peaks do not follow the above-mentioned rules. It is also possible that the weak peaks of trapped hydroxyl radicals are overlapped by other peaks. From the EPR result, the content of hydroxyl radicals in water is higher than that in MeCN rich solvent.

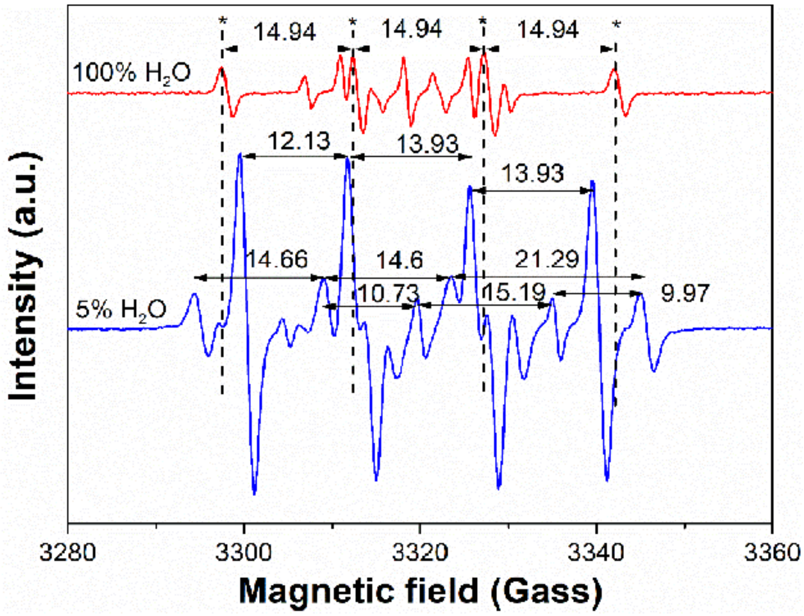

Figure R1 Analysis of the EPR result

The solvent effect is complex. Based on our results, we think the effect of solvent is probably to suppresses the formation of hydroxyl radicals. The oxidation of organic species by hydroxyl radicals leads to the formation of carboxylic acids which decompose to CO_2 via decarboxylation (Appl. Catal. B: Environ. 2011, 106, 681). We think the hydroxyl radicals are the major cause for over oxidation of the organic species to CO_2. Therefore, the competitive oxidation of water and polyols by the holes affect the final product in the water-organic solvent system. The hydroxyl radicals are formed via the hole oxidation of water. Decreasing water content will reduce the availability of water to the holes on the catalyst surface, and thus suppresses the formation of OH radicals and prohibits the degradation of organic species to CO_2.

The reaction rate varies in different solvent. The conversion of glycerol in water and MeCN-H_2O (8:2) solvent is 45% and 80%, respectively. It is better to compare

the selectivity but not the absolute amount of CO_2. As shown in Figure S2, the selectivity of CO_2 (69%) in water is higher than that (41%) in MeCN-H_2O (8:2) solvent. It can be deduced that the present of water favors the formation of CO_2.

Question 5. I appreciate the authors adding control experiments to study degradation of MeCN, although I would have preferred more solid evidence based on isotopic labelling. In any case, the performed control experiments should be mentioned in the manuscript.
Response: Isotopic reagent of $^{13}CH_3{}^{13}CN$ is currently unavailable for us. We have discussed the control experiments in the context (Page 5, line 4-8).

Question 6. Although the authors have added AQY measurements to the SI this is not mentioned in the manuscript nor discussed by comparison with the state of the art.
Response: We mentioned the AQY in the context (Page 5, line 1).

Question 7. For comparison with other work, the authors have simply added a table to the SI listing previous work on glycerol photoreforming and two reviews. This is not a suitable replacement for a proper discussion of their results in the context of the state of the art. There is also quite a bit of work on direct photocatalytic conversion of cellulose that might be worth citing in this context, especially since some of these gave organic oxidation products beside CO_2, though no MeOH (e.g. Chem. Commun. 2016, 52, 1673‑1676; Nat. Energy 2017, 2, 17021; Appl. Catal. A 2018, 563, 73-79; J. Am. Chem. Soc. 2018, 140, 11604-11607). There is also precedent for photoreforming of pre-treated biomass (Am. J. Ener. Res. 2015, 3, 1‑7).

Response: There are many literatures about the photoreforming of biomass and most of the works are focused on the generation hydrogen and CO_2. We have cited some typical references and discussed in the context (Page 4, line 1-6).

Question 8. I find it quite remarkable and very interesting that the authors observe CO2 reduction to CO combined with biomass oxidation. Just to double-check: Can the authors rule out that 13CO peak not just a result of the natural abundance of 13C in glycerol (this should be obvious from integration of the peaks)? Could the authors add a GC-MS trace when this experiment is performed under 12CO2 for comparison?

Response: After integration of the peaks, the content of ^{13}CO is 8.58% and 5.32% for 2Cu/TNR and 0.1Cu/TNR, respectively, which are higher than the natural abundance of ^{13}C (1.07%). When the reaction was carried out under ^{12}CO, no obvious ^{13}CO peak was detected by MS (Figure R2). The result indicates that the reduction of CO_2 to CO occurs during the biomass oxidation.

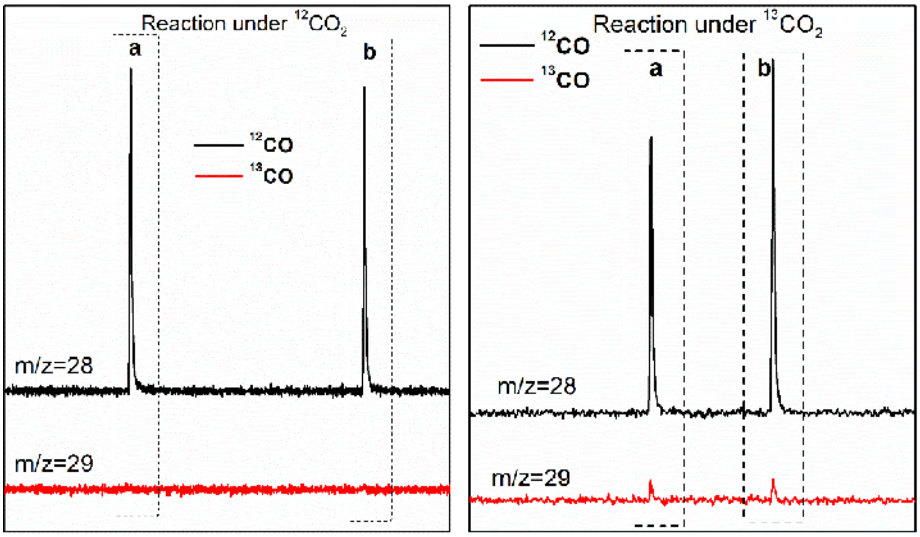

Figure R2 The mass spectroscopy of selected m/z=28, 29. Reaction conditions: (a) 10 mg of 2Cu/TNR, 10 mg of glycerol, 0.8 mL of MeCN, 0.2 mL of water, 365 nm LED (18 W, 55 mv cm^{-2}), Ar atmosphere, 12 h, 1 mL $^{13}CO_2$ or $^{12}CO_2$ of was injected into the reactor before reaction; (b)10 mg of 0.1Cu/TNR, 10 mg of glycerol, 0.95 mL of MeCN, 0.05 mL of water, 365 nm LED (18 W, 55 mW cm^{-2}), Ar atmosphere, 24 h, 1 mL $^{13}CO_2$ or $^{12}CO_2$ of was injected into the reactor before reaction.

Thinks for the valuable comments and suggestions. It is helpful for us to improve this manuscript.

Reviewer: 3

The authors have clearly made substantial efforts to improve the manuscript and their responses have adequately answered the posed questions. Although the science is sound, I still have some reservations with respect to the novelty of the data, especially considering the much lower activity of the system when under solar irradiation. This should be acknowledged explicitly in the text. I commend the authors on their extensive work on this publication and good quality science. Some further work will also be necessary to improve the English in the text before publication.

Response: Thanks for your comments. We have discussed the results under solar irradiation in the main text (Page 5, line 1-4). If necessary, we will seek a language editing service to improve the English before publication.

REVIEWERS' COMMENTS:

Reviewer #2 (Remarks to the Author):

I am happy with the revision. The manuscript should be accepted.

Moritz Kuehnel

Response to the reviewers

Reviewer: 2

I am happy with the revision. The manuscript should be accepted.

Response: Thinks for the valuable comments and suggestions. It is helpful for us to improve this manuscript.
